# Sulfation at Glycopolymer Side Chains Switches Activity at the Macrophage Mannose Receptor (CD206) In Vitro and In Vivo

**DOI:** 10.1021/jacs.2c10757

**Published:** 2022-12-06

**Authors:** Francesca Mastrotto, Marco Pirazzini, Samuele Negro, Alan Salama, Luisa Martinez-Pomares, Giuseppe Mantovani

**Affiliations:** † School of Pharmacy, University of Nottingham, Nottingham NG7 2RD, U.K.; ‡ School of Life Sciences, University of Nottingham, Nottingham NG7 2RD, U.K.; § Department of Pharmaceutical and Pharmacological Sciences, University of Padova, via F. Marzolo 5, Padova 35131, Italy; ∥ Department of Biomedical Sciences, University of Padova, Via Ugo Bassi 58/B, Padova 35131, Italy; ⊥ Department of Renal Medicine, University College London, London NW3 2PF, U.K.

## Abstract

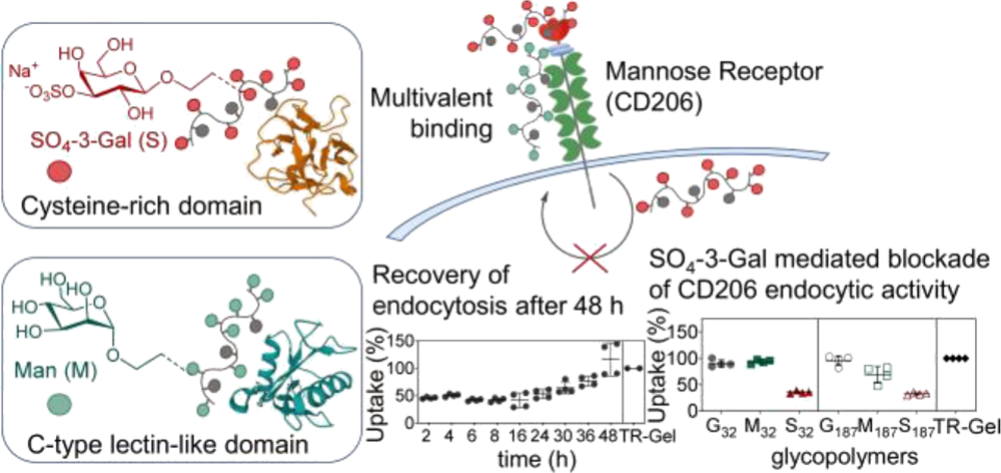

The mannose receptor (CD206) is an endocytic receptor expressed by selected innate immune cells and nonvascular endothelium, which plays a critical role in both homeostasis and pathogen recognition. Although its involvement in the development of several diseases and viral infections is well established, molecular tools able to both provide insight on the chemistry of CD206-ligand interactions and, importantly, effectively modulate its activity are currently lacking. Using novel SO_4_-3-Gal-glycopolymers targeting its cysteine-rich lectin ectodomain, this study uncovers and elucidates a previously unknown mechanism of CD206 blockade involving the formation of stable intracellular SO_4_-3-Gal-glycopolymer–CD206 complexes that prevents receptor recycling to the cell membrane. Further, we show that SO_4_-3-Gal glycopolymers inhibit CD206 both in vitro and in vivo, revealing hitherto unknown receptor function and demonstrating their potential as CD206 modulators within future immunotherapies.

## Introduction

Therapies involving immune blockade/modulation are revolutionizing the treatment of many cancers^1−3^ and are also being investigated for the treatment of both viral^4^ and bacterial^5^ infections, e.g., through host-directed therapies or modulation of the host immune response to infection.^4^ Within this context, chemical tools have been increasingly investigated to dissect and manipulate immune responses.^6,7^

The mannose receptor (MR, CD206) is an endocytic receptor that selectively binds an extensive range of both endogenous and exogenous ligands and is widely expressed by key immune cells such as tissue macrophages, selected populations of dendritic cells (DCs), and nonvascular endothelium.^8,9^ Its involvement in homeostatic clearance, antigen presentation, and modulation of cellular activation clearly places CD206 at the interface between innate and adaptive immunity.^9,10^ From a clinical perspective, CD206 plays a role in a range of diseases and conditions. In the context of viral infections, CD206 mediates dengue virus,^11^ HIV-1 infection of macrophages^11,12^ and entry of hepatitis B virus into intrahepatic DCs.^13^ We demonstrated its role in the development of kidney crescentic glomerulonephritis in mice,^14^ and in the recognition of a number of allergens and promotion of Th2 T cell differentiation,^15,16^ Imaoka and co-workers showed CD206 overexpression on alveolar macrophages in lungs of patients with severe chronic obstructive pulmonary disease and suggested its involvement in the pathogenesis of this condition.^17^ Moreover, robust expression of CD206 has been observed in tumor-associated macrophages (TAMs),^18−20^ major tumor-promoting immune cells in many solid tumors, where they induce immunosuppression, metastasis, and angiogenesis.^21,22^ Importantly, very recently, Rudloff and co-workers showed that conformational switch of CD206 in TAMs induced by a 10-mer synthetic peptide reprograms M2-like TAMs to an antitumor M1-like phenotype, suppressing tumor growth.^23^ Thus, CD206 could become an important biological target in therapies aiming at reprogramming immune responses mediated by macrophages and DCs or preventing CD206-mediated host infection by pathogens, if effective tools to modulate and better understand its chemistry and biological function can be identified.^24^ Structurally, CD206 is a type I transmembrane protein with two lectin ectodomains; hence, glycans would be ideal candidates to achieve this goal.

Lectin “deciphering” of sugar-encoded information, known as glycocode^25,26^ plays a critical role in both innate^27,28^ and adaptive^29^ immunity, and better understanding of lectin and glycan interplay in inflammation has led to new insights into human disease and development of pro- and anti-inflammatory therapeutics.^30^ However, the very structural diversity that makes protein–glycan interactions essential to all life forms also makes them very difficult to study.^31,32^ This can be ascribed to the intrinsic chemical complexity of glycans and the lack of template-driven or transcriptionally controlled biosynthesis.^31,33^ As a result, naturally occurring oligosaccharides often possess significant microheterogeneity; even when they can be isolated in sufficient amounts from biological sources, they typically cannot be used to probe the functional complexity of the glycocode due to lack of both chemical uniformity and accessible analytical tools to characterize fully their detailed structures.^31,33^ These challenges, however, also make glycans a largely untapped resource for both biological discovery and unforeseen therapeutic opportunities.^32^ Synthetic mimics of natural lectin-binding oligosaccharides have proven indispensable as chemical tools to link lectin structure with function.^31,34^ Individual monosaccharides bind lectin sugar-binding pockets with low affinities, with dissociation constants (*K*_D_) typically in the millimolar range.^35^ Multivalency can enhance both binding avidity and specificity,^36,37^ and multiple copies of sugar molecules displayed on larger ligands or biological surfaces interact with lectins, often themselves organized into oligomeric structures.^31,38,39^ Synthetic glycopolymers can function as effective multivalent mimics of natural lectin-binding oligosaccharides when engineered with appropriate topology, valency, and sugar orientation. Chen and co-workers showed that DC surface modification with specific glycopolymers successfully promoted T-cell activation.^40^ Other recent examples include their use to elucidate how integrins can promote cancer growth and survival,^41^ including glioblastoma,^42^ unveiling a key, sugar-mediated mechanism of cancer immunoevasion,^43^ reprogramming macrophages from tumor-associated immunosuppressive (M2) to inflammatory (M1) anticancer phenotype,^44^ and illustrating the effect of antigen structure on intracellular routing in DCs.^45^ Thus, these multivalent ligands may provide new opportunities for interrogating and controlling receptor functions and, ultimately, treat diseases.^39^

Here, we present two families of glycopolymer multivalent ligands displaying galactose 3-*O*-sulfate or mannose carbohydrates, which independently target either the cysteine-rich (CR) or C-type lectin-like domains (CTLDs) lectin ectodomains of CD206, respectively, and differentially modulate its endocytic activity. We identify a structure–function relationship for these multivalent ligands and show that their chemical and structural features—nature and number of carbohydrate recognition elements and polymer chain size—directly control both extent and duration of this modulatory effect. Our results also provide new insights into the chemistry of sugar–CD206 interactions and intracellular trafficking of the receptor. Crucially, we also unveil a previously unknown mechanism of inhibition of CD206-mediated endocytosis, mediated by the novel galactose 3-*O*-sulfate-based glycopolymers presented here, and we propose a molecular mechanism for receptor modulation/inhibition which we observed both in vitro and in vivo in a murine model. Thus, this work not only elucidates fundamental aspects of CD206 chemistry and biology but also introduces a family of synthetic multivalent probes which effectively regulate CD206-mediated processes, potentially opening the way for clinical exploitation of this receptor as a therapeutic target in clinical settings.

## Results and Discussion

### CD206: Glycopolymer Multivalent Ligands and Binding Modalities

**Figure 1:**
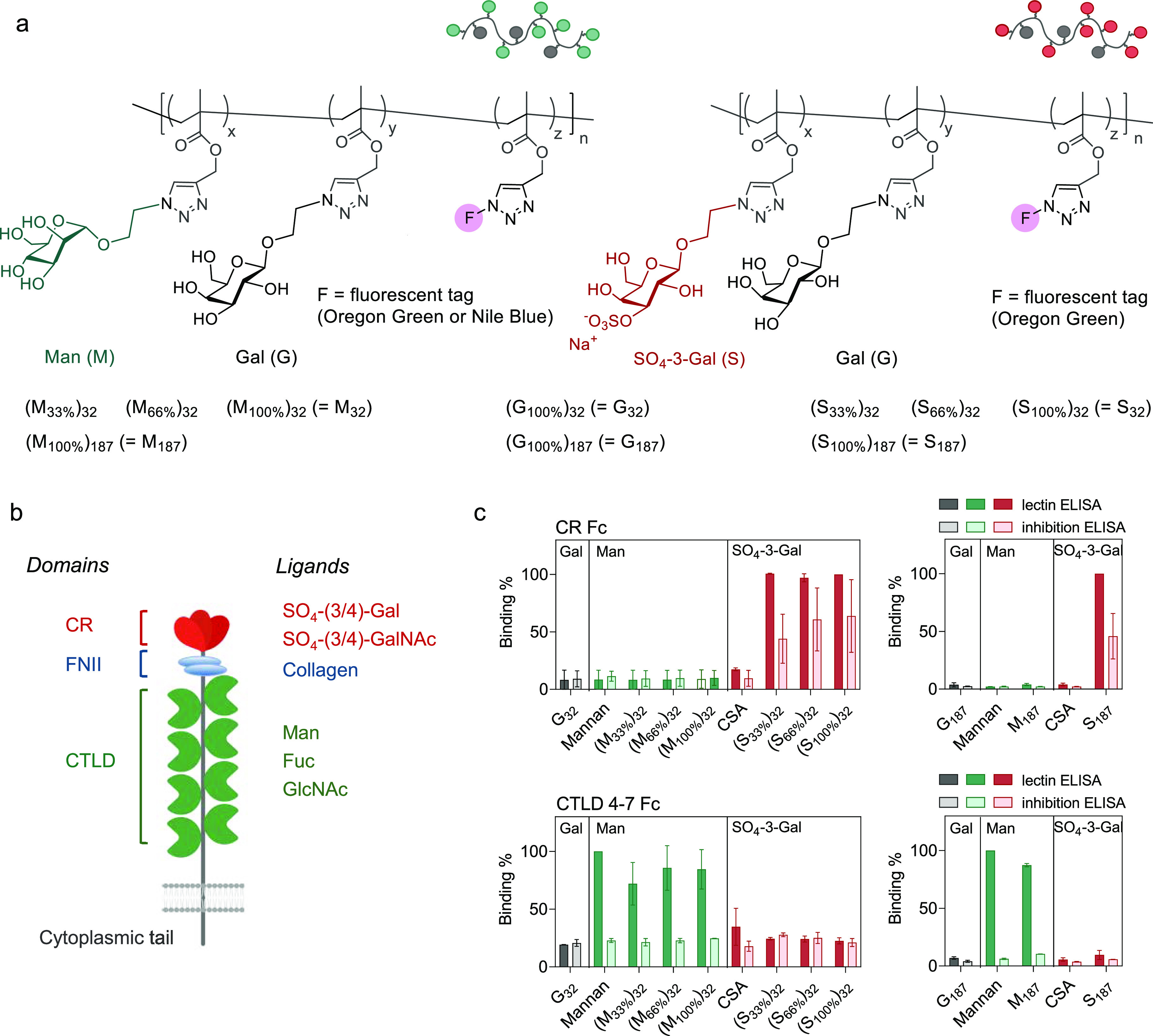
Mannose receptor (MR, CD206) glycopolymer ligands. (a) Structure of CD206-binding multivalent glycans designed in this study. These glycopolymer ligands possess variable chain length (*n* = 32 and 187) and either mannose (Man) or SO_4_-3-galactose (SO_4_-3-Gal) CD206-binding sugar units. Glycopolymer contents in Man (*x*) and SO_4_-3-Gal (*y*) are systematically varied (0, 33, 66, and 100%), using galactose (Gal) as the remaining non-CD206-binding polymer repeating units. Glycopolymers containing only non-CD206-binding Gal repeating units, G_32_ and G_187_, were used in this study as negative control ligands. All glycopolymers are fluorescently labeled with 1% mol/mol of either 2,4,5,7,7′-pentafluorofluorescein (Oregon Green (OG), λ_ex/em_ 488/530 nm) or Nile blue (λ_ex/em_ 629/670 nm) to enable detection in in vitro and in vivo assays. Table S1 shows the full composition and characterization of the glycopolymers. (b) Schematic representation of the mannose receptor (MR, CD206). CR: Cysteine-rich domain which binds 3/4-*O*-sulfate galactose (SO_4_-3/4)-Gal) and *N*-(acetyl galactosamine) (SO_4_-3/4)-GalNAc); FNII: collagen-binding fibronectin type II domain; CTLD: C-type lectin-like domain which binds mannose (Man), fucose (Fuc), and *N*-(acetyl glucosamine) (GlcNAc). (c) Binding of glycopolymers to CTLD4-7 and CR CD206 fragments as assessed by ELISA assay. Glycopolymers were first immobilized, and then for (i) lectin ELISA tests, they were treated with CTLD4-7-Fc or CR-Fc CD206 Fc-chimeras, while for (ii) inhibition ELISA tests, CTLD4-7-Fc and CR-Fc fragments were first co-incubated with 25 mM 2′-azidoethyl-*O*-α-D-mannopyranoside or 3-*O*-sulfo-2′-azidoethyl-*O*-β-D-galactopyranoside monovalent ligands and then added to the immobilized glycopolymers. In both sets of experiments, CD206 fragments were detected with anti-human IgG Fc-specific, alkaline phosphatase conjugates. G_32_ = (G_100%_)_32_, G_187_ = (G_100%_)_187_, M_187_ = (M_100%_)_187_, and S_187_ = (S_100%_)_187_. Results are from three independent experiments performed in quadruplicate. Data represent mean ± s.d. CSA: chondroitin sulfate A.

The initial part of this study involved the design of synthetic multivalent glycans able to recognize the different CD206 lectin domains (Figure 1a). CD206 is an endocytic receptor with three distinct binding ectodomains: (i) CR lectin domain that binds (SO_4_-3/4)-Gal and (SO_4_-3/4)-GalNAc sulfated sugars, (ii) collagen-binding fibronectin type II (FN II) domain, and (iii) CTLDs that recognize Man, Fuc, and GlcNAc carbohydrates in a Ca^2+^-dependent manner (Figure 1b).^9^ Thus, CD206 is unique among lectin receptors for possessing two independent lectin domains that bind remarkably different families of sugar ligands. Additionally, a tyrosine-based motif in the cytoplasmic intracellular tail directs the delivery of carbohydrate-containing ligands to early endosomes.^27^ Of the eight tandemly arranged CTLDs, only CTLD4-8 are believed to be directly involved in binding of carbohydrate ligands^46^—both endogenous and exogenous molecules, such as lysosomal hydrolases, allergens, and microbial-derived species—and only CTLD4 was shown to bind carbohydrates in isolation.^9^ FN II mediates collagen uptake, while the β-trefoil-shaped CR domain recognizes sulfated glycans in pituitary hormones such as lutropin and in lymphoid tissues and kidneys.^9^ The broad chemical diversity of CD206 ligands is perhaps surprising and reflects the multiple physiological roles of this receptor. The avidity of binding of glycopolymers to lectin receptors can depend on the polymer chain length,^47^ which also directly affects the ability of these multivalent ligands to span over multiple copies of receptors at cell membranes.^38^ To compare glycopolymers displaying different carbohydrate repeating units, it was therefore critical that they possessed the same average chain length. This can be achieved by synthesizing these libraries from the same parent polymer precursor, bearing reactive chemical handles which can be quantitatively functionalized with the required carbohydrate binding groups.^48^ Here, we followed a protocol previously developed by us and Haddleton,^49,50^ which involves sequential Cu^I^-catalyzed ATRP, to prepare a polymer precursor bearing reactive 1-alkyne repeating units, and “click” Huisgen 1,3-dipolar cycloaddition with specific sugar 2′-azidoethyl-*O*-glycosydes, to introduce 3-*O*-sulfo-*O*-β-D-galactopyranoside (SO_4_–3-Gal) and α-D-mannopyranoside (Man) sugar binding units (Scheme S1). During this second “click” step, a fluorescent tag, either Oregon Green or Nile Blue, is also introduced, to facilitate the detection of glycopolymers in subsequent in vitro and in vivo assays.

For the synthesis of these libraries of glycopolymers, three parameters were systematically varied, namely, (i) the nature of CD206-binding sugars (SO_4_-3-Gal vs Man), (ii) the number of CD206-binding sugars grafted on each polymer chain, and (iii) the length of the glycopolymer chains. Evidence indicates that the density of sugar ligands in a glycopolymer ligand can affect both binding kinetics and avidity to model lectins. In this study, glycopolymers were prepared with variable proportion—33, 66, and 100%—of SO_4_-3-Gal or Man repeating units, with the remaining sites being occupied by non-CD206 binding β-d-galactopyranose (Gal) molecules, to investigate the effect of these parameters on both binding to CD206 and uptake by CD206^+^ cells. As indicated in Figure 1a, for brevity, the codes for polymers where all repeating units are functionalized with the same sugar were simplified, i.e., (M_100%_)_32_ = M_32_, (S_100%_)_32_ = S_32_, etc. Glycopolymers were synthesized with degrees of polymerization (DP) of 32 and 187, corresponding to average molar mass *M*_n_ of 12–17 and 71–89 kDa, respectively. A DP of 32 was chosen for the library of glycopolymers with variable proportion of Man and SO_4_-3-Gal binding units, under the hypothesis that that their size was sufficient to elicit a significant multivalent effect, while still allowing an accurate estimation of their DP by ^1^H NMR, by comparison of the signals of polymer chain ends with those of the repeating units. Finally, glycopolymers with 100% non-CD206 binding Gal repeating units, G_32_ and G_187_, were also prepared and utilized as negative controls for subsequent in vitro and in vivo studies. We postulated that with appropriate choice of the sugar recognition elements in our glycopolymers, the two distinct lectin-type domains of CD206, CR and CTLDs, could be individually targeted with high specificity. This prediction was successfully validated by enzyme-linked immunosorbent assay (ELISA) tests using purified CR-Fc and CTLD4-7-Fc sub-fragments,^51^ which showed that the former could be targeted selectively with glycopolymers bearing SO_4_-3-Gal sugar units, and the latter with those containing Man units (Figure 1c).

### CD206-Dependent Cellular Uptake of SO_4_-3-Gal or Man Glycopolymers

**Figure 2:**
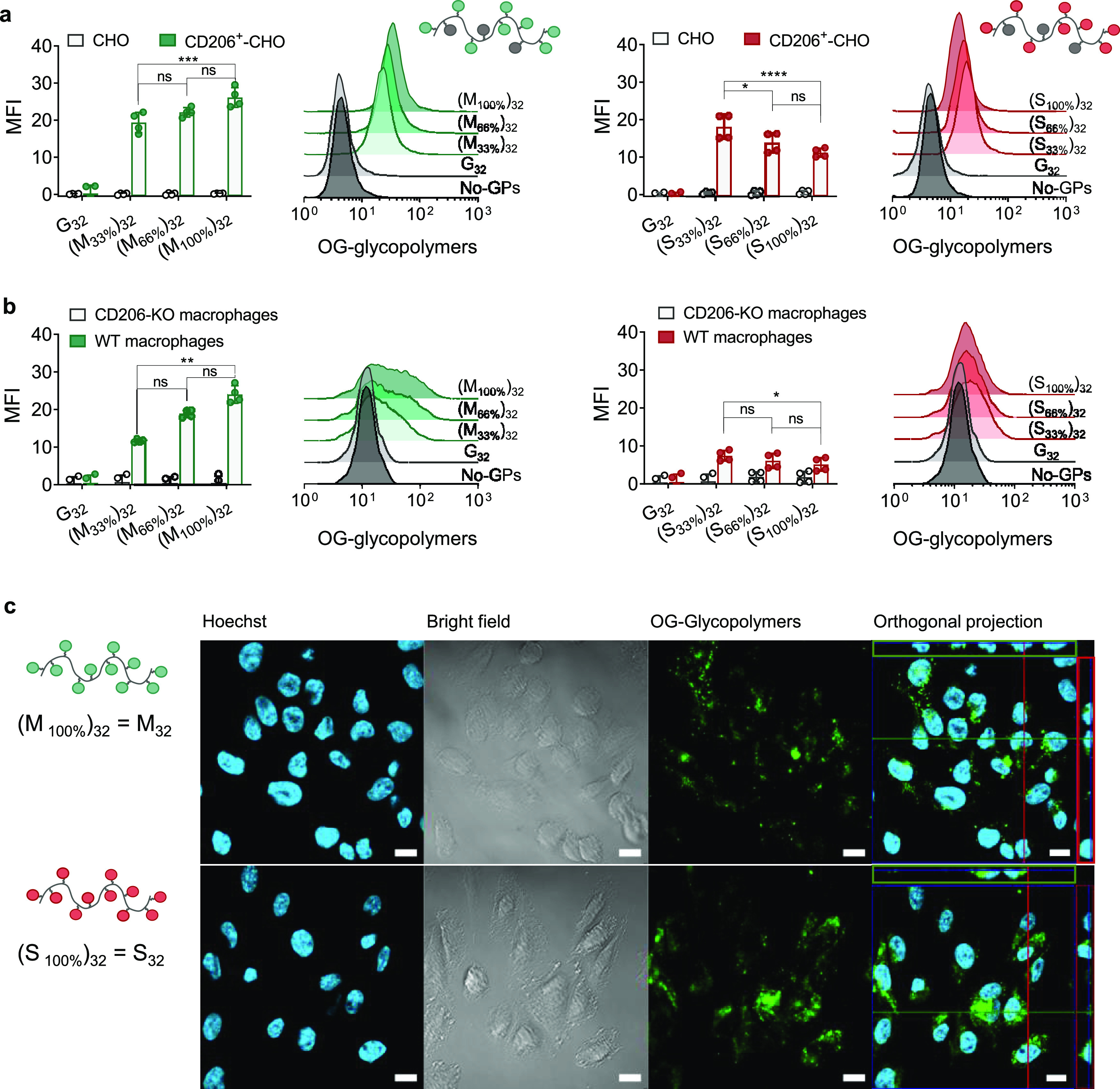
Internalization of SO_4_-3-Gal or Man glycopolymers by CD206-expressing cells. (a) Uptake of glycopolymers (GPs) with different contents of mannose (M) and 3-*O*-sulfo-galactose SO_4_-3-Gal (S) sugar repeating units by CD206^+^-CHO cells. Following incubation with Oregon Green (OG)-tagged glycopolymers (1.0 μg mL^–1^) for 30 min at 37 °C, cell uptake was quantified by flow cytometry. CD206^–^-CHO cells (CHO) were used as negative control. Data are presented as mean ± s.d. of four biological replicates from two independent experiments. (b) Wild-type (WT) macrophage uptake of glycopolymers (1.0 μg mL^–1^) after incubation for 30 min at 37 °C. CD206-knockout (CD206-KO) macrophages were used as negative control. Cell uptake was quantified by flow cytometry. Data are presented as mean ± s.d. of four biological replicates from two independent experiments. (c) Mannose (M) and 3-*O*-sulfo-galactose (S) glycopolymers are efficiently internalized by CD206^+^ cells. Representative images from confocal microscopy analysis of CD206^+^-CHO cells following incubation with Oregon Green-tagged glycopolymers (100 μg mL^–1^) for 1 h at 37 °C. Cell nuclei were stained with Hoechst, and imaging was done under bright field and fluorescence modes. MFI: median fluorescence intensity.

Next, CD206-mediated cellular uptake of these synthetic glycans was tested using CD206^+^-CHO cells and wild-type (WT) primary mouse macrophages, using the subfamily of glycopolymers with DP = 32, where the content of mannose and galactose-3-*O*-sulfate (SO_4_-3-Gal) was systematically varied. Efficient cellular internalization was observed in CD206^+^-CHO (Figure 2a) and WT macrophages (Figure 2b) for both Oregon Green-tagged Man- and SO_4_-3-Gal-glycopolymers, as confirmed by flow cytometry analysis.

Confocal microscopy analysis demonstrated that the OG-tagged glycopolymers were located intracellularly rather than bound to the cell membrane, thus confirming that the increase in fluorescence of CD206^+^-CHO was due to cell internalization, rather than simple association of glycopolymers to CD206 at the cell membrane without subsequent polymer endocytosis (Figure 2c). No cell uptake was observed by fluorescence-activated single cell sorting (FACS) flow cytometry analysis in CHO cells lacking CD206 and in CD206-deficient KO macrophages, or when non CD206-binding control glycopolymer G_32_ was employed, confirming that uptake of Man- and SO_4_-3-Gal-glycopolymers was indeed mediated by CD206. Here, a first clear difference between Man and SO_4_-3-Gal multivalent ligands became apparent. While mannosylated glycopolymer uptake by CD206^+^-CHO cells improved with the increasing number of Man units per polymer chain [(M_100%_)_32_ (=M_32_) > (M_66%_)_32_ > (M_33%_)_32_], the opposite was observed for SO_4_-3-Gal glycopolymers [(S_32%_)_32_ (=S_32_) > (S_66%_)_32_ > (S_100%_)_32_] (Figure 2a). WT macrophages displayed an analogous trend (Figure 2b). Uptake of mannosylated glycopolymers was time-dependent and, as expected, increased when incubation was prolonged from 30 to 60 min. Conversely, no increased internalization of SO_4_-3-Gal glycopolymers was observed at the 60 min time point (Figure S7a,b), indicating that glycopolymers internalized at the early stages of these experiments prevent further uptake of these ligands. Flow cytometry traces shown in Figure 2b are suggestive of the presence of macrophages with different levels of CD206 expression, more evident with Man-containing ligands due to their higher cell uptake compared to their SO_4_-3-Gal counterparts.

These initial observations suggested a modulatory effect of SO_4_-3-Gal glycopolymers on the endocytic activity of CD206. Importantly, at the range of concentrations (3.1–490 μM concentration of sugar repeating units) and incubation times (30–120 min incubation with glycopolymers and up to 48 h post-incubation in their absence) employed in the in vitro experiments in this work, all glycopolymers have low toxicity (>80% viability) for both CD206^+^CHO and BMDM cells, as assessed by LDH assay (Figures S4–S6).

### SO_4_-3-Gal- but Not Man-Containing Glycopolymers Inhibit the Endocytic Activity of CD206 In Vitro: Mechanistic Considerations

CD206 mediates clathrin-dependent endocytosis of ligands to endosomal compartments. Within endosomes, ligand–receptor complexes dissociate and CD206 recycles back to the cell membrane.^10^ At any given time, most of CD206 is intracellular,^52,53^ with very rapid endocytosis rates^54^ and a receptor turnover which for rat alveolar macrophages has been estimated to be ca. 11 min.^55^ In principle, the clustering of multiple receptor molecules at the plasma membrane induced by large multivalent ligands may be possible. However, a dedicated study by van Kasteren, Albertazzi, and co-workers using point accumulation in nanoscale topography super-resolution microscopy failed to show lowering of lateral diffusion of CD206 in the presence of multivalent ligands, and hence, ligand-induced CD206 clustering could be not be proven.^56^

**Figure 3:**
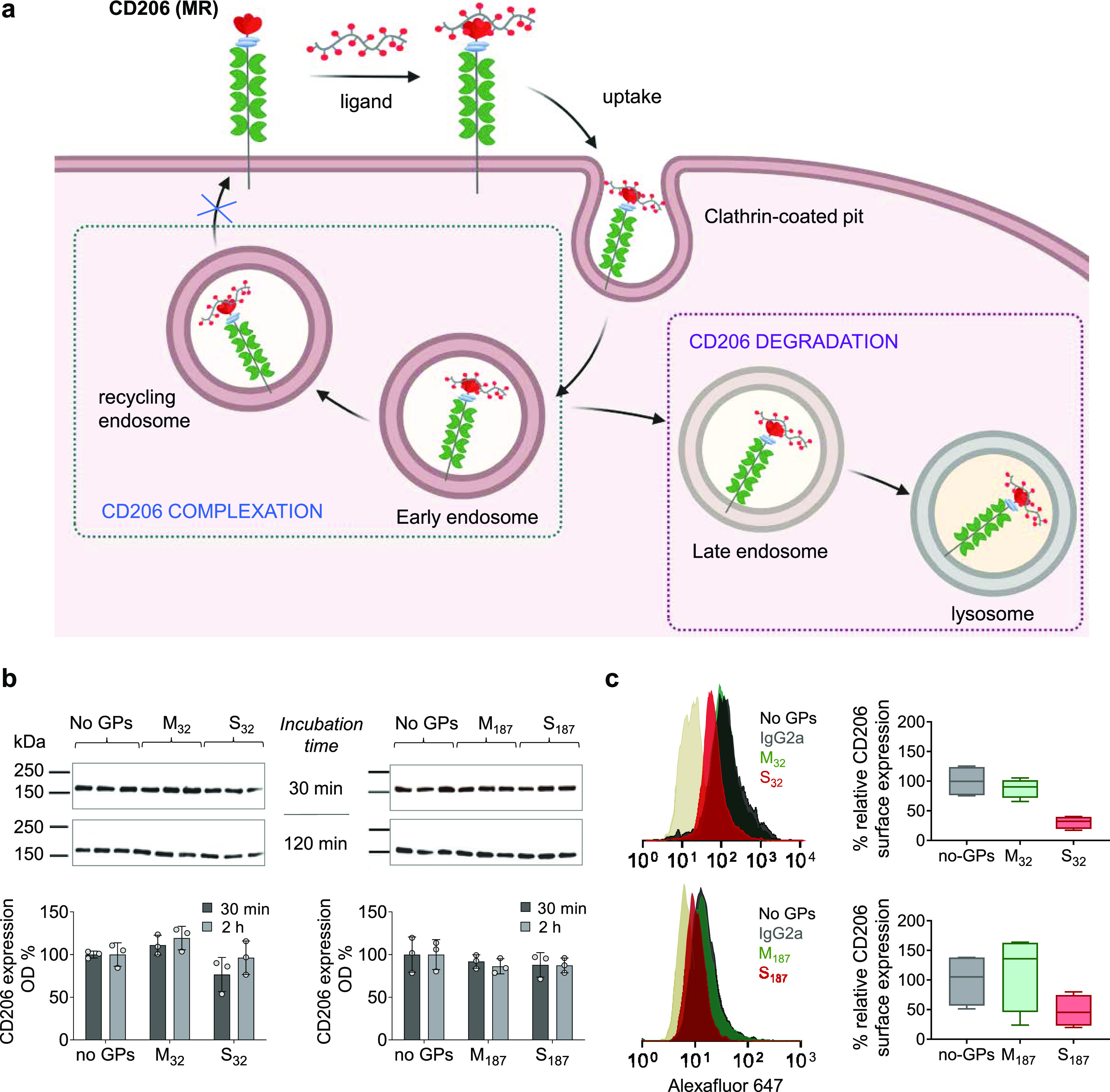
SO_4_-3-Gal glycopolymers inhibit recycling of CD206 to the plasma membrane. (a) Potential mechanisms by which SO_4_-3-Gal glycopolymers could inhibit CD206-mediated endocytosis: (i) modification of CD206 intracellular trafficking leading to receptor degradation (CD206 degradation) vs (ii) suppression of CD206 recycling to the plasma membrane due to the intracellular formation of stable (SO_4_-3-Gal glycopolymer)–CD206 complexes (CD206 complexation). For simplicity, potential clustering of multiple CD206 molecules at the plasma membrane prior to, or following, glycopolymer binding is not shown. Created with BioRender. (b and c) Both (b) total and (c) cell surface CD206 were quantified to establish whether the inhibition of CD206 endocytic activity occurs by an intracellular pathway leading to CD206 degradation or by forming stable glycopolymer–CD206 complexes that prevent recycling of the receptor back to the cell surface. (b) Total cell CD206 does not change following incubation with glycopolymers in the timescale of these experiments. Quantification of total cell CD206 in CD206^+^-CHO cells incubated with Man or SO_4_-3-Gal glycopolymers (490 μM concentration of sugar repeating units) for 30 or 120 min, compared with untreated CD206^+^-CHO cells (no glycopolymers (GPs)). CD206 expression was estimated by optical densitometry (OD) of western blot gels. Data are representative of three biological replicates. Full gels are shown in Figure S12. (c) CD206 at the cell surface decreases following treatment with SO_4_-3-Gal-glycopolymers S32 and S_187_ but not with Man-glycopolymers M_32_ and M_187_. Quantification of CD206 at the cell surface after treatment with Man or SO_4_-3-Gal glycopolymers (490 μM concentration of sugar repeating units) or the cell medium only for 120 min. Cells were harvested under nonenzymatic cell dissociation conditions. Membrane CD206 was immunostained with MR5D3 antibody, using rat IgG2a as isotype control, and quantified by flow cytometry. Box-and-whisker plots show a median (centerline), upper/lower quartiles (box limits), and maximum/minimum (upper/lower whiskers) of four biological replicates from two independent experiments. GPs = glycopolymers.

In our initial experiments, early cell uptake of SO_4_-3-Gal glycopolymers appeared to hamper further CD206-mediated endocytosis (Figure S7b). This phenomenon could be explained by two mechanisms: (i) re-routing of CD206 cell trafficking, resulting in receptor degradation (Figure 3a, CD206 degradation), or (ii) blocking of CD206 endocytic activity due to formation of very stable [SO_4_-3-Gal glycopolymer–CD206] complexes unable to dissociate within recycling endosomal compartments (Figure 3a, CD206 complexation), which would prevent the recycling of the free CD206 receptor to the cell membrane.

To test our first hypothesis, we estimated the variation of total cellular CD206 by western blotting of cell lysates following treatment of CD206^+^-CHO with S_32_ and S_187_ glycopolymers. Results indicate that the total amount of cellular CD206 remained virtually unchanged after 30- and 120-min treatment with glycopolymers compared to untreated CD206^+^-CHO control cells (Figure 3b), ruling out that the observed inhibition of CD206-mediated uptake was due to receptor degradation. Conversely, incubation with SO_4_-3-Gal glycopolymers significantly decreased the level of CD206 at the cell surface—down to 31 and 48% of its initial amount for S_32_ and S_187_, respectively—compared to untreated CD206^+^-CHO cells (Figure 3c). These findings suggest that following incubation with S_32_ and S_187_, a larger proportion of receptor might be retained intracellularly. Treatment of CD206^+^-CHO cells with mannosylated glycopolymers M_32_ and M_187_ did not change total and membrane CD206. Taken together, these results support the original hypothesis that sulfated multivalent ligands S_32_ and S_187_, unlike their mannosylated analogues, could inhibit CD206 endocytic activity by trapping the receptor intracellularly and preventing, at least for the time scale of these initial experiments, its recycling back to the cell membrane.

**Figure 4:**
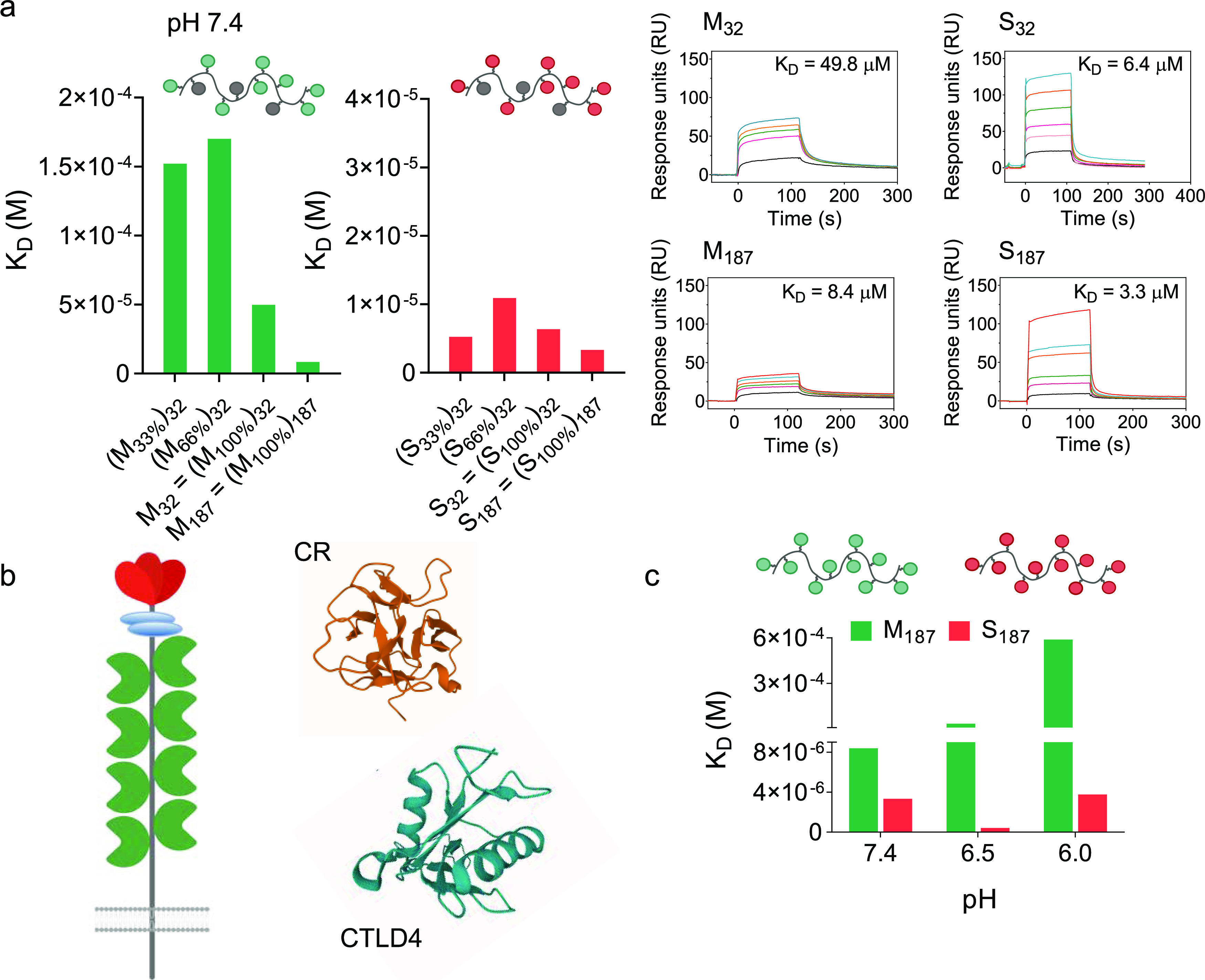
Differential pH dependency of mannosylated and sulfated glycopolymers binding to CD206. (a) Binding of Man and SO_4_-3-Gal glycopolymers to CD206 at pH 7.4, as assessed by SPR. Nature of sugar repeating units, their density along each polymer chain, and size of the glycopolymers are systematically varied, to identify a structure–activity relationship for these multivalent ligands. Representative SPR sensorgrams of binding profiles are shown for M_32_ and S_32_ and M_187_ and S_187_ at concentrations of polymer sugar repeating units of 0.032–2.4 mM. Sensorgrams of all polymers are shown in Figures S13 and S14. (b) Schematic representation of CD206, and crystal structures of its CR domain^59^ and CTLD4,^58^ PDB ID 1DQO and 1EGG from RCSB PDB, respectively. Images were produced with Mol*.^60^ (c) pH strongly affects binding of mannosylated M_187_ to CD206 but to a much lesser extent that of S_187_. Binding to CD206 is tested in the 7.4–6.0 pH range, to simulate the conditions which glycopolymer–CD206 complexes encounter when going from the cell membrane to endosomal compartments. Dissociation constant *K*_D_ values are derived from SPR analysis, using immobilized CD206, in 10 mM HEPES, 5 mM CaCl_2_, 0.005% tween-20, 150 mM NaCl, pH 7.4, 6.5, 6. Higher *K*_D_ values indicate more favorable dissociation of glycopolymer–CD206 complexes.

To explain these major differences in cellular uptake between the two families of glycopolymers, we sought to explore how the chemical nature and number of binding sugar units per polymer chain and size of glycopolymer affected the avidity of binding of the different glycopolymers to CD206 (CR and CTLD4 shown in Figure 4b), using surface plasmon analysis (SPR). Initial experiments showed that at pH 7.4 binding to mannosylated glycopolymers increased as the amount of Man units per polymer chain increased from 66 to 100% (Figure 4a and Table S2). Binding also increased with the polymer molar mass, with dissociation constants *K*_D_ of 5.0 × 10^–5^ and 8.4 × 10^–6^ M for M_32_ and M_187_, respectively. On the other hand, relatively small differences in *K*_D_ were observed for all SO_4_-3-Gal glycopolymers. Thus, this part of our study provided an initial structure–affinity relationship for these two families of multivalent CD206 ligands.

Using M_187_ and S_187_ as representative examples of Man and SO_4_-3-Gal glycopolymers, measurements were then repeated under conditions simulating those occurring when ligands bind the receptor at the cell membrane and, following cell uptake, are subsequently trafficked into increasingly acidic endosomal compartments. He and co-workers showed that, upon acidification, CD206 undergoes a transition from extended to more compact conformation and suggested that ligand binding and release depend not only on the binding affinities of individual domains but also on the interdomain conformation of the receptor as a whole, both of which are affected by the pH.^57^ We found that at pH 7.4, typical of the extracellular environment, the avidities of M_187_ and S_187_ for CD206 were comparable, both with *K*_D_ in the micromolar range (Figure 4c and Table S2). However, at more acidic pH, the binding avidity of mannosylated M_187_ dramatically decreased, with *K*_D_ increasing by one and almost two orders of magnitude at pH 6.5 and 6.0, respectively. In contrast, the binding of S_187_ remained essentially unchanged under these experimental conditions. Unlike the CR domain, binding of CD206 CTLDs to ligands is Ca^2+^-dependent. Work by Weis’s group suggests a mechanism for endosomal ligand release from CTLDs involving a pH-induced loss of Ca^2+^ ions and consequent conformational rearrangements of the receptor which makes it less capable of binding carbohydrate ligands,^58^ which is in agreement with our results. Conversely, the CR domain does not require Ca^2+^ for ligand recognition, and Bjorkman and co-workers showed that binding affinity to monovalent 4-SO_4_-GalNAc remains constant in the 4.5–7.5 pH range.^59^ Thus, our SPR data indicate that binding of CD206 to Man and SO_4_-3-Gal glycopolymers behaves very differently under conditions mimicking those encountered following receptor-mediated endocytosis, with CD206 largely losing its ability to bind Man glycopolymers as the pH decreases from 7.4 to 6.0, while binding is maintained for SO_4_-3-Gal glycopolymers.

**Figure 5:**
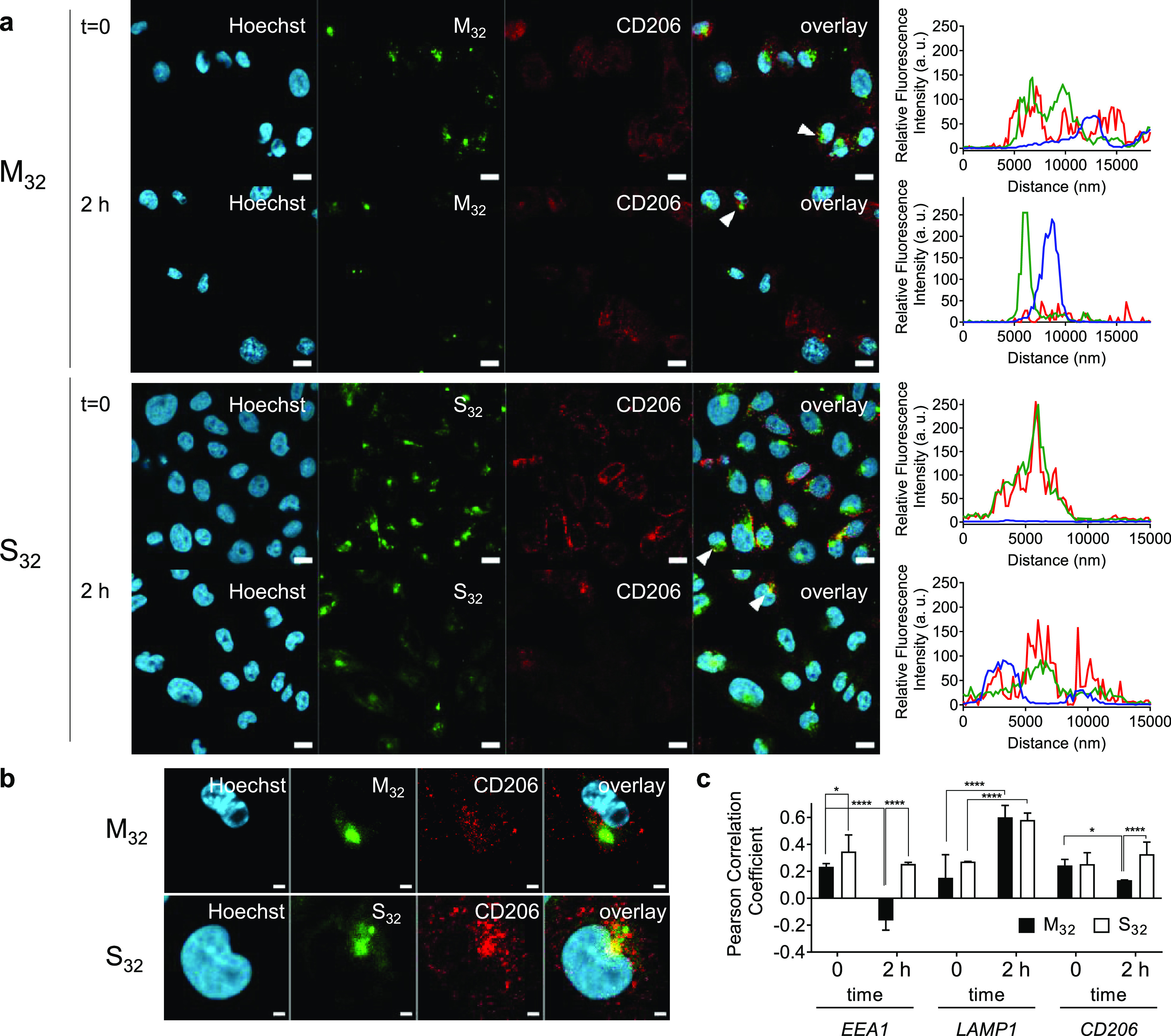
Intracellular trafficking of M_32_ and S_32_ glycopolymers. (a) Confocal images of CD206^+^-CHO cells incubated with M_32_ or S_32_ glycopolymers (259 μM concentration of sugar repeating units) for 2 h at 37 °C. Subsequently, cells were either collected (*t* = 0) or incubated for 2 h (*t* = 2 h) with a glycopolymer-free medium at 37 °C to allow intracellular trafficking of glycopolymers. In both instances, samples were processed for confocal microscopy and stained for early endosomes (anti-EEA1), lysosomes (anti-LAMP1), or CD206 (MR5D3, mannose receptor antibody). Graphs on the right show the normalized fluorescence intensity of line scans for Oregon Green-glycopolymers (green), CD206 (red), and Hoechst (blue) channels across cell areas indicated by the white arrows in the overlay images. (b) Magnification of individual cells indicated by white arrows in panel a for M_32_ and S_32_ at *t* = 2 h. (c) Pearson correlation r coefficients were calculated to assess the co-localization of the glycopolymers at different time points with early endosomes, lysosomes, or CD206. At *t* = 0, the glycopolymers M_32_ and S_32_ show similar co-localization with early endosomes (EEA1) and CD206. After 2 h post incubation, only S_32_ still partially co-localizes with both early endosomes and CD206 (*P* = 0.32 and 0.25, respectively), while M_32_ is not detected in EEA1-positive vesicles and has significantly lower (50%) co-localization with CD206. Both S_32_ and M_32_ increase their co-localization with lysosomes (LAMP1). Correlation coefficients were calculated from the analysis of at least 24 cells. Statistical significance was calculated with one-way ANOVA. Error bars indicate s.d. Panel a: scale bars 10 μm. Panel b: scale bar 2 μm. * *P* ≤ 0.05; **** *P* ≤ 0.0001.

To test whether differences in CD206 binding avidity for mannosylated and sulfated glycopolymers at low pH could translate into changes in intracellular trafficking of the glycopolymers following uptake by CD206^+^-CHO cells, we treated CD206^+^-CHO cells with M_32_ and S_32_ for 2 h (*t* = 0 h in Figure 5a), followed by incubation for further 2 h in a fresh polymer-free medium (*t* = 2 h, Figure 5a,b). After the incubation in the glycopolymer-free medium, fluorescence intensity line scans showed decreased co-localization of M_32_ and CD206 traces compared to S_32_ and CD206 traces, suggesting a closer proximity of intracellular CD206 and S_32_ (Figure 5a, right panels). Pearson correlation coefficients showed that co-localization between M_32_ and both early endosomes (EEA1^+^) and CD206 decreased significantly, while it increased with a marker of lysosomal compartments (Figure 5c). This is in line with the known mechanism of CD206-mediated endocytosis, where ligands are directed to lysosomal compartments, and receptor molecules continuously recycle back to the cell membrane.^53^ Conversely, S_32_ remained mostly colocalized with the CD206 receptor, as indicated by analysis of both the fluorescence intensity of line scans (Figure 5a) and Pearson correlation coefficients (Figure 5c), and was found to co-localize with both early endosomal and lysosomal markers. Thus, these results are in agreement with the distinct binding mechanisms identified for M_32_ and S_32_ in the SPR experiments and support the hypothesis that SO_4_-3-Gal glycopolymers form stable intracellular glycopolymer–receptor complexes, which can prevent CD206 recycling to the plasma membrane and inhibit its endocytic activity.

### SO_4_-3-Gal Glycopolymers Block CD206 In Vitro and In Vivo

Taken together, our data showed that an appropriate choice of glycopolymer multivalent ligands could induce the switching from continuous ligand delivery to CD206-expressing cells to inhibition of CD206-mediated endocytosis. The latter would be of particular interest as it may open the way to the development of CD206 blockers, with potential therapeutic implications for diseases and conditions where CD206^+^ DCs, macrophages, and endothelial cells play a major role.

To validate the CD206-blocking activity of SO_4_-3-Gal glycopolymers, we next explored whether these multivalent materials could inhibit uptake by CD206 ligands other than glycopolymers. Collagen was an obvious choice as its carbohydrate-independent binding to the FNII domain of CD206 and subsequent cell uptake have been extensively characterized, by us^51^ and Taylor et al.^61^ From a clinical viewpoint, it has been suggested that collagen uptake by TAMs mediated by two members of the mannose receptor family, CD206 and Endo180, could promote tumorigenesis through remodeling of the tumor extracellular matrix, and therapeutic interference with this pathway could be part of future anti-metastatic therapies.^62^

Initially, CD206^+^-CHO cells were pre-treated with either S_32_ or S_187_ as potential blockers (490 μM in sugar binding units), or analogous Man and Gal glycopolymers. After 30 min, the cell medium containing glycopolymers was replaced by a fresh medium containing Texas Red-tagged partially hydrolyzed collagen, gelatin (TR-gelatin, 10 μg mL^–1^), used as a fluorescent reporter of CD206-mediated endocytosis.

**Figure 6:**
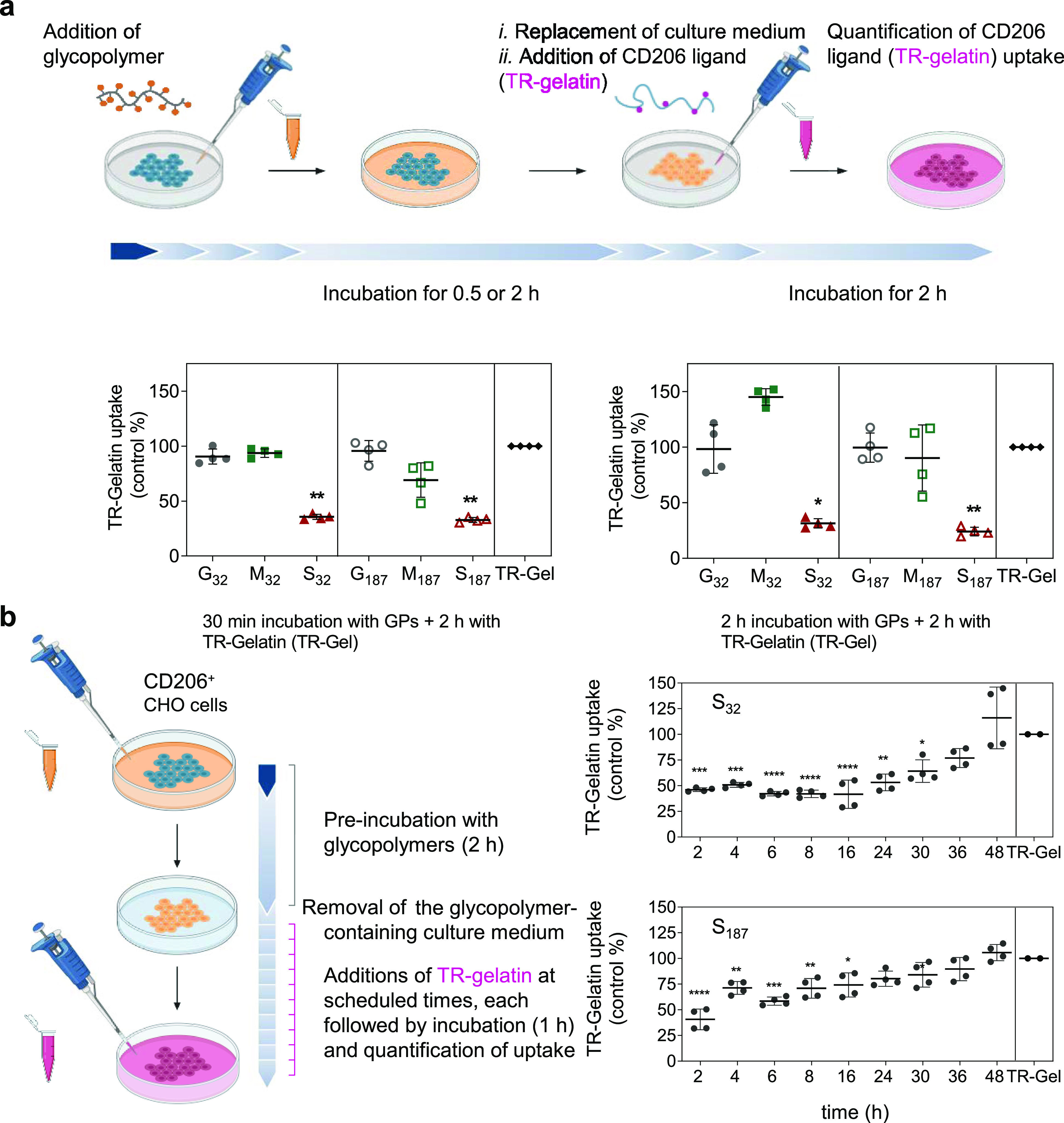
SO_4_-3-Gal glycopolymers inhibit CD206 endocytic activity in vitro. (a) SO_4_-3-Gal glycopolymers inhibit uptake of gelatin in CD206^+^-CHO cells. Cells were pre-treated with glycopolymers (GPs, 490 μM in sugar repeating units, 30 min, left panel, or 2 h, right panel), and then, the cells were washed and incubated with glycopolymer-free media containing Texas Red-tagged gelatin (TR-Gel, 10 μg mL^–1^), for 2 h. TR-gelatin uptake was quantified by flow cytometry. Data are expressed as a percentage of gelatin uptake compared to that of non-pre-treated cells (TR-Gel columns). Data are presented as mean ± s.d. of four biological replicates from two independent experiments. (b) Created with BioRender. Inhibition of CD206 endocytic activity by a single treatment with SO_4_-3-Gal glycopolymers is long-lasting yet reversible. CD206^+^-CHO cells were incubated for 2 h with S_32_ or S_187_ (490 μM in sugar repeating units). Control cells were incubated over the same period with a glycopolymer-free medium. After washing, cells were incubated with a fresh medium and at scheduled times treated with a pulse of TR-gelatin (80 μg mL^–1^) for 1 h. Gelatin uptake was quantified by flow cytometry. Data are expressed as a percentage of gelatin uptake compared to that of non-pretreated cells. Data are presented as mean ± s.d. of four biological replicates from two independent experiments. A one-way ANOVA was performed to test significance; *P ≤ 0.05, **P ≤ 0.01, **P ≤ 0.001, and **** *P* ≤ 0.0001. Created with BioRender.

**Figure 7:**
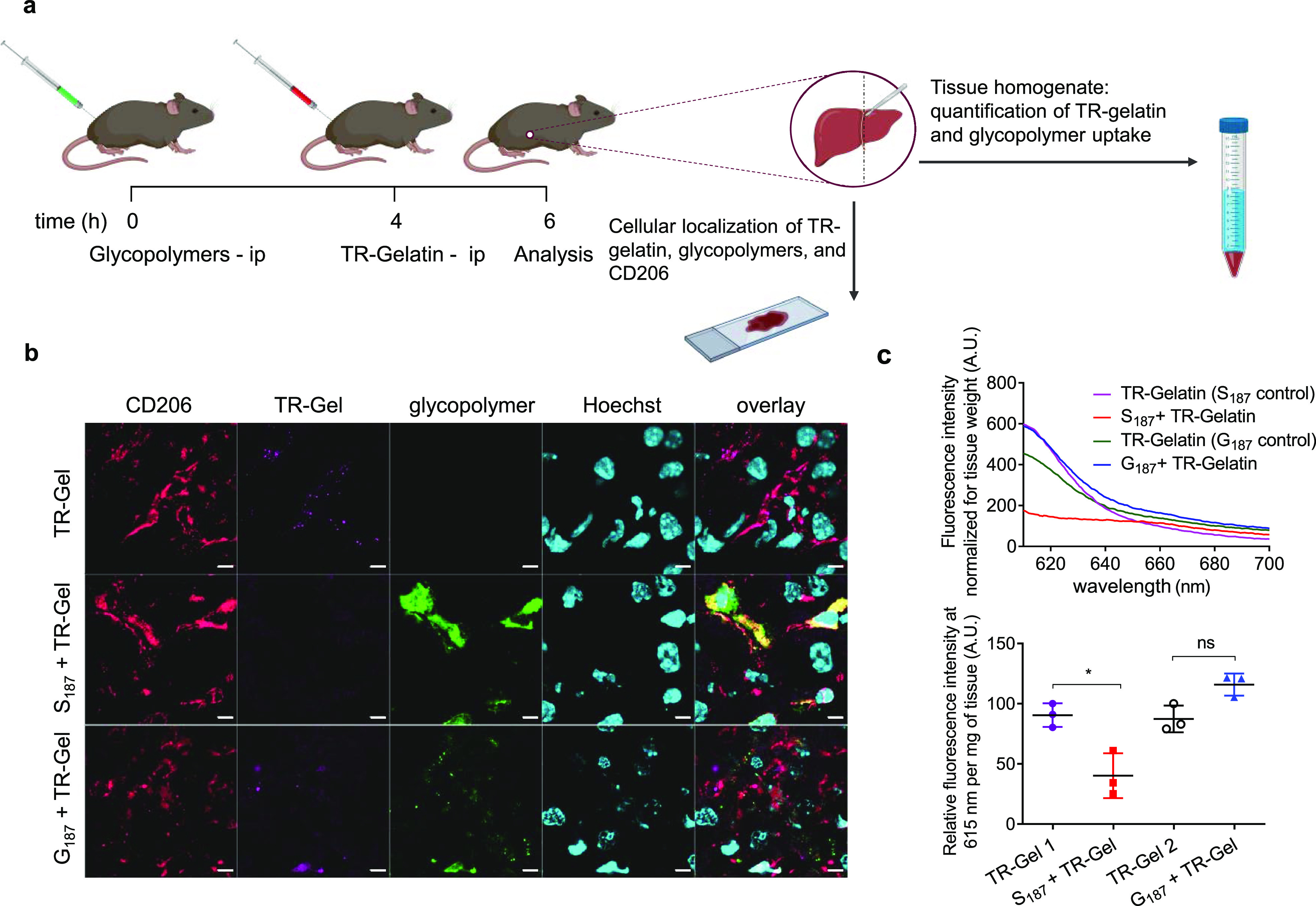
S_187_ inhibits uptake of gelatin by liver cells in vivo. (a) Mice were injected intraperitoneally with 100 μL of Oregon Green (OG)-tagged S_187_ or G_187_ (490 μM polymer units in PBS). Four hours later, mice received 100 μL of TR-gelatin solution (1.0 mg mL^–1^ in PBS), and livers were collected after further 2 h. Mice treated with TR-gelatin for 2 h were used as positive controls. Created with BioRender. (b) Immunofluorescence analysis of liver sections: representative two-dimensional fluorescence images obtained by confocal laser scanning with Hoechst staining for nuclei (cyano), immunostaining for the CD206 receptor (red), TR-gelatin (magenta), and S_187_ or G_187_ glycopolymers (green). Mice were treated with TR-gelatin only (TR-Gel) or S_187_ or G_187_ followed by TR-gelatin (S_187_ + TR-gelatin or G_187_ + TR-gelatin, respectively) as described in the Materials section. Scale bars: 5 μm. Series of 0.5 and 2× magnification images are shown in Figures S15–S17; and (c) liver TR-gelatin uptake was quantified on liver homogenate as relative fluorescence signal at λ_em_ = 615 nm per mg of liver tissue homogenate. Top panel: representative emission spectra in the 610 to 700 nm range (λ_ex_ = 596 nm for TR-gelatin quantification) from a single animal. Bottom panel, collated data λ_em_ = 615 nm from *N* = 3. TR-Gel 1 and TR-Gel 2 are the positive control samples (mice not pre-treated with glycopolymers) for S_187_ + TR-Gel and G_187_ + TR-Gel samples, respectively. A two-tailed t-test was performed to test significance; * *P* ≤ 0.05.

After 2 h of incubation, gelatin uptake was quantified by flow cytometry. Internalization of TR-gelatin was significantly reduced in cells pre-incubated with SO_4_-3-Gal glycopolymers S_32_ and S_187_, while cells treated with Man and non-binding Gal glycopolymers displayed a TR-gelatin uptake similar to that observed with untreated cells (Figure 6a). These uptake patterns did not significantly change by increasing the duration of polymer pre-incubation from 30 to 120 min (Figure 6a), or when bone-marrow-derived murine macrophages were used instead of CD206^+^-CHO cells (Figure S11). Very similar results were obtained when after the initial pre-incubation, glycopolymers were not removed and TR-gelatin was added in the culture media, thus obtaining a cell co-treatment assay (Figure S9). Finally, analogous data were obtained when a Nile Blue-tagged mannosylated glycopolymer M_32_, targeting CTLDs, instead of TR-gelatin (which targets the FN II domain) was used as the fluorescent ligand for CD206-mediated internalization (Figure S10). Thus, effective blockade of CD206-mediated endocytosis was demonstrated by using fluorescent probes targeting all three ligand-binding domains—CR (Figures 2a,b and S7), FN-II (Figures 6, 7, S9, and S11), and CTLDs (Figure S10).

Next, we performed time-course inhibition experiments to determine the duration of the inhibition of CD206 endocytic activity following a single treatment with SO_4_-3-Gal glycopolymers. CD206^+^-CHO cells were used for this proof-of-concept study to minimize the confounding factors that could arise from macrophages modifying their phenotype, including CD206 expression, during the course of the assay. Cells initially incubated with either S_32_ or S_187_ for 2 h and washed were treated at scheduled times with TR-gelatin for 1 h; changes in cell fluorescence were assessed by flow cytometry (Figure 6b). For both SO_4_-3-Gal glycopolymers, full CD206 endocytosis was restored after 48 h. S_32_ gave a persistent inhibition profile, with TR-gelatin uptake recovery starting after 24–30 h, while S_187_ followed a more linear profile. Recovery of uptake could be ascribed to a slow release of CD206 from the glycopolymer complexes followed by receptor recycling to the cell membrane, slight differences in intracellular trafficking, newly produced CD206 being displayed at the cell membrane, or a combination of these processes. Further investigation is needed to add insight into this potentially complex scenario and will be part of our future studies.

Collectively, these results indicate that SO_4_-3-Gal glycopolymers interfere with CD206 endocytic trafficking in a long-lasting, though reversible, manner. From a therapeutic and/or diagnostic viewpoint, this would be very convenient, as not only a sustained uptake inhibition would reduce the frequency of the required drug intakes, but a fully reversible effect could also ensure that CD206 activity is restored at the end of the treatment.

To investigate the translational relevance of our results, having demonstrated that SO_4_-3-Gal glycopolymers are efficient CD206 blockers in vitro, we sought to expand the scope of this technology to relevant in vivo models. Using glycosylated multivalent ligands in vivo is challenging, due to a plethora of different lectins having overlapping sugar ligand specificities, leading to off-target effects. For example, in addition to CD206, many other animal lectins recognize mannose-based molecular patterns, e.g., DC-specific intercellular adhesion molecule-3-grabbing non-integrin (DC-SIGN, CD209)^63^ and mannose-binding lectin.^64,65^ Selective targeting of CD206 in vivo using mannosylated ligands may therefore be challenging, unless disease-specific delivery strategies, e.g., local administration, are utilized. However, lectins that selectively recognize SO_4_-3-Gal motifs with high avidity are much less common. Using libraries of sulfated galactose-containing polymers prepared by ROMP polymerization, Kiessling and co-workers showed that for strong binding to P-selectin, an additional sulfate group at C6 of the galactopyranose ring was required.^66^ Using ligands specific for the CR domain based on SO_4_-3-Gal may therefore be a potential route for selective targeting of CD206 in vivo.

The potential of our SO_4_-3-Gal glycopolymers to inhibit CD206 endocytic activity in CD206^+^ hepatic cells in vivo was tested in a murine model. CD206 is the main receptor for collagen uptake in the mouse liver, due to the expression of CD206 in liver sinusoidal endothelial cells (LSECs) and, to a lesser extent, in resident macrophages, Kupffer cells.^67,68^ In the context of liver diseases, plasma levels of soluble CD206 (sCD206) have been utilized as a marker of liver disease severity and prognosis in patients with liver cirrhosis,^69^ alcoholic liver disease,^70^ and primary biliary cholangitis.^71^ Arteta et al. showed that in LSECs CD206-mediated endocytosis induces immunosuppression and development of liver metastases in a murine C26 colorectal cancer model and suggested that therapies directed at CD206 blockade may restore hepatic defense against metastatic colon carcinoma.^72^ Additionally, accumulation of intrahepatic TNFα-secreting CD206^+^ macrophages is associated with chronic liver inflammation and development of fibrosis and cirrhosis,^65^ while CD206^+^ tumor-infiltrating macrophages in hepatocellular carcinoma have been associated with elevated recurrence and reduced survival.^73,74^

For these experiments, we used S_187_ and non-CD206-binding G_187_ control polymers because their molar mass is higher than the 40–60 kDa threshold for kidney glomerular filtration for globular proteins,^75^ which would prevent relatively rapid renal excretion. S_187_ or G_187_ was administered to mice through the intraperitoneal route, followed, 4 h later, by TR-gelatin. Two hours after TR-gelatin administration, mice were perfused with phosphate buffered saline (PBS) to eliminate the circulating fluorescent probe from blood vessels, including hepatic sinusoids, thus allowing analysis of the TR fluorescence exclusively bound or internalized by liver cells (Figure 7a). Immunofluorescence analysis of liver sections qualitatively showed a clear co-localization of CD206 and Texas Red (TR) fluorescence in mice treated with TR-gelatin only (Figures 7b and S15a). Conversely, no TR-gelatin could be visualized in liver sections of mice pre-treated with S_187_. In these samples, Oregon Green fluorescence from the S_187_ glycopolymer colocalized with CD206. While an in-depth investigation on the relative uptake of TR-gelatin by the different CD206-expressing hepatic cells is beyond the scope of the present study, we observed a significant co-localization of S_187_ with the vascular marker CD31, indicating that liver endothelial sinusoidal cells were major contributors to the uptake of the S_187_ glycopolymer (Figures S15b and S16). On the other hand, we did not observe co-localization of G_187_ with CD206 or CD31, with this polymer being mainly associated with hepatocytes in the liver parenchyma, thus explaining the higher TR-gelatin uptake by CD206 and CD31 positive cells upon animal pre-treatment with control galactose glycopolymer G_187_ than SO_4_-3-Gal glycopolymer blocker S_187_.

To obtain a quantitative comparison of the effect of the two glycopolymers on gelatin uptake, we performed fluorescence spectroscopy on liver homogenates. Data showed that pre-treatment with S_187_ caused a 70% decrease in liver TR-gelatin uptake, while administration of control polymer G187 did not affect gelatin uptake (Figure 7c; Table S3). As expected, plasma levels of TR-gelatin and Oregon Green-tagged glycopolymers were comparable in all animals treated with S_187_ and G_187_ (Figure S18), confirming that in these experiments, the same doses of TR-gelatin and Oregon Green-tagged glycopolymers were consistently administered to all mice. Taken together, these experiments show successful CD206 blockade by SO_4_-3-Gal glycopolymers in vivo, confirming the results obtained in our in vitro experiments.

## Conclusions

To our knowledge, our study represents the first example of effective CD206 inhibitors and provides an ab initio understanding of the molecular basis of a previously unknown mechanism of CD206 inhibition. Accordingly, we present here a new class of CD206 blockers based of glycopolymers bearing SO_4_-3-Gal sugar recognition elements designed to bind the CR ectodomain of CD206 and provide an initial structure–activity relationship for these multivalent ligands. This work shows that analogous mannosylated ligands targeting CTLD ectodomains of CD206 undergo a different intracellular trafficking compared to their SO_4_-3-Gal analogues, indicating that the nature of the multivalent sugar–ectodomain interactions ultimately dictates both the intracellular fate and the ability of these multivalent glycans to act as effective CD206 blockers. Data also showed that CD206 blockade following a single administration is transitory and is reproduced in vivo. At the same time, this study also provides important information on the chemistry of CD206 ligand binding and more specifically on how this is affected by ligand valency and intracellular pH. Thus, this study covers all aspects of the development of CD206 receptor blockers, from design and synthesis of the required polymeric multivalent ligands to investigation of their mechanisms of action and concept validation both in vitro and in vivo.

The potential implications of this work are manyfold. From a chemical perspective, non-covalent recognition of glycans to carbohydrate-binding proteins, lectins, typically requires multivalency, and several synthetic carbohydrate ligands have been used to intercept and dissect these processes. For example, the Ernst group recently described novel glycopolymer, against the DC-SIGN-mediated dissemination of SARS-CoV-2, based DC-SIGN antagonists in vitro in the context of SARS-CoV-2 infections,^76^ while Hartmann, Schelhaas, and co-workers designed highly sulfated synthetic glycopolymers and showed that they can act as effective broad-spectrum antiviral agents.^77^ Recently, glycopolymer probes have been used to elucidate how integrins can promote development of glioblastoma^42^ and how the molecular structure of sugar antigens affects cellular routing.^45^ However, thus far, use of such synthetic probes in vivo has been relatively limited because many lectins bind to the same glycan motifs; hence, selectivity can be challenging. The novel glycopolymer CD206 blockers presented in this work possess relatively uncommon SO_4_-3-Gal sugar functionalities which, though reported to bind relatively weakly to P-selectin,^66^ appear to be specific for the CR domain of CD206, and thus may be utilized both to dissect CD206-binding glycan function in vitro and to modulate its activity in vivo in the context of immunomodulation or prevention of pathogen infection.

From a therapeutic perspective, synthetic glycans share many of the features of macromolecular targeted therapeutics currently utilized in clinical settings, such as monoclonal antibodies, in that they can be used to selectively target selected protein receptors. However, multivalent synthetic glycans possess the advantage that their structural features—i.e., molecular topology and number and orientation of recognition elements—can be easily altered,^38^ and our data show that for CD206 this can be used to modulate the avidity of ligand–receptor binding. An additional advantage is that the in vivo stability profiles of multivalent synthetic glycans can potentially be tuned, which would be important for use in target tissues like the eye or brain where long-term stability toward biodegradation may be required.^78^ Despite its many roles at the interface between innate and adaptive immunity to maintain homeostasis and detect infections, CD206 is also utilized by several viral pathogens—e.g., HBV, HIV-1, and dengue virus—as a route of entry to initiate infection of host cells and has been associated with the development of diseases such as kidney crescentic glomerulonephritis, chronic obstructive pulmonary disease, and many cancers. Thus, within these clinical contexts, CD206 blockade would provide therapeutic benefits if effective inhibition strategies could be identified. Knowledge of the mechanism of CD206 inhibition will provide the basis for second generation chemical blockers with optimized molecular topology and valency. Future studies on the roles of CD206 inhibitors in specific viral and inflammatory diseases will be key to identify the clinical settings where the mechanism of CD206 blockade presented in this work can be exploited therapeutically, alone or as a part of specifically designed multidrug therapies.

## Supporting Information Available

Experimental details for both syntheses of glycopolymers and intermediates, cell, and in vivo work (PDF)

## Notes

Additional data related to this paper are available from the authors upon request and on the University of Nottingham Research Data Management Repository (https://rdmc.nottingham.ac.uk/).
