# Supplementary material for: Sulfation at Glycopolymer Side Chains Switches Activity at the Macrophage Mannose Receptor (CD206) In Vitro and In Vivo
Source: J Am Chem Soc. 2022 Dec 6;144(50):23134–47. doi: 10.1021/jacs.2c10757 (PMC9782796; doi:10.1021/jacs.2c10757)
Supplement: Supplementary file 1 — ja2c10757_si_001.pdf [file ja2c10757_si_001.pdf]

Supporting Information

Sulfation at glycopolymer side-chains switches activity at macrophage mannose receptor (CD206) in vitro and in vivo.

Francesca Mastrotto,[a,b,c] Marco Pirazzini,[d] Samuele Negro,[d] Alan Salama,[e] Luisa Martinez-Pomares,[b,*] and Giuseppe Mantovani [a,*]

[a]School of Pharmacy, University of Nottingham, Nottingham, NG7 2RD, UK, [b]School of Life Sciences, University of Nottingham, Nottingham, NG7 2RD, UK, [c]Department of Pharmaceutical and Pharmacological Sciences, University of Padova, via F. Marzolo 5, 35131, Padova, Italy, [d]Department of Biomedical Sciences, University of Padova, Via Ugo Bassi 58/B, 35131 Padova, Italy, [e]Department of Renal Medicine, University College London, NW3 2PF, UK.

1 Materials & Methods

1.1. Materials

6-azido-2,4,5,7,7'-pentafluorofluorescein (Oregon Green azide),[1] 2'-azidoethyl-O-D-galactopyranoside and 2'-azidoethyl-O-D-mannopyranoside[2] were synthesised as previously described. Triethylamine, 4-fluororesorcinol, tetrafluorophtalic anhydride, 3-dimethyl aminophenol, sodium nitrate, 1-naphtylamine, sodium iodide, 5-dimethylamino-2-nitrosophenol hydrochloride were purchased from Alfa Aesar or Acros Organics. NaN_3, methansulfonic acid, amberlite IR-120, Dowex® 50WX8 Na^+ form 200-400 mesh, 2-bromoethanol, D-galactose, D-mannose, dibutyltin oxide, phenylboronic acid, trimethylamine sulfur trioxide complex, Cu(II)Br, bipyridine, gelatin from porcine skin, Triton X-100, sodium deoxycholate, DNase, sulforhodamine 101 acid chloride (Texas Red), mannan from *Saccharomyces cerevisiae* (CAS 9036-88-8), chondroitin sulfate A sodium salt from bovine trachea (CAS 39455-18-0), DMEM/F12, Tris-HCl, phosphate buffer saline, trypsin-EDTA solution, penicillin/streptomycin and glutamine solutions, p-nitrophenyl phosphate substrate, non-enzymatic cell dissociation buffer, anti-EEA1 and anti-LAMP1 antibodies, HOECHST 33342 stain solution, RPMI 1640 medium, Fetal Bovine Serum (FBS), sterile-filtered (low endotoxin < 1 EU mL^{-1}) water were supplied by Sigma-Aldrich. Mouse M-CSF was purchased from R&D Systems (Inc., Minneapolis USA). All solvents and salts for buffer were obtained from Fisher Scientific or VWR and were of analytical grade or superior. Spectra/Por 8 kDa MWCO and SnakeSkin 3.5 kDa MWCO dialysis tubing were obtained from VWR International and Thermo Fisher Scientific, respectively. Dulbecco's modified Eagle's medium/Ham's F-12 nutrients and opti-MEM® reduced serum medium without phenol red were supplied by GIBCO

(Invitrogen, Carlsbad, CA, USA). Cytotoxicity Detection Kit (LDH) and cOmplete EDTA-free Protease Inhibitor Tablets were purchased from Roche Diagnostics. Pierce BCA Protein Assay Kit, Hybond™-C Extra – Amersham Bioscence Nitrocellulose membrane and GE Healthcare Amersham™ Hyperfilm™, PageRuler™ Plus Prestained Protein Ladder, ECL solution and GE Healthcare CM-5 sensor chip were supplied by Thermo Fisher Scientific. Heat-inactivated goat serum was provided by MP Biomedical. Alexa Fluor® 647 goat anti-rabbit (H+L) and Alexa Fluor® 647 goat anti-rat (H+L) were obtained from Molecular Probes (Breda, the Netherlands). DAKO fluorescent mounting media was supplied by DAKO, Agilent (North America, Inc. Ca 93013 USA). Alexa Fluor® 647 anti-mouse CD206 Antibody, Alexa Fluor® 647 Rat IgG2a, k Isotype Ctrl and Alexa Fluor® 647-conjugate goat anti-Rat IgG were purchased from Biolegend (San Diego, CA, USA). HRP-conjugated goat-anti-Rat IgG was obtained from Source Bioscence. Goat Anti-human IgG (Fcγ specific) alkaline phosphatase conjugate was supplied by Jackson ImmunoResearch. MR5D3 and IgG2a,[3] soluble form of CD206 (sCD206),[4] and the hybridomas 2.4G2 (anti-CD32)[5] were produced as described previously.

15-2 CD206 monoclonal antibody used for tissue immunostaining was obtained from Santa Cruz Biotechnology, Zoletil® was from Virbac (Carros, France) and Xilor was from Laboratorios Calier S.A. (Barcelona, Spain), HOECHST 33342 for nuclei staining and anti-CD31 monoclonal antibody (P8590) were obtained from Sigma-Aldrich. Alexa Fluor® 488 goat anti mouse secondary antibody was obtained from Thermo Fisher Scientific, PolyFreeze (Tissue Freezing Medium) was obtained from Polysciences Europe GmbH.

1.2 Analysis

^{1}H and ^{13}C{^{1}H} NMR spectra of low molecular weight intermediates and ^{1}H NMR of glycopolymers were recorded at room temperature on a 400 MHz (Bruker DPX400 Ultrashield) using deuterated solvents ($CDCl_3$, D_2O or DMSO-d_6). ^{13}C{^{1}H} NMR spectra of glycopolymers were recorded on a Bruker AV(III) 500 NMR Spectrometer with Carbon-Proton Cryoprobe facilities. Quantitative ^{13}C NMR spectra of Oregon Green-labelled galactose-3-O-sulfated glycopolymers were acquired at 298 K, with an inverse-gated decoupling pulse sequence, relaxation delay of 50 s (^{13}C T1 measurements were carried out prior to running ^{13}C quantitative NMR analysis on the samples). All chemical shifts are reported in parts per million (ppm). Mass spectra (TOF-ESI) were recorded on a Waters 2795 separation module/micromass LCT platform. FT-IR spectra were recorded with an Attenuated Total Reflection spectrophotometer (Agilent Technologies Cary 630 FTIR) equipped with a diamond single reflection ATR unit. Spectra were acquired with a resolution of 4 cm^{-1}, in the range 4000-650 cm^{-1} by recording 32 interferograms.

Average molecular weights and dispersities (Đ) of poly(propargyl methacrylate)s were determined by size-exclusion chromatography (SEC) performed on a Polymer Laboratories GPC 50 system (Polymer Laboratories) equipped with Refractive Index detector and two PLgel

Mixed-D (5 µm bead, 7.8-300 mm) columns with a matching guard (7.8-50 mm), using chloroform as the mobile phase, at a flow rate of 1 mL min^{-1}. Calibration was performed using narrow molar mass range polystyrene standards (Polymer Labs) in the 0.13–210 kDa molecular weight range. DMF SEC of glycopolymers was carried out on a pair of Agilent PLgel 5 µm Mixed D columns (7.5 x 300 mm, 5 µm bead size, Polymer Labs UK), eluting with DMF + 0.1 % w/w LiBr at flow rate of 1 mL min^{-1}. Aqueous SEC was carried out with a Shimadzu UPLC system fitted with a differential refractive index detector. The mobile phase was DPBS, pH 7.4 at 25ºC and a flow rate of 1.0 mL·min^{-1}. The instrument was fitted with a Polymer Labs aquagel-OH guard column (50 x 7.5 mm, 8 µm) followed by three PL aquagel-OH columns (30, 40 and 50; 300 x 7.5 mm, 8 µm). Column calibration was achieved using PEG/PEO narrow standards with Mp in the 0.200 – 130 kDa range. Molecular weights and dispersity values were calculated using Shimadzu LabSolutions software with SEC.

1.3 Synthesis of glycopolymer CD206 ligands

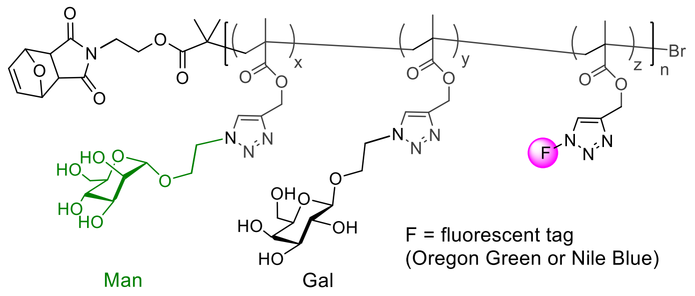

glycopolymer	alt. name	n	x	y	z
$(Gal_{100\%})_{32}$	G_{32}	32	0	0.99	0.01
$(M_{33\%})_{32}$	-	32	0.33	0.66	0.01
$(M_{66\%})_{32}$	-	32	0.66	0.33	0.01
$(M_{100\%})_{32}$	M_{32}	32	0.99	0	0.01
$(M_{100\%})_{187}$	M_{187}	187	0.99	0	0.01

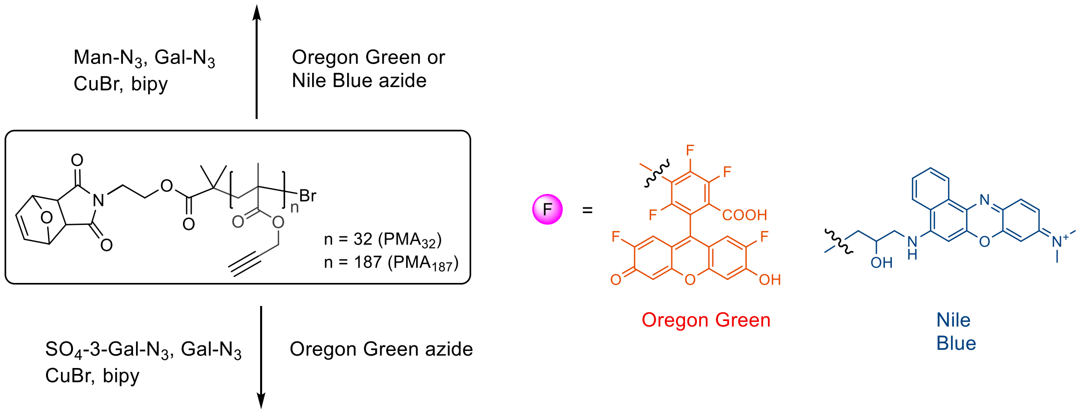

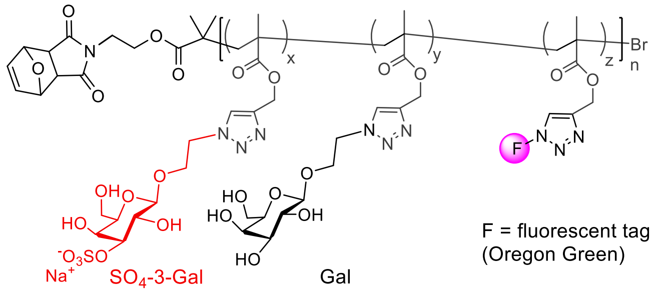

glycopolymer	alt. name	n	x	y	z
$(Gal_{100\%})_{32}$	G_{32}	32	0	0.99	0.01
$(S_{33\%})_{32}$	-	32	0.33	0.66	0.01
$(S_{66\%})_{32}$	-	32	0.66	0.33	0.01
$(S_{100\%})_{32}$	S_{32}	32	0.99	0	0.01
$(S_{100\%})_{187}$	S_{187}	187	0.99	0	0.01

Scheme S1. Synthesis of fluorescent glycopolymer CD206 ligands. All glycopolymers were fluorescently tagged with Oregon Green ($\lambda_{ex,max}$= 495 nm, $\lambda_{em,max}$= 535 nm). In addition, one batch of $(M_{100\%})_{32}$ (M_{32}) was also tagged with Nile Blue ($\lambda_{ex,max}$= 650 nm, $\lambda_{em,max}$= 677 nm) (M_{32}-NB) and used as fluorescent ligand probe as an alternative to Texas Red-tagged gelatin for subsequent uptake inhibition studies.

1.3.1 2'-azidoethyl-*O*-*α*-D-mannopyranoside ($Man-N_3$) and 2'-azidoethyl-*O*-*α*-D-galactopyranoside ($Gal-N_3$)

2'-azidoethyl-*O*-*α*-D-mannopyranoside ($Man-N_3$) and 2'-azidoethyl-*O*-*β*-D-galactopyranoside ($Gal-N_3$) were synthesised as described previously.[2]

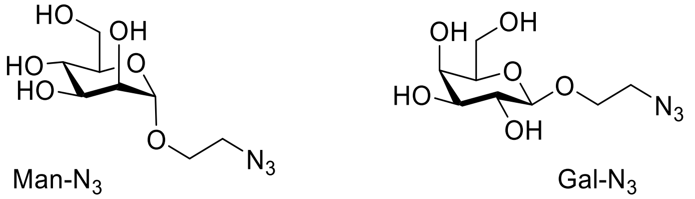

Figure S1. Chemical structure of 2'-azidoethyl-*O*-α-D-mannopyranoside ($Man-N_3$) and 2'-azidoethyl-*O*-β-D-galactopyranoside ($Gal-N_3$).

2'-azidoethyl-O-α-D-mannopyranoside ($Man-N_3$). ^{1}H NMR (400 MHz, D_2O, δ): 3.52-3.67 (m, 2H, CH_2N_3); 3.71-3.78 (m, 2H, $CH_2CH_2N_3$); 3.78-3.88 (m, 2H, CH_2OH); 3.89-4.08 (m, 4H, 4 CH); 5.00 (d, *J* = 1.7 Hz, 1H, $CH_{anomeric}$). $^{13}C\{^{1}H\}$ NMR (100.59 MHz, D_2O, δ): 50.3 (1C, CH_2Br); 61 (1C, CH_2OH); 66.45 (1C, *C*H); 66.78 (1C, OCH_2CH_2Br); 70.0 (1C, *C*H); 70.5 (1C, *C*H); 72.95 (1C, *C*H); 99.89 (1C, $CH_{anomeric}$). HRMS (ESI) m/z: $[M + H]^+$ calcd for $C_{21}H_{38}N_4O_6S$, 475.2591; found, 475.2593.ESI-TOF *m/z*: $[M + Na]^+$ calcd for $C_8H_{15}N_3O_6Na$ 272.0853; found 272.0936. FT-IR: v = 3403, 3271, 2932, 2111, 1637, 1617, 1339, 1261, 1139, 1077, 1052, 971, 886, 812, 606 cm^{-1}.

2'-azidoethyl-O-β-D-galactopyranoside ($Gal-N_3$). ^{1}H NMR (400 MHz, D_2O, δ): 3.49-3.55 (m, 1H, CH); 3.56 (t, *J* = 4.9 Hz, 2H, CH_2N_3); 3.63-3.89 (m, 5H, 1 CH+CH_2OH+$CH_2CH_2N_3$); 3.93 (d, *J* = 3.1 Hz, 1H, CH) 4.07 (dt, *J* = 11.1, 4.7 Hz, 1H, $CHCH_2OH$); 4.44 (d, *J* = 7.8 Hz, 1H, $CH_{anomeric}$). $^{13}C\{^{1}H\}$ NMR (100.59 MHz, D_2O, δ): 50.6 (1C, CH_2Br); 61.0 (1C, CH_2OH); 68.4 (1C, *C*H); 68.6 (1C, OCH_2CH_2Br); 70.7 (1C, *C*H); 72.7 (1C, *C*H); 75.2 (1C, *C*H); 102.9 (1C, $CH_{anomeric}$). ESI-TOF *m/z*: $[M + Na]^+$ calcd for $C_8H_{15}N_3O_6Na$ 272.0853; found 272.1029. FT-IR: v 3412, 2934, 2112, 1637, 1400, 1343, 1290, 1148, 1071, 976, 864, 781, 621 cm^{-1}.

1.3.2. Synthesis of 3-*O*-sulfo-2'-azidoethyl-*O*-*β*-D-galactopyranoside (SO_4-3-Gal-N_3)

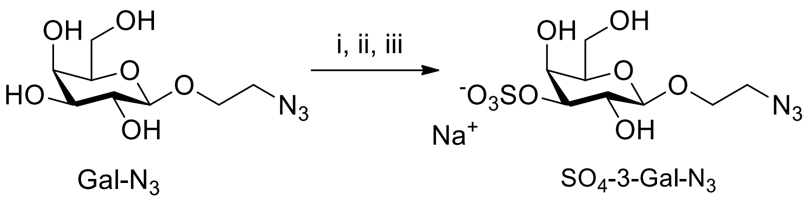

Scheme S2. Synthesis of 3-*O*-sulfo-2'-azidoethyl-*O*-β-D-galactopyranoside. *Reagents and conditions:* (i) phenylboronic acid, MeOH/EtOAc 2:1, reflux, 2 h; (ii) dibutyltin oxide, MeOH/EtOAc 2:1, reflux 2 h; (iii) trimethylamine sulfur trioxide complex, DMF, 15 h, room temperature.

3-*O*-sulfo-galactose was synthesized following the method reported by Kiessling and co-workers,[6, 7] with some modifications.

To a solution of 2'-azidoethyl-*O*-β-D-galactopyranoside (1.00 g, 4.02 mmol) in MeOH (10 mL), EtOAc (5 mL) and solid phenylboronic acid (490 mg, 4.02 mmol) were added. A Dean-Stark apparatus, pre-filled with EtOAc, was set up and the reaction mixture was stirred under reflux for 2 h. The reaction solution was then concentrated to ca. 5 mL, a fresh portion of MeOH/EtOAc (10 mL) and solid dibutyltin oxide (1.26 g, 4.42 mmol) were added, and the reaction mixture was refluxed using the Dean-Stark apparatus for further 2 h. The solvent was then removed under reduced pressure, and the resulting residue was dissolved in anhydrous DMF (5 mL) and added of trimethylamine sulfur trioxide (700 mg, 5.03 mmol). After stirring overnight at room temperature, MeOH (5 mL) was added to stop the reaction, the solvent removed under reduced pressure, and the residue added of a second portion of MeOH (5 mL) and stirred. After 10 min, the crude product was precipitated in Et_2O, redissolved in methanol and precipitated again in Et_2O to remove any residual traces of DMF. The resulting precipitate was added of MeOH (5 mL), diluted with water (10 mL) and extracted with Et_2O (3 x 10 mL). The aqueous layer was then added of anionic exchange resin (Dowex Na^+, previously extensively washed with methanol). The mixture was stirred for 15 min, then the resin was removed by filtration. The aqueous solution was freeze-dried, and the resulting residue was purified by flash chromatography (silica gel 60, 35-70 μm, gradient elution 9:1 to 7:3 EtOAc/MeOH), to give 3-*O*-sulfo-2'-azidoethyl-*O*-β-D-galactopyranoside sodium salt (1.17 g, 3.33 mmol, 83%) as a white solid.

^{1}H NMR (400 MHz, D_2O, δ): 3.6 (t, *J* = 5.0 Hz, 2H, C$H_2$$N_3$); 3.72 (dd, *J* = 9.7, 7.9 Hz, 1H, C*H*); 3.83-3.75 (m, 2H, CH_2C$H_2$$N_3$); 3.86-4.18 (m, 2H, C$H_2$OH); 4.31-4.41 (m, 2H, 2 CH); 4.36 (m, 1H, CH); 4.6 (d, *J* = 7.9 Hz, 1H, CH). ^{13}C{^{1}H} NMR (100.59 MHz, D_2O, δ): 50.6 (1C, *C*$H_2$$N_3$); 66.8 (1C, *C*$H_2$OH); 66.9 (1C, *C*H); 68.5 (1C, O*C*H_2CH_2Br); 68.8 (1C, *C*H); 74.8 (1C, *C*H); 80.3

(1C, *C*H); 102.6 (1C, *C*$H_{anomeric}$). ESI-TOF *m/z*: $[M - H]^-$ calcd for $C_8H_{14}N_3O_9S^-$ 328.0451; found 328.0246. FT-IR: ν 3434, 2118, 1635, 1235, 1151, 1063, 990 cm^{-1}.

1.3.3 Synthesis of *N*-(5-((3-azido-2-hydroxypropyl)amino)-9H-benzo[a]phenoxazin-9-ylidene)-*N*-methylmethanaminium (Nile Blue azide)

N-(5-((3-azido-2-hydroxypropyl)amino)-9H-benzo[a]phenoxazin-9-ylidene)-*N*-methylmethanaminium (Nile Blue azide) was synthesised by adapting a protocol previously described by Kele *et al.* for the synthesis of other stucturally similar Nile Blue azides.[8]

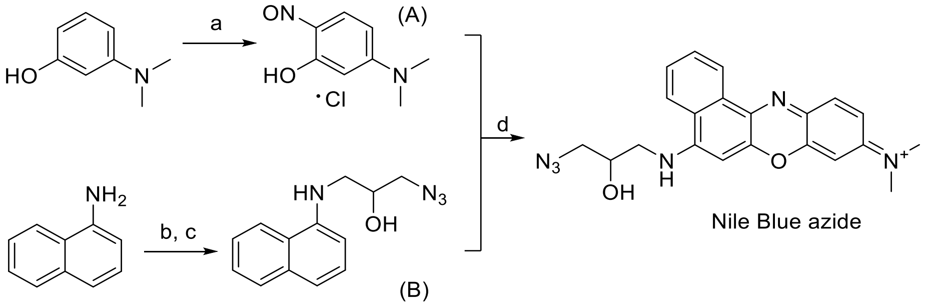

Scheme S3. Synthesis of Nile blue azide. *Reagents and conditions*: a) $NaNO_2$, HCl in EtOH/water; b) 1-bromo-3-chloro-2-propanol in EtOH, reflux, 12 h; c) NaI, NaN_3, DMF, room temperature, 24 h; d) ethanol, HCl, reflux, 9 h.

5-(dimethylamino)-2-nitrosophenol hydrochloride (A). An ice cold solution of 3-dimethyl aminophenol (1.5 g, 11 mmol) in EtOH (15 mL) was added of concentrated aqueous HCl (0.75 mL). A solution of sodium nitrite (840 mg, 12.2 mmol) in water (1.5 mL) was added dropwise over 20 min to the dimethyl aminophenol acidic solution. At complete reaction, as assessed by TLC, the organic solvent was removed under reduced pressure, the crude product 5-(dimethylamino)-2-nitrosophenol hydrochloride (A) was freeze-dried to remove residual traces of water, and used without further purification for the next step (1.80 g, 10.8 mmol, 97.7%).

^1H NMR (400 MHz, $CDCl_3$, δ): 3.25 (s, 6H, N(C*H_3*)$_2$); 5.73 (d, *J* = 2.6 Hz, 1H, Ar); 6.62 (dd, *J* = 9.9, 2.6 Hz, 1H, Ar); 7.45 (d, *J* = 9.9 Hz, 1H, Ar). ESI-TOF *m/z*: $[M + H]^+$ calcd for $C_8H_{11}N_2O_2$ 167.0815; found 167.8061. FT-IR: ν 3434, 2055, 1655, 566 cm^{-1}.

1-azido-3-(naphthalen-1-ylamino)propan-2-ol (B). 1-bromo-3-chloro-2-propanol (2.5 g, 14 mmol) was added to a solution of 1-naphtylamine (1.87 g, 13.1 mmol) in ethanol (4 mL) and the reaction mixture was heated at reflux under stirring for 12 h. The reaction was monitored by TLC. Then the organic solvent was removed under reduced pressure and the crude product was purified by flash chromatography (silica gel 60, 35-70 µm, petroleum ether/CH_2Cl_2 4:6 to

100% CH_2Cl_2) to give 1-chloro-3-(naphthalen-1-ylamino)propan-2-ol (500 mg, 2.07 mmol). The isolated intermediate was reacted with sodium iodide (31.7 mg, 0.212 mmol) and sodium azide (165 mg, 2.54 mmol) in DMF (2.5 mL) at 80°C. After 24 h the reaction solution was diluted with EtOAc and washed 3 times with water. The organic layer was dried over $MgSO_4$ and evaporated to dryness to give 1-azido-3-(naphthalen-1-ylamino)-2-propanol (B) (450 mg, 1.85 mmol, 89.3%).

^{1}H NMR (400 MHz, $(CD_3)_2CO$, δ): 3.46 (m, 2H, CH_2N_3); 3.55 (m, 2H, $NHCH_2$); 4.33 (m, 1H, CH-OH); 4.93 (bs, 1H, OH); 5.56 (bs, 1H, NH); 6.72 (d, *J* = 7.45 Hz, 1H, Ar CH); 7.29 (d, *J* = 8.2 Hz, 1H, Ar); 7.37-7.43 (m, 1H, Ar); 7.43-7.56 (m, 2H, Ar); 7.82-7.88 (m, 1H, Ar); 8.07-8.15 (m, 1H, Ar). ^{13}C{^{1}H} NMR (100.59 MHz, $(CD_3)_2CO$, δ): 48.1 (1 C, CH_2NH); 55.54 (1 C, CH_2N_3); 69.67 (1 C, CHOH); 104.69 (1 C, Ar); 117.55 (1 C, Ar); 121.43 (1 C, Ar); 124.47 (1 C, Ar); 125.09 (1 C, Ar); 126.35 (1 C, Ar); 127.44 (1 C, Ar); 129.05 (1 C, Ar); 135.29 (1 C, Ar); 144.55 (1 C, Ar). ESI-TOF *m/z*: $[M + H]^+$ calcd for $C_{13}H_{15}N_4O$ 243.1240; found 243.5551. FT-IR: ν 3434, 2101, 1581, 1526, 1408, 1281, 770 cm^{-1}.

N-(5-((3-azido-2-hydroxypropyl)amino)-9H-benzo[a]phenoxazin-9-ylidene)-N-methylmethanaminium (Nile Blue azide). To an ice cooled solution of 5-dimethylamino-2-nitrosophenol hydrochloride (330 mg, 1.63 mmol) in 8 mL of ethanol 1-azido-3-(naphthalen-1-ylamino)-2-propanol (400 mg, 1.66 mmol) and concentrated hydrochloride acid (0.17 mL) were added. The reaction was maintained under reflux for 9 h and monitored by TLC (CH_2Cl_2/MeOH 9.5:0.5). The solvent was then removed under reduced pressure and the crude product purified by flash chromatography (silica gel 60, 35-70 µm, CH_2Cl_2/MeOH 9.5:0.5). *N*-(5-((3-azido-2-hydroxypropyl)amino)-9H-benzo[a]henoxazin-9-ylidene)-*N*-methylmethanaminium (Nile Blue azide) was obtained as a blue solid (500 mg, 1.29 mmol, 77.4%).

^{1}H NMR (400 MHz, MeOD, δ): 3.08 (s, 6H, $N(CH_3)_2$); 3.24-3.29 (m, 2H, CH_2N_3); 3.41-3.57 (m, 2H, $NHCH_2$) 3.66-3.67 (m, 2H*,* C*H*OH + CHO*H*); 4.21 (m, 1H, NH); 6.34 (s, 1H, Ar); 6.69 (s, 1H, Ar); 6.85 (dd, *J* = 9.3, 1.9 Hz, 1H, Ar); 7.28 (d, *J* = 8.6 Hz, 1H, OH); 7.36 (dd, *J* = 9.4, 2.7 Hz, 1H, Ar); 7.59 (t, *J* = 7.4 Hz, 1H, Ar); 7.66 (t, *J* = 7.6 Hz, 1H, Ar), 8.11 (d, *J* = 8.1 Hz, 1H, 1H, Ar); 8.36 (d, *J* = 8.0 Hz, 1H, Ar). ^{13}C{^{1}H} NMR (100.59 MHz, $(CD_3)_2CO$, δ): 41.15 (2C, N*C*H_3); 49.85 (1C, NH*C*H_2); 55.72 (1C, *C*H_2N_3); 70.34 (1C, *C*HOH); 95.00 (1C, Ar); 96.87 (1C, Ar); 116.49 (1C, Ar); 123.87(1C, Ar); 124.30 (1C, Ar); 125.30 (1C, Ar); 130.77 (1C, Ar); 131.31 (1C, Ar); 131.96 (1C, Ar); 132.82 (1C, Ar); 133.42 (1C, Ar); 134.27 (1C, Ar); 148.53 (1C, Ar); 152.27 (1C, Ar); 156.87 (1C, Ar); 159.51 (1C, Ar). ESI-TOF *m/z*: $[M]^+$ calcd for $C_{21}H_{21}N_6O_2^+$ 389.1721, found 389.0630. FT-IR: ν 3329, 2094, 1640, 1045, 1024, 989 cm^{-1}.

1.3.4 Synthesis of poly(propargyl methacrylate)s clickable scaffolds $(PMA)_{32}$ and $(PMA)_{187}$

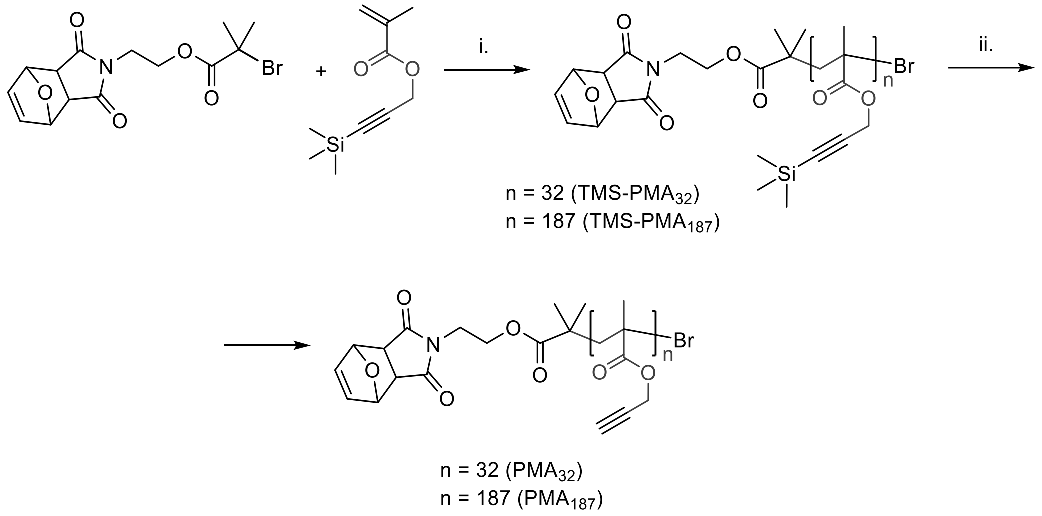

Scheme S4. Synthesis of poly(propargyl methacrylate) scaffolds. *Reagent and conditions*: *i*) *N*-(ethyl)-2-pyridylmethanimine, Cu(I)Br, toluene, 30°C; *ii*) acetic acid, THF, TBAF, room temperature, 18 h.

Poly(propargyl methacrylate) clickable scaffolds were prepared as described by Geng *et al.*[2] using the conditions shown below:

(propargyl methacrylate)$_{32}$ (PMA$_{32}$). Trimethylsilyl-propargylmethacrylate (2.0 g, 10 mmol), *N*-(ethyl)-2-pyridylmethanimine ligand (4.5 mg, 0.34 mmol), 2-(1,3-dioxo-1,3,3a,4,7,7a-hexahydro-2H-4,7-epoxyisoindol-2-yl)ethyl 2-bromo-2-methylpropanoate ATRP initiator (30 mg, 0.084 mmol). Solvent: anhydrous toluene (6 mL). T = 30°C, final monomer conversion: 73%. Removal of TMS protection groups was carried out using TBAF and acetic acid, as described by Geng *et al.*[2] to give (propargyl methacrylate)$_{32}$ (PMA$_{32}$) as a white solid (1.10 g, 8.2 mmol of alkyne functionalities). $M_{n,NMR}$ = 4.33 kDa; $M_{n,SEC}$ ($CHCl_3$) =4.27 kDa, Đ = 1.32.

Degree of polymerization (DP) and $M_{n,NMR}$ were estimated by comparing the signals of the polymer chain-end at 5.3 (C*H*O) and 6.6 (CH_{vinyl}) ppm, and those of the propargyl ester repeating units at 4.6 (CH_2O) and 2.5 (C≡CH) ppm.

Synthesis of (propargyl methacrylate)$_{187}$ (PMA$_{187}$).
[Trimethylsilyl-propargylmethacrylate]$_0$:[initiator]$_0$:[*N*-(ethyl)-2-pyridylmethanimine ligand]$_0$:[CuBr]$_0$=200:1:4:2; [monomer]$_0$=2.12 M, toluene: 6 mL, T=40°C, final monomer conversion: 44%.

(propargyl methacrylate)$_{187}$ (PMA$_{187}$) was isolated as an off-white solid (1.7 g, 13.7 mmol of alkyne functionalities). $M_{n,NMR}$ = 23.5 kDa; $M_{n,SEC}$ ($CHCl_3$) = 9.76 kDa, Đ = 1.87.

Degree of polymerization (DP) and $M_{n,NMR}$ were estimated by comparing the signals of the polymer chain-end at 5.3 (C*H*O) and 6.6 (CH_{vinyl}) ppm, and those of the propargyl ester repeating units at 4.6 (CH_2O) and 2.5 (C≡CH) ppm.

1.3.6 Synthesis of Oregon Green-labelled glycopolymers

Glycopolymers were prepared by clicking Oregon Green and sugar azides to appropriate poly(propargyl methacrylate) polymeric precursors by CuAAC.[2]

As an example, the preparation of Oregon Green-tagged $(S_{100\%})_{32}$ (S_{32}) is described below.

A solution of poly(propargyl methacrylate) (DP 32, 300 mg, 2.42 mmol of clickable alkyne units), Oregon Green azide (11 mg, 0.024 mmol) and bipyridine (152 mg, 0.967 mmol) in DMF (15 mL) were degassed by nitrogen bubbling for 15 min. CuBr (I) (69.5 mg, 0.484 mmol) was then added to the reaction mixture under a positive flow of nitrogen, and the solution was bubbled with nitrogen for further 15 minutes. The deep purple solution was stirred at room temperature for 3 days. The reaction was monitored by SEC with visible (λ = 496 nm) and RI detection. A 5 mL aliquot was withdrawn, and to this 3-O-sulfo-2'-azidoethyl-O-galactopyranoside (6) (319 mg, 0.968 mmol) was added and the solution degassed for 15 min under nitrogen. A solution of sodium ascorbate (32 mg, 0.16 mmol) in water (100 µL) was added to the mixture with a deoxygenated syringe, and the reaction was stirred at room temperature for further 2 days. The glycopolymer was precipitated in THF, isolated by centrifugation, re-dissolved in water, transferred into a dialysis membrane (MWCO 3.5 kDa) and dialyzed in the dark against aqueous EDTA, then DI water for 3 days. The polymer aqueous solution was then freeze-dried to give $(S_{100\%})_{32}$ (S_{32}) as a pink-orange solid.

For the synthesis of glycopolymers with 66 or 33% of mannosylated or galactose 3-*O*-sulfate sugars, the procedure is analogous to that described above, except that after clicking of the Oregon Green azide, mannose and sulfated galactose azides were first added to the reaction mixture according to the required molar ratio (alkyne units/sugar 100:66 or 100:33) and stirred at room temperature. After 3 days an excess of galactose azide, to give a total (sugar azide)/alkyne units molar ratio of 1.5:1, was added and the mixture left to react for further 3 days, to react all the clickable units available. The synthetic protocol followed in this work diverges slightly from that reported previously by Haddleton and us,[9] in that the sugar and fluorescent azides were added sequentially over time, rather than used all together from the beginning of the click reaction. This was done because in our previous study we only utilized 2'-azidoethyl-*O*-galacto- and 2'-azidoethyl-*O*-mannopyranoside, which for this 'click' reaction one can assume they are so similar that their kinetics of reaction with poly(propargyl methacrylate) are almost identical. Hence a copolymer with a specific relative content of these

two sugar moieties could be prepared by simply mixing the two sugar azide precursors in the same molar ratio (co-clicking). In this work, however, 3-*O*-sulfo-2'-azidoethyl-*O*-galactopyranoside is larger than the corresponding Man and Gal azides, and is also negatively charged, hence the assumption that it would react with the same rate as the other azides may not be valid. Therefore, here functionalisation of clickable poly(propargyl methacrylate) was carried out by adding the required sugar azides in a sequential manner over time, as described above.

The synthesis of Nile blue-labelled polymer (M_{32}-Nile Blue) was performed under identical conditions, but with using Nile blue azide instead of Oregon Green azide.

Table S1. Characterization of glycopolymers synthesized in this study.

Code	alt. code	Man (M)[a]	SO_4-3-Gal (S)[a]	Gal (G)[a]	Fluor.[a),b)]	$M_{n,theor}$ (kDa)[c)]	$M_{n,SEC}$ (kDa)	Đ
$(G_{100\%})_{32}$	G_{32}	-	-	0.99	0.01	12.3	14.6[d)]	1.30[d9]
$(G_{100\%})_{187}$	G_{187}	-	-	0.99	0.01	70.2	29.2[d)]	1.54[d)]
$(M_{33\%})_{32}$		0.33	-	0.66	0.01	12.3	14.4[d)]	1.26[d)]
$(M_{66\%})_{32}$		0.66	-	0.33	0.01	12.3	14.0[d)]	1.26[d)]
$(M_{100\%})_{32}$	M_{32}	0.99	-	-	0.01	12.3	13.0[d)]	1.23[d)]
	$M_{32\text{-Nile Blue}}$	0.99	-	-	0.01	12.3	16.5[d)]	1.20[d)]
$(M_{100\%})_{187}$	M_{187}	0.99	-	-	0.01	70.2	28.9[d)]	1.47[d)]
$(S_{33\%})_{32}$		0.33	-	0.66	0.01	13.4	11.6[e)]	1.22[e)]
$(S_{66\%})_{32}$		0.66	-	0.33	0.01	14.5	12.8[e)]	1.20[e)]
$(S_{100\%})_{32}$	S_{32}	0.99	-	-	0.01	15.6	14.2[e)]	1.18[e)]
$(S_{100\%})_{187}$	S_{187}	0.99	-	-	0.01	89.3	26.0[e)]	1.36[e)]

[a)]Molar fraction of each repeating unit (e.g. 0.33 indicates that 33% of polymer repeating units contain that specific sugar molecule, 0.01 that 1% of repeating units are functionalised with a fluorophore (Fluor.), etc). Molar fractions are those expected based on the relative amount of reagents used in the 'click' reaction feed. [b)]All glycopolymers are fluorescently tagged with Oregon Green, except for $M_{32\text{-Nile Blue}}$, which was tagged with Nile Blue. [c)]Calculated by multiplying the degree of polymerisation (DP) of the relevant clickable poly(propargyl methacrylate) precursor (either 32 or 187) by the molecular mass of the clicked sugar repeating units, + the molecular weight of ATRP initiator used to prepare the clickable precursor: $M_{n,theor} = (DP \times MW_{sugar\ repeating\ units}) + MW_{ATRP\ initiator}$. [d)]Obtained from SEC analysis using DMF+0.1% LiBr as the mobile phase (PMMA standards). [e)]Obtained from SEC analysis using PBS as the mobile phase (PEG standards). For clarity, glycopolymers where all repeating units (100%) present the same monosaccharide repeating units – e.g. $(G_{100\%})_{32}$, $(M_{100\%})_{32}$, etc. – were renamed with a simpler alternative polymer code, as indicated in the **alt. code** column.

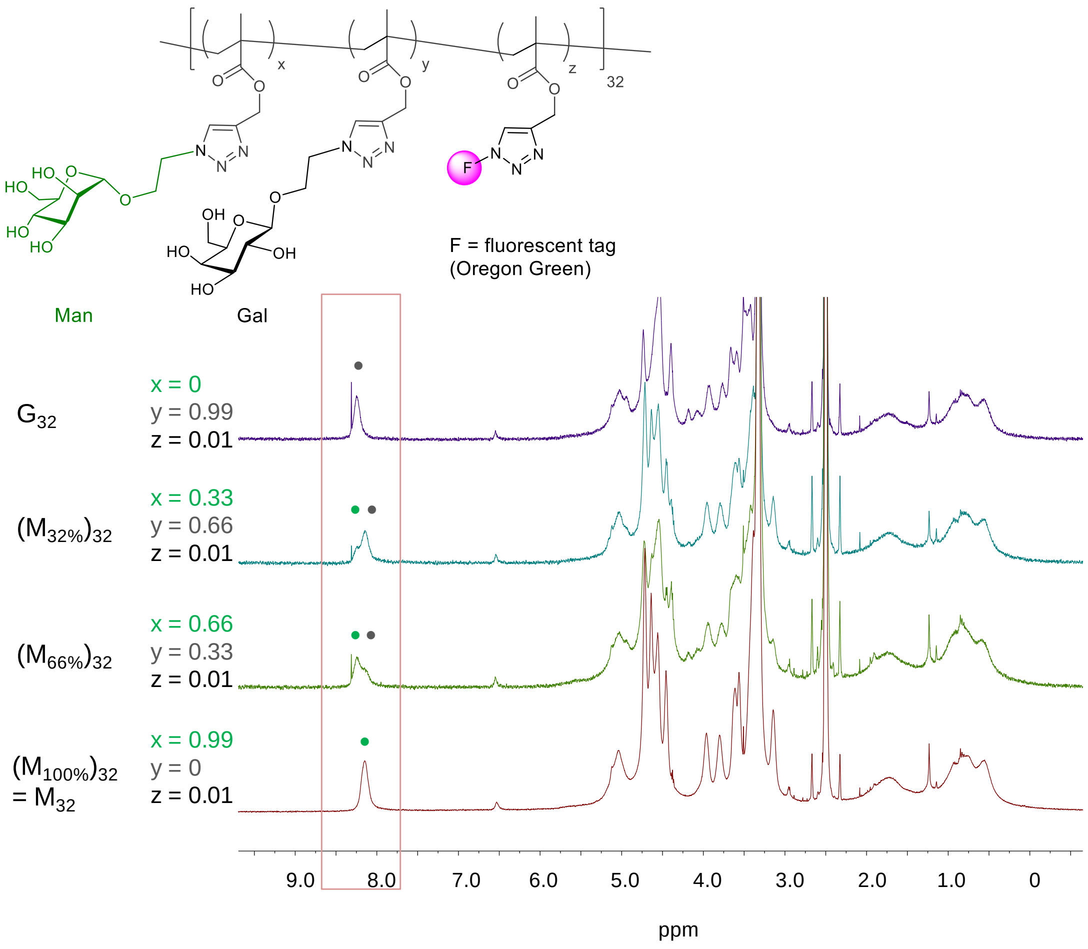

Figure S2. Overlay of ^{1}H NMR spectra of mannosylated glycopolymers (DP 32) with different mannose content, in DMSO-d_6. The 7.6-8.1 ppm region is highlighted to show how the triazole signal pattern changes at different galactose (●) : mannose (●) ratios.

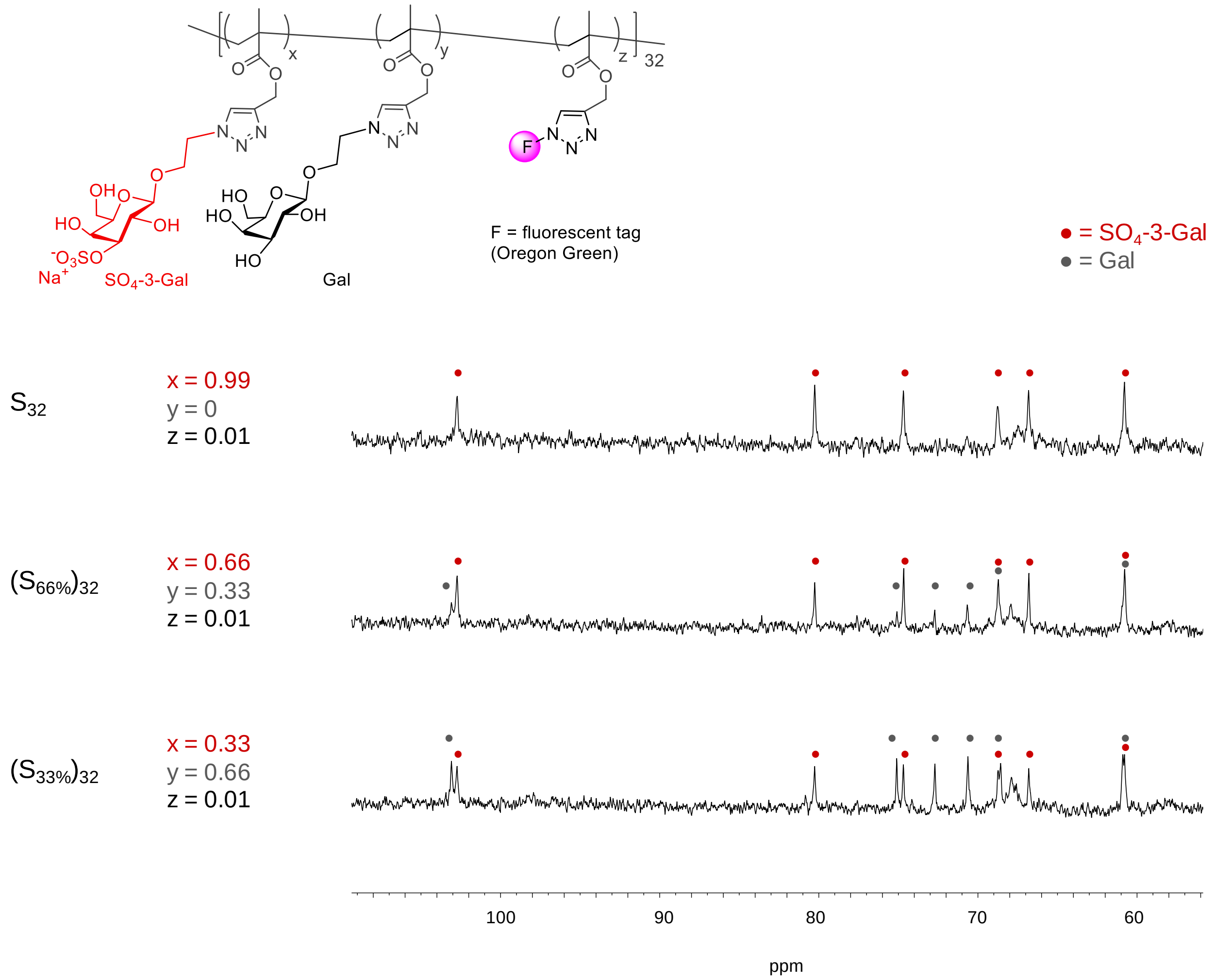

Figure S3. Overlay of ^{13}C NMR spectra of glycopolymers (DP 32) with different galactose-3-sulfate content, in DMSO-d_6. For this subfamily of CD206 ligands, ^{13}C NMR allows better characterization of glycopolymers with different galactose (●):SO_4-3-galactose (●) polymer ratios than ^1H NMR, due to the signal broadening observed in the ^1H NMR spectra as the galactose-3-sulfate content increases (Figures S33-35, and S38).

1.4 Synthesis of Texas Red-tagged gelatin (TR-Gelatin)

Texas Red-tagged gelatin was prepared following the procedure described by Hummert *et al.*[10]

A 10 mg mL^{-1} gelatin solution was prepared by dispersing 100 mg of porcine skin gelatin in 10 mL of 0.1 M sodium bicarbonate buffer, pH 9.0, at room temperature. The suspension was stirred gently for 3 h and then heated at 45°C until completely dissolved (12 h). A 25 mg mL^{-1} stock solution of sulforhodamine 101 acid chloride (Texas Red) was prepared by dissolving 2.5 mg of the fluorescent dye in 250 µL of anhydrous MeCN.

2.0 mL of the 10 mg mL^{-1} gelatin solution was cooled to 4°C in an ice/water bath, and under gentle stirring 180 µL of sulforhodamine 101 acid chloride stock solution (1.8 mg of dye) was added. The resulting solution was stirred overnight in the dark and then dialyzed against 4 L of 10 mM sodium phosphate, 154 mM NaCl, pH 7.4, for 2 days, with at least 4 buffer

exchanges. After complete removal of the unreacted Texas Red, the solution was freeze-dried and stored at -20°C.

1.5 Binding of glycopolymers to CD206 CTLD4-7 and CR domains: ELISA assays.

For the ELISA assays, murine CTLD4-7-Fc or CR-Fc constructs were prepared from plasmid encoding the Fc-chimeric proteins as described by Martinez-Pomares *et al.*[11]

Mannosylated, galactosylated and galactose-3-O-sulfated glycopolymers solutions in 10 mM NaH_2PO_4, 154 mM NaCl, pH 7.4 (PBS), - 5.5 µM sugar unit concentration, prepared from 20 mg mL^{-1} stock solutions of glycopolymers in sterile and endotoxin–free water - were immobilized on 96 well microtitres plates (MAXISORP Nunc, Roskilde, Denmark) by overnight incubation at 4°C (200 µL per well). PBS was used as negative control, mannan and chondroitin sulfate A at 5.5 µM sugar unit concentration (calculated as molecular weight of mannose for mannan and the sum of D-glucuronic acid and GalNAc molecular weights for chondroitin sulfate A) as positive controls for mannosylated and galactose-3-O-sulfated glycopolymers, respectively.

Glycopolymers (GPs) indirect ELISA tests: wells were rinsed three times with 250 µL of TBS buffer (10 mM Tris-HCl, pH 7.5, 10 mM Ca^{2+}, 154 mM NaCl, 0.05% (w/v) Tween-20) and exposed to CTLD4-7-Fc or CR-Fc constructs (2 µg mL^{-1}, 50 µL $well^{-1}$) in TBS buffer at room temperature, for 90 min.

Inhibition ELISA tests: CTLD4-7-Fc or CR-Fc constructs (2 µg mL^{-1}) were pre-incubated on ice for 30 min with 25 mM solutions in TBS buffer of 2'-azidoethyl-O-α-D-mannopyranoside or 3-O-sulfo-2'-azidoethyl-O-β-D-galactopyranoside monovalent ligands, respectively. Then, 50 µL of either CTLD4-7-Fc or CR-Fc constructs/sugars solutions were added to the appropriate wells and incubated at room temperature for 90 min.

The plates were washed three times with 250 µL of TBS buffer and 50 µL of Goat anti-human IgG (Fcγ specific) alkaline phosphatase (AP) conjugate (1:1000 dilution in TBS buffer) were added to each well, and incubated at room temperature for further 60 min. The plates were washed three times with 250 µL TBS, and twice with 250 µL AP buffer (100 mM Tris-HCl, 100 mM NaCl, 1 mM $MgCl_2.6H_2O$, pH 9.5). AP activity was detected using 50 µL of 1 mg mL^{-1} p-nitrophenyl phosphate substrate solution in AP buffer. After 30 minutes the absorbance at λ = 405 nm was measured using an ELISA plate reader (Labsystems Multiskan EX, Finland).

1.6 Cell lines and culture conditions.

Chinese Hamster Ovary (CHO and $CD206^+$-CHO) cell lines[3] were maintained in Dulbecco's modified Eagle's medium/Ham's F-12 nutrients containing 10% Fetal Bovine Serum (FBS), 2 mM L-glutamine, 100 U mL^{-1} penicillin and 100 µg mL^{-1} streptomycin, at 37°C, 5% CO_2 and

95% relative humidity. Stable transductants for CD206$^+$-CHO were selected using 0.6 mg mL^{-1} geneticin.

Bone marrow-derived macrophages (BMDM) from WT and CD206$^{-/-}$ C57BL/6 mice were prepared from femurs and tibias using L929 cells conditioning media (LCCM) or M-CSF as source of macrophage colony stimulating factor. BMDM were cultured in 20 cm bacteriologic plastic (BP) petri dishes by re-suspension in 25 mL of R10 media (RPMI supplemented with 10% Fetal bovine serum, 15% LCCM or 50 ng mL^{-1} M-CSF, 2 mM L-glutamine, 100 U mL^{-1} penicillin and 100 μg mL^{-1} streptomycin). Three days after the seeding, 15 mL of R10 fresh media containing LCM or M-CSF were added to the plate. On day 6 the media was removed and replaced with 25 mL of fresh R10 media containing LCM or M-CSF. After 7 days in culture, the attached cells were washed with 10 mL of sterile phosphate-buffered saline (PBS) and collected using 10 mL of ice-cold PBS containing 10 mM EDTA for 10 min. BMDM were detached by gently pipetting, centrifuged at 233 g for 5 min, washed with 40 and 10 mL of opti-MEM® containing 2 mM L-glutamine, 100 U mL^{-1} penicillin and 100 U mL^{-1} streptomycin (complete opti-MEM®) and re-suspended in 10 mL of complete opti-MEM® at a concentration of 1.5x10^6 cells mL^{-1} for seeding.

Prior to carrying out the uptake inhibition experiments, the presence of CD206 at the cell membrane of WT macrophages was assessed by immunostaining with Alexa Fluor® 647 anti-mouse CD206 Antibody. 3.2x10^5 BMDM were resuspended in 400 μL of blocking buffer (5% inactivated goat serum, 0.5% BSA, 2 mM NaN_3, 5 mM EDTA in PBS) containing a 1:1000 dilution of Anti-CD16/CD32 antibody (clone 2.4G2, 2 mg mL^{-1}) and incubated on ice for 30 min. Cells were centrifuged at 1000 rpm using a benchtop centrifuge, for 5 min at 4 °C, re-suspended in 100 μL of blocking buffer containing 0.5 μg mL^{-1} of Alexa Fluor® 647 anti-mouse CD206 antibody or isotype control (Alexa Fluor® 647 Rat IgG2a,κ Isotype Ctrl) and incubated for 1 h at 4°C. Cells were washed three times with 1 mL of FACS buffer (0.5% BSA, 2 mM sodium azide, 5 mM EDTA in PBS) and resuspended in 500 μL of FACS buffer containing 2% paraformaldehyde. Cells were analysed on a MoFlo® Astrios™ (Beckman Coulter, Brea, Calif., USA) equipped with 7 lasers (355 nm; 405 nm; 488 nm; 532 nm; 561 nm; 592 nm; 640 nm). Laser settings used for the acquisition are reported as excitation-emission/bandwidth in nm. Fluorescence was detected using laser setting 640-671/30 for Alexa Fluor® 647. Untreated cells were also analysed to estimate cell auto-fluorescence. On average, in all preparations 94% of cells stained positively for CD206 expression. A representation of the gating strategy used is shown in Supplementary Figure S39.

1.6.1 Biocompatibility of glycopolymers

Biocompatibility studies were performed using the "*LDH Cytotoxicity Detection Kit*" (*Roche*) according to the manufacturer's instructions. CHO, CD206$^+$-CHO, WT or CD206$^{-/-}$

macrophages cells were seeded in 96-well plates (200 µL well^{-1}, 2x10^5 cell mL^{-1}) and cultured overnight at 37°C, 5% CO_2. Cells were treated with Oregon Green-labelled mannosylated, and galactose-3-*O*-sulfated glycopolymers solutions (100 µL well^{-1}) in complete opti-MEM® supplemented with 1% FBS (prepared from 20 mg mL^{-1} stock polymer solutions in sterile and endotoxin–free water), at 3.2-640 µM concentration of sugar repeating units. BMDM were treated for 2 or 24 h, and CHO cells for 24 h at 37°C. Maximum LDH release controls consisted of cells treated with 100 µL of media containing 1% Triton-X-100. Plates were centrifuged at 931 g for 5 min and 50 µL of each sample were transferred to a new plate and mixed with 50 µL of LDH working reagent. Plates were then incubated for 45 minutes at room temperature in the dark, and the absorbance at λ = 492 nm was recorded with a microplate reader, with reference wavelength at λ = 650 nm. The cytotoxicity (percentage of LDH release) was calculating using the formula (1) below, where "Sample Abs (492 nm)" is the absorbance at λ = 492 nm recorded for the cells treated with glycopolymers, "blank" is that of untreated cells, and "control" that of cells treated with 0.1% Triton-X-100:

$$Cytotoxicity\ (\%) = \frac{(\text{Sample Abs}(492\text{ nm}) - \text{Blank})}{(\text{Control} - \text{Blank})} x\ 100\ (1)$$

To remove the contribution of the Oregon Green-labelled glycopolymers to the measured absorbance at λ = 492 nm, the absorbance of polymer solutions in complete opti-MEM® supplemented with 1% FBS at all the concentration tested was measured, and subtracted to those of the corresponding LDH assay samples. Accordingly, 100 µL of each glycopolymer solution were placed in a 96 wells plate in absence of cells, and samples were processed as described above for the glycopolymer-CHO cells samples. The absorbance at λ = 492 nm of the final glycopolymer-only samples were subtracted to those of the corresponding glycopolymer-CHO cells samples.

All samples were run in triplicate and experiments were repeated twice.

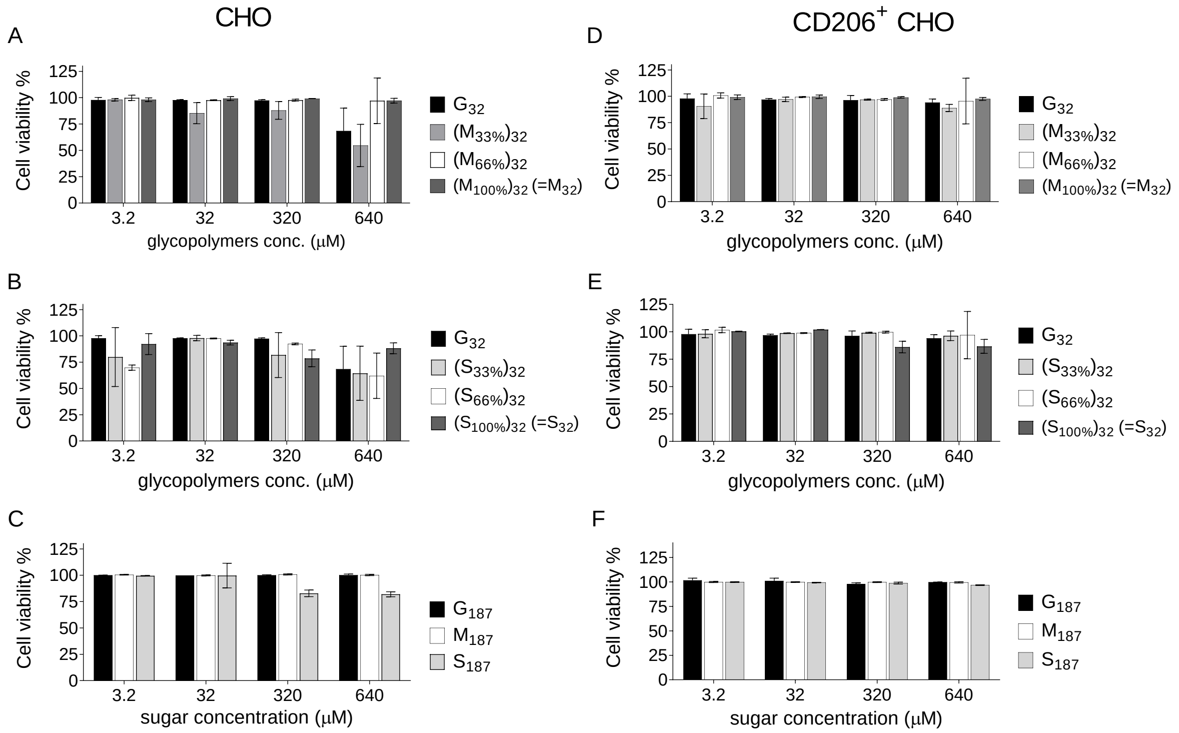

Figure S4. CHO cell viability following incubation with CD206 glycopolymer ligands. LDH assay: CHO (A, B, C) and CD206+-CHO (D, E, F) cells were treated with glycopolymers at various concentrations (3.2-640 µM of sugar repeating units), at 37°C, for 24 hours. Data shown are expressed as LDH release (%) compared to that of CD206 positive and negative control cells, and are the average of two independent experiments performed in triplicate.

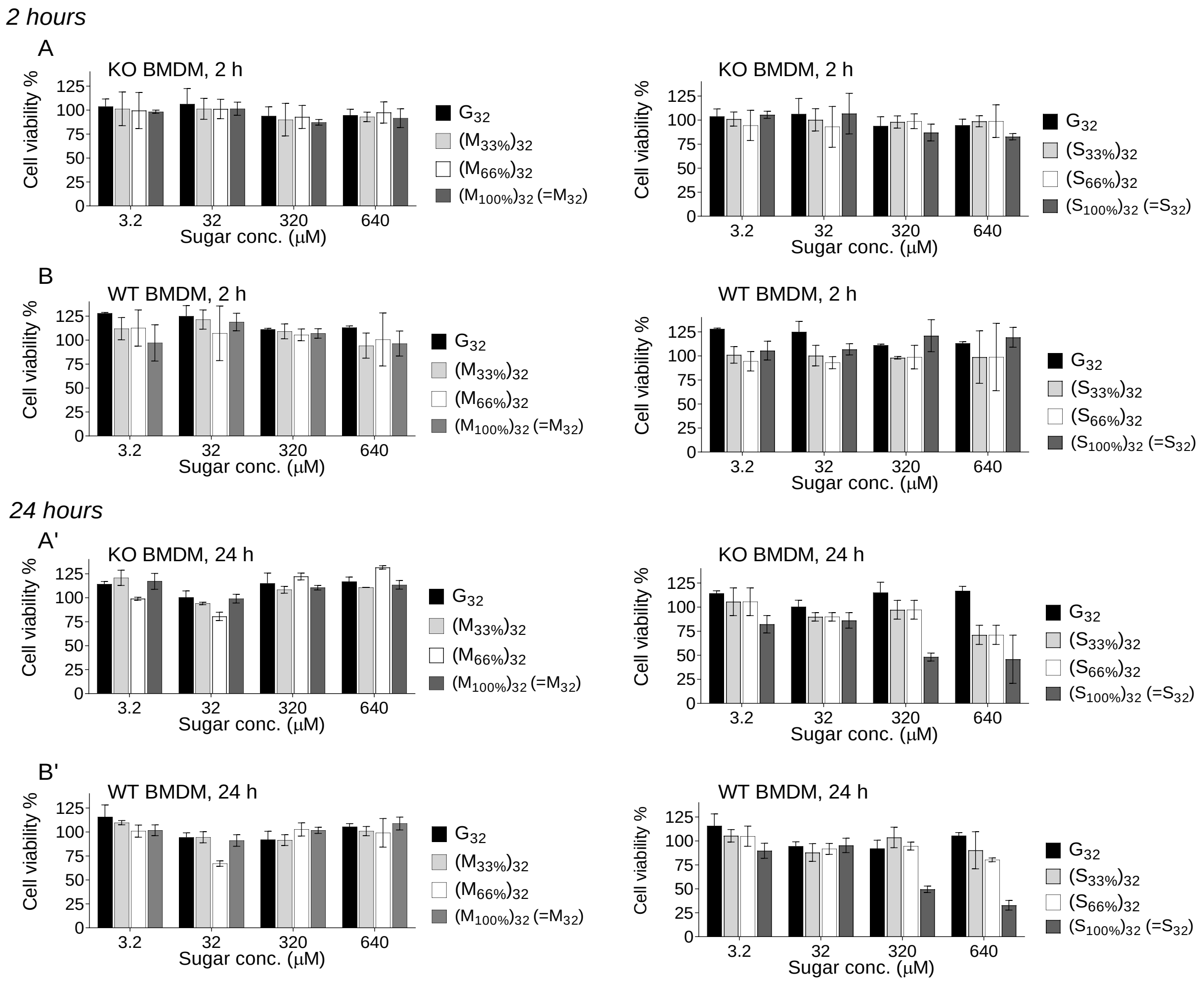

Figure S5. Viability of murine bone marrow-derived macrophages (BMDM) following incubation with CD206 glycopolymer ligands assessed by quantification of lactate dehydrogenase release (LDH assay). CD206-*knockout (KO)* (A, A') and WT (B, B') macrophages were treated with DP 32 mannosylated (left) and sulfated (right) glycopolymers (concentration: 3.2-640 μM of sugar repeating units), at 37°C, for 2 (I), or 24 (II) h. Data shown are expressed as cell viability (%) and are the average of two independent experiments performed in triplicate.

The assay was repeated on CHO and CD206$^+$-CHO cells, incubated for 30 min or 2 h with Oregon Green-labeled galactose-3-*O*-sulfated glycopolymers solutions, at 20.6 μM and 490 μM sugar units concentration. The medium was then discharged, and cells further incubated for 2, 24 or 48 h in complete opti-MEM® supplemented with 1% FBS before analysis.

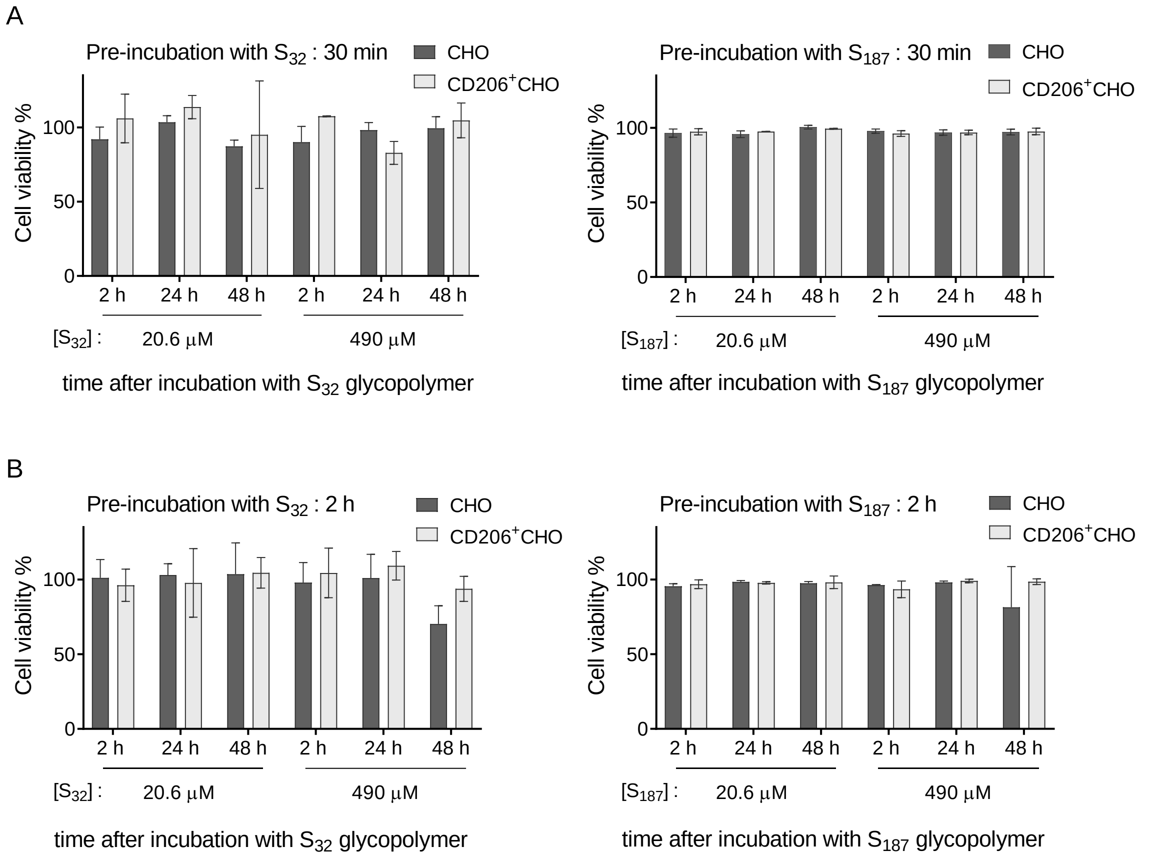

Figure S6. CHO and CD206⁺-CHO cells viability at 2, 24, and 48 h, following incubation with SO_4-3-Gal glycopolymers S_{32} and S_{187} for 0.5 or 2 h: LDH assay. CHO and CD206⁺-CHO cells were pre-incubated with S_{32} or S_{187} for (A) 30 min, or (B) 2 h. Glycopolymer-containing culture media were replaced with fresh media, and LDH assay was carried out after 2, 24, or 48 h. Concentrations of S_{32} and S_{187} are expressed in µM of sugar repeating units. Data shown are expressed as cell viability (%), and are the average of two independent experiments performed in triplicate.

1.6.2 Uptake of glycopolymers by CD206$^+$ and CD206$^-$ cell lines

CHO or CD206$^+$-CHO cells were seeded in a 24 wells plates (400 µL per well, 6.25 x 10^5 cells mL^{-1}) and cultured overnight at 37°C, 5% CO_2. Cells were washed with 2 x 500 µL of PBS, incubated for 30 min at 37°C, 5% CO_2 with complete opti-MEM® and then with complete opti-MEM® containing Oregon Green labeled glycopolymers [low molecular weight DP 32 glycopolymers (G_{32}; $(M_{33\%})_{32}$, $(M_{66\%})_{32}$, and $(M_{100\%})_{32}$; $(S_{33\%})_{32}$, $(S_{66\%})_{32}$, and $(S_{100\%})_{32}$) (1 µg mL^{-1}, 400 µL $well^{-1}$)]. After 30 or 60 min, the cells were rinsed three times with 1 mL of PBS, harvested using trypsin/EDTA solution diluted 1:1 in PBS and fixed in 2% paraformaldehyde in PBS. Untreated cells were used as negative controls.

Similar experiments were performed using WT and CD206$^{-/-}$ (BMDM). Cells (200 µL, 1.5x10^6 cells mL^{-1} in complete opti-MEM®) were transferred into sterile plastic tubes (BD Falcon) and incubated at 37°C for 30 min. 200 µL of 2 µg mL^{-1} glycopolymers solutions in complete opti-MEM® were added to the tubes and incubated for further 30 min at 37°C. Cells were washed

twice with 2 mL ice-cold complete opti-MEM®, re-suspended in 200 µL of the same media and fixed in 2% paraformaldehyde solution in PBS (200 µL).

Samples were analyzed using a Beckman Coulter FC500 Series equipped with an argon laser (λ = 488 nm excitation) and a red emitting diode (λ = 635 nm excitation), with CXP acquisition version 2.2 (Beckman Coulter). The mean fluorescence intensity (MFI) was detected on the standard analysis optical filters FL1 (λ = 525 nm) and at least 2 x 10^4 cells were acquired for single staining.

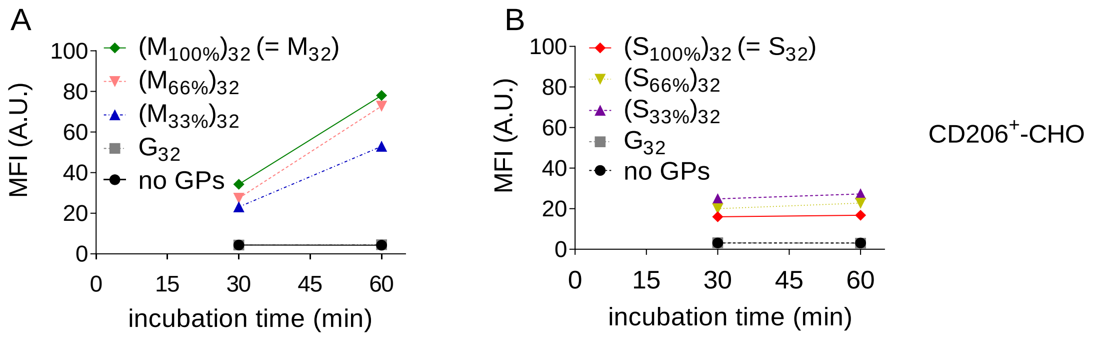

Figure S7. Relative uptake of Oregon Green (OG)-tagged DP 32 glycopolymers (GPs) with different mannose content, under different incubation times and polymer concentration, as quantified by flow cytometry. (A) and (B): time-dependent uptake of glycopolymers (1.0 µg mL^{-1}) by $CD206^+$-CHO cells, as quantified by FACS, and data are presented as mean ± s.d. of one experiment performed in duplicate. For some points error bars are smaller than data symbols. MFI indicates sample mean fluorescence intensity and is expressed in arbitrary units (A.U.).

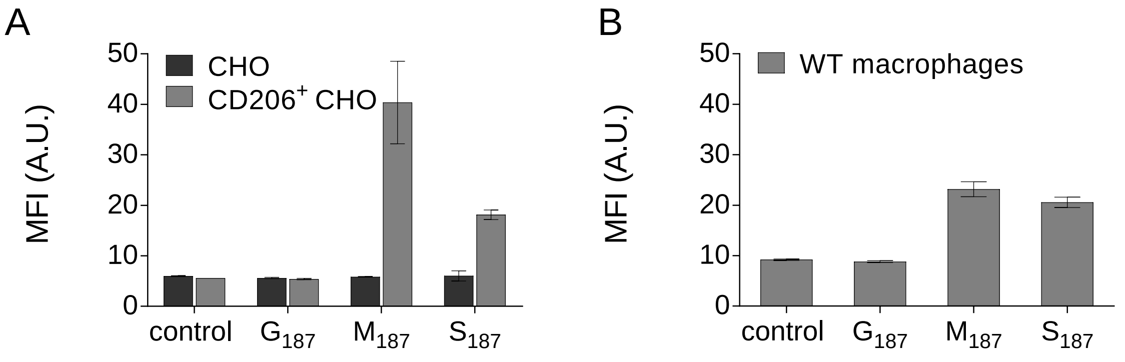

Figure S8. Relative uptake of Oregon Green (OG)-tagged DP 187 glycopolymers by different cell lines, as assessed by flow cytometry. (A) $CD206^+$-CHO and $CD206^-$-CHO cells were incubated with 1.0 µg mL^{-1} glycopolymers concentration (corresponding to 2.7 and 2.1 µM sugar unit for M_{187} and G_{187}, and S_{187}, respectively) for 30 minutes at 37°C. Data are reported as mean ± s.d. of two independent experiments performed in duplicates. (B) WT bone marrow derived macrophages were incubated for 30 minutes at 37°C with 1.0 µg mL^{-1} (corresponding to 2.7 and 2.1 µM sugar unit for M_{187} and G_{187}, and S_{187}, respectively) solutions of glycopolymer. Data are reported as mean ± s.d. of a single experiment performed in duplicate. Untreated cells were used as control in both sets of experiments. MFI indicates sample mean fluorescence intensity, and is expressed in arbitrary units (A.U.).

1.6.3 Inhibition of CD206 endocytic activity *in vitro*: blocking cell uptake of Texas Red-tagged gelatin (TR-gelatin), and mannosylated M_{32}-Nile Blue ligands.

Uptake inhibition experiments. CHO and $CD206^+$-CHO cells were incubated with DP 32 (G_{32}, M_{32}, and S_{32}), and DP 187 (G_{32}, M_{32}, and S_{32}) glycopolymers for 30 min or 2 h, then:

i) the glycopolymer-containing cell media were replaced with medium containing fluorescent Texas Red-tagged gelatin (TR-gelatin), an alternative ligand for CD206 (*uptake inhibition* experiments, Figure 6), and the cells incubated under these conditions for 2 h, or

ii) TR-tagged gelatin was added without removing the glycopolymer-containing medium, and cells were co-incubated with glycopolymers and TR-gelatin for 2 hours (*uptake inhibition/co-incubation* experiments, Figure S9).

Experimental Procedure.

CHO cells: inhibition of TR-gelatin uptake. CHO and $CD206^+$-CHO cells were seeded in a 24 wells plate (400 µL per well, 6.25 x 10^5 cells mL^{-1}) and cultured overnight at 37°C, 5% CO_2. Cells were washed twice with 500 µL of PBS, and incubated in complete opti-MEM® for 30 min. Media was then replaced with solutions of Oregon Green-labeled glycopolymers in complete opti-MEM® (400 µL per well, 490 µM sugar units concentration) and cells were cultured for 30 min or 2 h.

Uptake inhibition experiments: after initial incubation with glycopolymers, the medium was removed, the cells were washed three times with 1 mL PBS and TR-gelatin (400 µL, 80 µg mL^{-1}) in complete opti-MEM® was added to the wells and cells were incubated with TR-gelatin for 2 h at 37°C in the dark. After the incubation, cells were rinsed three times with 1 mL PBS, collected using trypsin/EDTA diluted 1:1 in PBS, and fixed with 2% paraformaldehyde in PBS before FACS analysis. Fluorescence was detected on FL1 (λ = 525 nm, Oregon Green detection) and FL3 (λ = 620 nm, Texas Red detection). A minimum of $1x10^4$ cells were acquired per sample. Untreated cells were also analysed to estimate cell auto-fluorescence. Cells treated with only TR-gelatin for 2 h, without pre-incubation with glycopolymers, were used as positive controls, and their MFI readings set as 100% of ligand uptake. Compensation to account for the overlap of the emission of Oregon Green into TR-gelatin channel was carried out by using FACS fluorescence readings from cells treated with only Oregon Green-tagged glycopolymers (without subsequent addition of TR-gelatin). Samples were analysed as described in the ”Uptake of glycopolymers by $CD206^+$ and $CD206^-$ cell lines” section.

Uptake inhibition/co-incubation experiments: TR-gelatin (80 µg mL^{-1}) was added to the cells without removing the glycopolymers-containing medium (final volume: 405 µL) and then treated as described above.

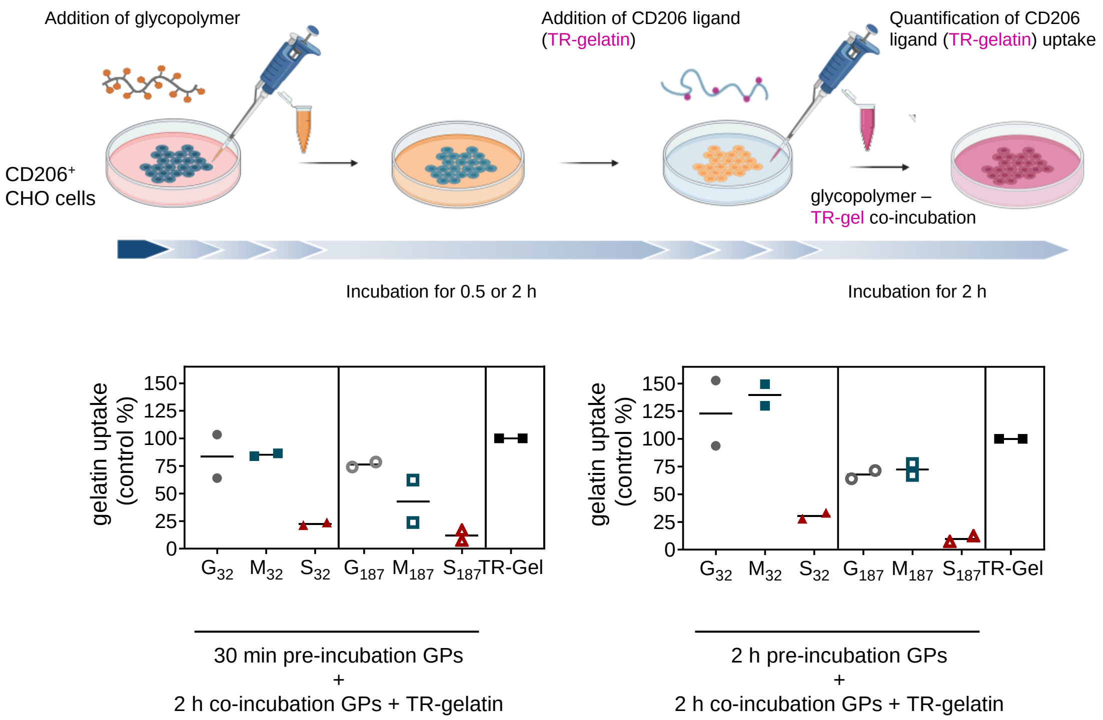

Figure S9. Uptake inhibition/co-incubation experiments. Cells were pre-treated with glycopolymers (490 µM in sugar binding units) for 30 min or 2 h followed by addition of TR-gelatin for 2 h (final concentration 80 µg mL^{-1}). Control cells were incubated over the same period with cell medium in the absence of glycopolymers. Data are expressed as percentage of gelatin uptake compared to that of $CD206^{+}$-CHO cells not pre-treated with glycopolymers (right column of each panel, TR-gelatin). Data are reported as mean ± s.d. of two independent experiments performed in duplicates.

CHO cells: inhibition of M_{32}-Nile Blue uptake. An analogous experiment was performed using Nile blue-labeled mannosylated polymer M_{32}-Nile Blue instead of TR-gelatin, as the CD206 ligand of choice. Cells were seeded as described above and then Oregon Green-labelled glycopolymers (see below) diluted in complete opti-MEM® (prepared from 20 mg mL^{-1} stock polymer solutions in sterile and endotoxin–free water) were added to the wells (400 µL $well^{-1}$, 490 µM sugar units concentration). Cells were incubated with the glycopolymers for 2 h, then:

Uptake inhibition experiments: after initial incubation with G_{32} or S_{32} glycopolymer for 2 h, the medium was removed, the cells were washed three times with 1 mL PBS, then M_{32}-Nile Blue in medium (400 µL, 165 µM of sugar units) was added to the wells. Cells treated with only M_{32}-Nile Blue for 2 h, without pre-incubation with galactose or galactose 3-*O*-sulfated glycopolymers, were used as positive controls, and their MFI readings set as 100% of ligand uptake. After 2 h cells were rinsed three times with 1 mL PBS, trypsinized with 0.25% trypsin/EDTA diluted 1:1 in PBS, harvested, and fixed with 2% paraformaldehyde in PBS. Cell fluorescence was assessed by FACS analysis on FL1 (λ = 525 nm, Oregon Green detection) and FL4 (λ = 675 nm, Nile blue detection). Fluorescence of samples was reported as % relative to that of the positive control.

Uptake inhibition/co-incubation experiments: after initial incubation with G_{32} or S_{32}, M_{32}-Nile Blue (4.04 µL, 16.5 mM sugar units) was added to the wells without removing the initial glycopolymers-containing medium. Cells were co-incubated with M_{32}-Nile Blue and G_{32}/S_{32} containing medium for further 2 h at 37°C in the dark, and then treated as described above. Cells treated with only M_{32}-Nile Blue for 2 h, without pre-incubation with G_{32} or S_{32} glycopolymers, were used as positive controls, and their MFI readings set as 100% of ligand uptake. Fluorescence was assessed by FACS analysis on FL1 (λ = 525 nm, Oregon Green detection) and FL4 (λ = 675 nm, Nile blue detection). Fluorescence of samples was reported as % relative to that of the positive control.

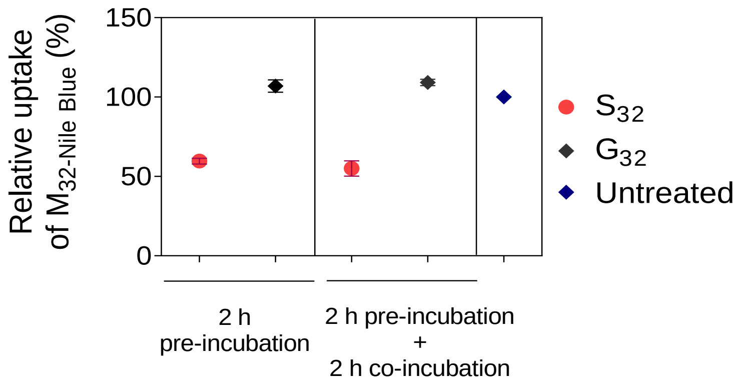

Figure S10. M_{32}-Nile Blue uptake inhibition experiments. *Uptake inhibition experiments:* CD206$^+$-CHO cells were pre-treated with S_{32} or G_{32} glycopolymers (490 µM in sugar repeating units) for 2 h. Then the medium was replaced with M_{32}-Nile Blue containing medium (left panel) or M_{32}-Nile Blue (165 µM in sugar repeating units) was then directly added to the wells and cells were incubated for further 2 h. Uptake is reported relative to that of positive CD206$^+$-CHO control cells incubated with M_{32}-Nile Blue for 2 h, without pre-treatment with other glycopolymers ('untreated' column) followed by quantification of M_{32}-Nile Blue uptake by flow cytometry (λ = 675 nm, Nile Blue detection). Control cells were incubated over the same period with cell medium in the absence of glycopolymers. Data are reported as mean ± s.d. of two biological replicates (N=1).

Inhibition of CD206-mediated uptake of TR-gelatin by BMDM. WT or CD206$^{-/-}$ (KO) BMDM in complete opti-MEM® (100 µL, 1.6x10^6 cells mL^{-1}) were transferred into sterile BD Falcon plastic tubes and incubated at 37°C for 30 min. Glycopolymers solutions in complete opti-MEM® (final concentration 49 or 490 µM of sugar units) were added to the tubes and incubated for further 1 h at 37°C. The glycopolymer-containing medium was removed, cells were washed twice with 200 µL ice-cold complete opti-MEM®, re-suspended in 200 µL of a TR-gelatin solution in complete opti-MEM® (10 µg mL^{-1}), and incubated for 1 h at 37°C. Cells were washed twice with 200 µL ice-cold complete opti-MEM®, gently re-suspended in 400 µL of complete opti-MEM® and fixed with 50 µL of a 9% paraformaldehyde solution in PBS. Samples were analyzed using a Beckman Coulter Astrios EQ Cell Sorter, with Summit version 6.2.3 (Beckman Coulter). The median fluorescence intensity (MFI) was detected on FL17 (488-513/26) for Oregon Green

and on FL24 (561-614/20) for Texas Red, and at least 5x10^3 cells were acquired for each sample. All experiments were performed in duplicate and repeated at least twice.

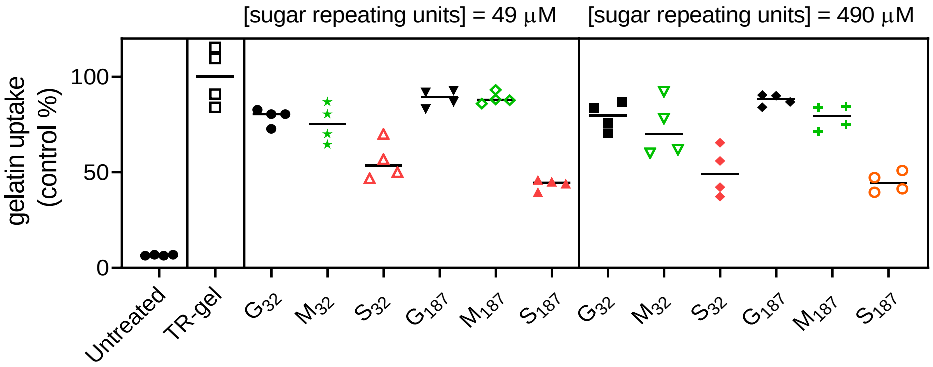

Figure S11. Inhibition of CD206-mediated uptake of TR-gelatin by murine bone marrow-derived macrophages (BMDM). Cells were pre-treated with glycopolymers (49 or 490 µM in sugar repeating units) for 1 h. The medium was then replaced with a solution of TR-gelatin in opti-MEM® (final concentration 10 µg mL^{-1}) and incubated for 1 h. Control cells ('untreated') were incubated over the same period with cell medium in the absence of glycopolymers and TR-gelatin. Data are expressed as percentage of gelatin uptake compared to that of BMDM not pre-treated with glycopolymers (TR-Gel, positive control). Data are reported as mean ± s.d. of two independent experiments performed in duplicates.

1.6.4 Inhibition of CD206 endocytic activity *in vitro* vs. time: time course experiments

CHO and CD206$^+$-CHO cells were seeded in a 24 wells plate (400 µL per well, 3.75x10^5 cells mL^{-1}) and cultured overnight at 37°C, 5% CO_2. Cells were washed twice with 500 µL of PBS, and incubated with complete opti-MEM® for 30 min. The medium was removed and Oregon Green-labeled S_{32} or S_{187} glycopolymers were added to the wells (400 µL well^{-1}, 490 µM sugar units concentration in complete opti-MEM®). After 2 h of incubation at 37°C the cells were washed three times with 1 mL PBS, and 400 µL of complete opti-MEM® were added to each well. At scheduled times (2, 4, 6, 8, 16, 24, 30, 36, 48 h) the medium was replaced with TR-gelatin solution (400 µL, 80 µg mL^{-1} in complete opti-MEM®) and the cells were further incubated for 1 h. After incubation with TR-gelatin, cells were rinsed three times with 1 mL PBS, harvested and fixed with 2% paraformaldehyde in PBS as above. FACS analysis was carried out as described in "Inhibition of CD206-mediated uptake of TR-Gelatin by BMDM".

1.6.5 Quantification of total and plasma membrane CD206 after treatment of CD206$^+$-CHO cells with glycopolymers

Quantification of total cell CD206: Western blot analysis. CD206$^+$-CHO cells were seeded in 24 well-plates (400 µL per well, 6.25x10^5 cells mL^{-1}) and cultured overnight at 37°C, 5% CO_2. Cells were washed twice with PBS (500 µL per well) and incubated for 30 min with complete opti-MEM®. The medium was then replaced with solutions of 100% mannosylated (M_{32} and

M_{187}) or sulfated (S_{32} and S_{187}) Oregon Green-labeled glycopolymers in complete opti-MEM® (20.6 or 490 µM of sugar repeating units, 400 µL per well). After 30 min or 2 h incubation at 37°C, the cells were washed three times with PBS (1 mL per well) and lysed in 200 µL of ice-cold lysis buffer (2% Triton X-100, 10 mM Tris-HCl, 150 mM NaCl, 2 mM EDTA, pH 7.4), containing protease inhibitors (Roche Diagnostics), for 30 min at 4°C. Lysates were harvested and centrifuged in a tabletop centrifuge at 4°C first at 2000 rpm to eliminate nuclei and then at 13000 rpm. Supernatants were stored at -20°C. Protein concentrations were quantified using the bicinchoninic acid assay. Cell lysates (2.5 µg protein content) were separated on 6% sodium dodecyl sulfate-polyacrylamide gel electrophoresis (SDS-PAGE) gels under non-reducing conditions and transferred to nitrocellulose membrane using a Trans Blot® Cell apparatus (Trans-Blot electrophoretic Transfer Cell – Bio Rad) overnight at 200 mA. After blocking with blocking buffer (0.1% tween 20, 5% low fat milk in PBS) for 1 h, membranes were incubated with anti-CD206 mAb (MR5D3, 9 2 µg mL^{-1} in blocking buffer) for 1 h. Membranes were washed 3 times with 0.1% tween 20 in PBS and CD206 detected using an HRP-conjugated goat-anti-Rat IgG (Source Bioscence) (1:1000 dilution in blocking buffer, 1 h at room temperature). Bound antibodies were visualized using an enhanced chemiluminescence system (ECL system, Amersham Pharmacia Biotech, Bucks, UK) and recorded on an X-ray film.

Quantification of CD206 on plasma membrane by FACS. $CD206^+$-CHO cells were seeded in 12 wells/plate (1 mL $well^{-1}$, $650x10^3$ cells mL^{-1}) and grown overnight at 37°C, 5% CO_2. Cells were washed twice with PBS (500 µL per well) and incubated with complete opti-MEM®. After 30 min, the medium was replaced with solutions of 100% mannosylated (M_{32} and M_{187}) or sulfated (S_{32} and S_{187}) Oregon Green-labeled glycopolymers in complete opti-MEM® (490 µM sugar repeating units, 1 mL per well). After 2 h incubation at 37°C, the cells were washed 3 times with washing buffer (PBS containing 0.5% BSA, 5 mM EDTA, and 2 mM NaN_3), harvested with non-enzymatic cell dissociation buffer (Sigma-Aldrich) on ice, dissociated by gentle pipetting and transferred to a sterile tube. Cells were washed twice with PBS (1 mL per sample), re-suspended in PBS (200 µL per sample) and fixed with 2% paraformaldehyde in PBS for 30 min at 4°C. Non-specific binding was blocked by incubation with blocking buffer (5% heat-inactivated goat serum, 0.5% BSA and 5mM EDTA in PBS, 400 µL per sample) for 30 min at 4°C. Cells were then incubated with primary Ab (MR5D3 or IgG2a isotype control, 5 µg mL^{-1} diluted in blocking buffer) for 40 minutes at room temperature. After incubation cells were washed 3 times in washing buffer and labeled secondary Ab (Alexa fluor® 647-conjugate goat anti-Rat IgG (Biolegend), diluted 1:1000 in blocking buffer) was added. After 45 min at room temperature, the cells were washed as described above, re-suspended in 400 µL of PBS containing 2% paraformaldehyde and analyzed by FACS. Untreated $CD206^+$-CHO cells were

used as positive control, and their fluorescence reading was taken as 100% membrane expression of CD206.

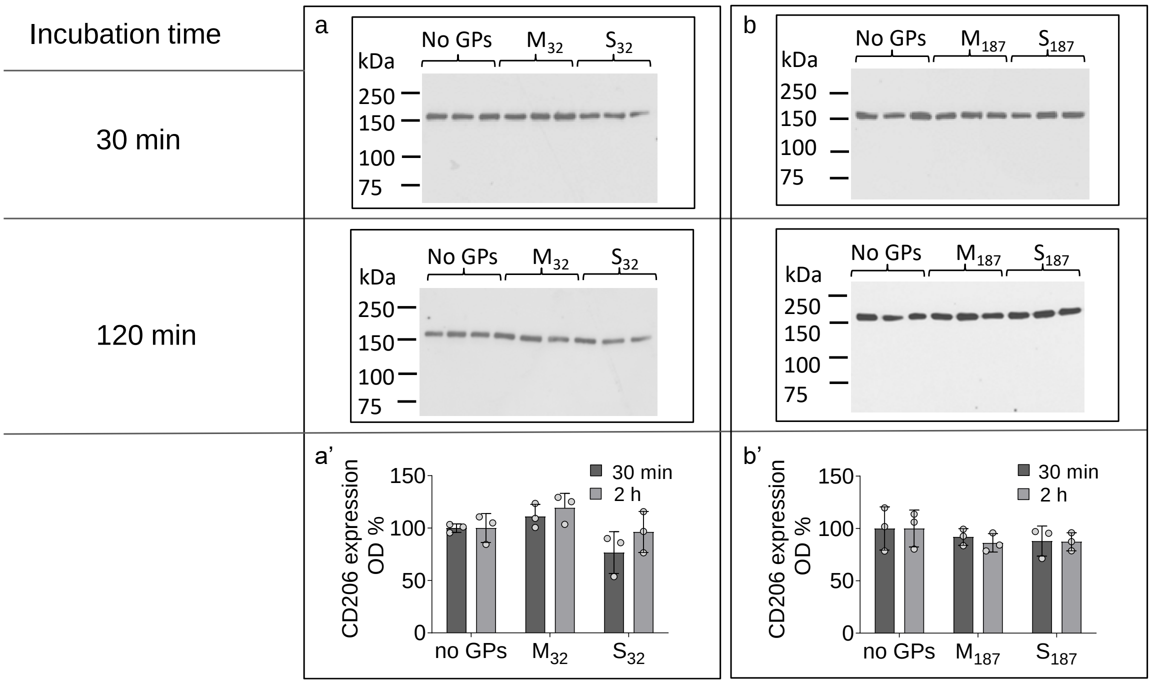

Figure S12. Quantification of total CD206 in CD206+-CHO cells after treatment with glycopolymer ligands. Western blot analysis of cell lysates from CD206+-CHO cells treated with low (M_{32} and S_{32}, panel a) and high (M_{187} and S_{187}, panel b) molecular weight glycopolymers at a 490 µM concentration of sugar repeating units. Untreated CD206+-CHO cells were used as controls. CD206 expression was estimated by optical densitometry (OD) of western blots gels (panel A' and B'). Data are representative of two independent experiments performed in duplicate or triplicate. Panels A and B show a complete representation of gels shown in Figure 3 of the manuscript.

1.6.6 Glycopolymers internalization and intracellular trafficking: confocal microscopy studies

These experiments were carried out to confirm that glycopolymers were indeed internalized by CD206+-cells and not simply associated to CD206 at the cell membrane, and to follow intracellular trafficking of glycopolymers after CD206-mediated endocytosis. Accordingly, $2x10^5$ CD206+-CHO cells were allowed to adhere to 10x10 mm glass slide (pre-treated with HCl 1 N and extensively rinsed with RPMI 1640) overnight. Next, cells were incubated for 2 h with solutions of 100% mannosylated (M_{32}), galactose 3-*O*-sulfated (S_{32}), and control galactose (G_{32}) glycopolymers, diluted in complete opti-MEM® (8.1 µM of polymer concentration, 259 µM of sugar repeating units), prepared from 20 mg mL^{-1} stock polymer solutions in sterile and endotoxin–free water. Then,

- *To monitor glycopolymer internalization*: after gentle washing with PBS (3 x 1 mL) cells were fixed with 4% paraformaldehyde in PBS for 10 minutes at ambient temperature, samples were rinsed with PBS (3 x 1 mL) and directly stained with 1 µg mL^{-1} HOECHST solution in PBS for 15 min at room temperature.
- *To follow glycopolymer trafficking:* after gentle washing with PBS (3 x 1 mL) cells were either immediately fixed with 4% paraformaldehyde in PBS for 10 minutes at ambient temperature or incubated for further 2 h with complete medium before fixation. Samples were rinsed with PBS (3 x 1 mL) and permeabilized in 0.5% Tween 20 with 4% BSA in PBS for 10 minutes at room temperature. Cells were washed with 0.1% Tween 20 in PBS (PBS-T) and blocked with 10% heat-inactivated goat serum in PBS (blocking buffer) for 1 h at room temperature. Finally, cells were stained with primary Abs (2 µg mL^{-1} of MR5D3, or anti-EEA1 or anti-LAMP1 diluted in blocking buffer) by incubation for 1 h at room temperature. Subsequently, cells were washed three times with 1 mL PBS-T and incubated with Alexa fluor® 647 conjugated secondary antibody (1:1000 diluted in blocking buffer, goat anti-Rat for CD206, goat anti-Rabbit for EEA1 or LAMP1) for an additional 60 min at room temperature. Cells were washed as described above and nuclei were stained with 1 µg mL^{-1} HOECHST solution in PBS for 15 min at room temperature.

In both sets of experiments, after the last cycle of washing, coverslips were mounted with DAKO fluorescent mounting medium and viewed by confocal microscopy using a Zeiss LSM 700 microscope (Carl Zeiss, Heidelberg, Germany) and ZEN2.3 (blue edition) software for images elaboration. Unstained control $CD206^{+}$-CHO cells were analyzed to assess cells autofluorescence. Anti-Rat or Anti-Rabbit secondary antibodies concentrations were optimized using a fluorescence microscope to avoid unspecific staining.

A 63x oil objective was used for acquiring all images. Section images in the z-dimension were collected. The quantitative colocalization analysis was performed by using ZEN 2010 confocal microscopy software (Carl Zeiss). Analysis was carried out on the best optical section in the z-stack and in sections above and below this plane. Singles cells in 4 different fields were compared for each analysis (12 cells analyzed per sample). The experiments were performed in duplicate.

1.7 Quantification of the avidity of glycopolymers for CD206 - Surface Plasmon Resonance (SPR) analysis

Binding experiments were performed by Surface Plasmon Resonance (SPR) on a BIAcore 3000 (Biacore Life Science). Approximately 1300-2000 response units (RU) of soluble CD206 (sCD206) was immobilised on a CM-5 sensor chip surface by amine coupling, to achieve a Rmax of approximately 100 RU during kinetic binding experiments (2).

$$\mathrm{R}_{max} = \frac{\text{analyte MW}}{\text{ligand MW}} x\, \mathrm{R}_{ligand} \quad (2)$$

Where "R_{max}" is the maximum analyte binding capacity of the surface, expressed in RU, "analyte MW" is the analyte molecular weight (molecular weight of polymer sugar repeating units), "ligand MW" is the molecular weight of the ligand (soluble CD206, sCD206) and "R_{ligand}" is the signal measured for the amount of immobilised protein in RU.

After surface activation by treatment with 140 µL EDC/NHS 1:1 (v/v) mixture in DI water (0.4 M EDC and 0.1 M NHS stock solution), 70 µL of 30 µg mL^{-1} sCD206[12] solution in 10 mM HEPES, 5 mM $CaCl_2$, 0.005% Tween-20, 150 mM NaCl, pH 7.4 (running buffer) were covalently coupled to the CM-5 sensor chip. Then, 190 µL of ethanolamine was applied to quench the excess of activated NHS esters functionalities at the sensor chip surface, followed by extensive wash with 1 M NaCl. The control channel was activated with 140 µL EDC/NHS 1:1 (v/v) mixture in DI water (0.4 M EDC and 0.1 M NHS stock solution), then treated with 190 µL of ethanolamine, and finally washed with 1 M NaCl. All experiments were carried out at 25°C. Glycopolymers at various concentrations of sugar repeating units - 32 µM to 16 mM for M_{32} and G_{32} glycopolymers, 32 µM to 8 mM for S_{32} glycopolymers and 32 to 2400 µM for G_{187}, M_{187}, and S_{187} - in running buffer at pH 7.4, 6.5 and 6 were injected for 120 s at 20 µL min^{-1} flow rate. For mannosylated glycopolymers flow cell regeneration was performed by injection of 20 µL of 10 mM HEPES, 5 mM EDTA, 0.005% tween-20, 150 mM NaCl, pH 7.4, and running this as described above for the glycopolymer samples; for sulfated and galactosylated glycopolymers regeneration was achieved by setting 20 minutes as dissociation time. Sensorgrams were analyzed using the BIAevaluation 4.1 software. Data to obtain the binding curves shown were globally fitted to a simple Langmuir model for a 1:1 ligand-binding model, simultaneously fitting association and dissociation in the experimental curves at different concentrations of glycopolymers.

Table S2. Binding of glycopolymers to sCD206 at different pH values, as assessed by SPR (BIAcore®).

**Glycopolymer**[a]	**K_D (M)**		
	**pH 7.4**[b]	**pH 6.5** [b]	**pH 6.0** [b]
G_{32}	$1.51 \cdot 10^{-3}$	-	-
$(M_{33\%})_{32}$	$1.52 \cdot 10^{-4}$	-	-
$(M_{66\%})_{32}$	$1.70 \cdot 10^{-4}$	-	-
$(M_{100\%})_{32}$ (=M_{32})	$4.98 \cdot 10^{-5}$	$2.51 \cdot 10^{-4}$	-
$(S_{33\%})_{32}$	$5.24 \cdot 10^{-6}$	-	-
$(S_{66\%})_{32}$	$1.09 \cdot 10^{-5}$	-	-
$(S_{100\%})_{32}$ (=S_{32})	$6.36 \cdot 10^{-6}$	$7.33 \cdot 10^{-8}$	-
G_{187}	$4.15 \cdot 10^{-2}$	-	-
M_{187}	$8.4 \cdot 10^{-6}$	$3.55 \cdot 10^{-5}$	$5.88 \cdot 10^{-4}$
S_{187}	$3.33 \cdot 10^{-6}$	$4.21 \cdot 10^{-7}$	$3.77 \cdot 10^{-7}$

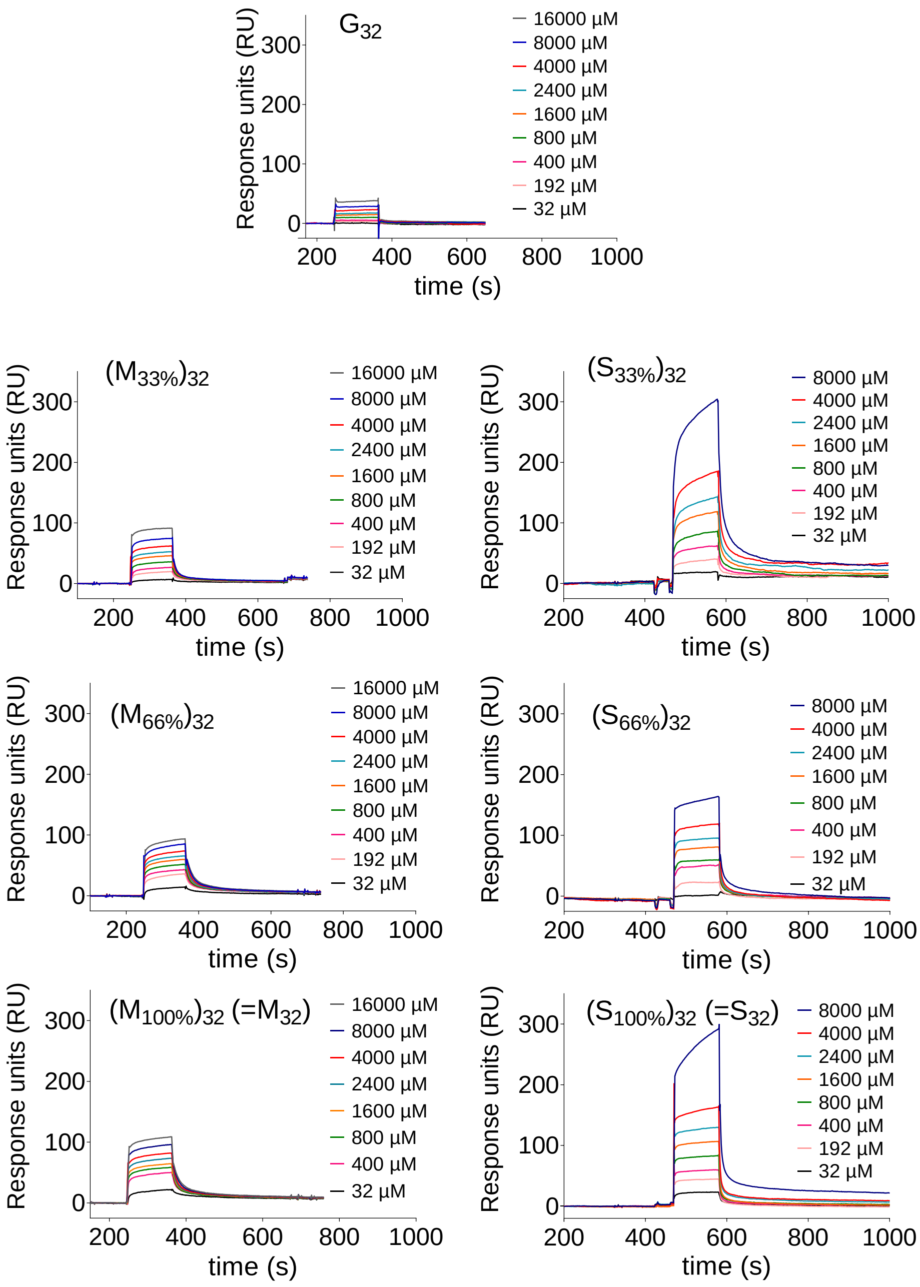

Figure S13. Quantification of binding of DP 32 glycopolymers to CD206 by surface plasmon resonance (SPR). Binding of DP 32 galactosylated, mannosylated and sulfated glycopolymers to soluble CD206 (sCD206)-coated sensor chips in 10 mM HEPES, 5 mM $CaCl_2$, 0.005% tween-20, 150 mM NaCl, pH 7.4, was quantified by SPR (BIAcore®). The signals from the reference channel were subtracted to all sensograms. Glycopolymer samples were injected at concentrations of polymer sugar repeating units of 0.032-16 mM for galactosylated and mannosylated glycopolymers, and of 0.032-8.0 mM for galactose-3-*O*-sulfated glycopolymers.

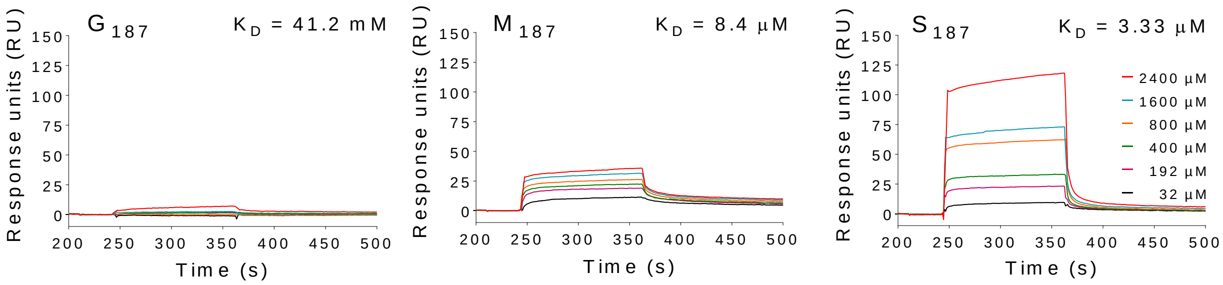

Figure S14. Quantification of binding of DP 187 glycopolymers to CD206 by surface plasmon resonance (SPR). Binding of G_{187}, M_{187}, and S_{187} to sCD206-coated sensor chip in 10 mM HEPES, 5 mM $CaCl_2$, 0.005% tween-20, 150 mM NaCl, pH 7.4, was quantified by SPR (BIAcore®). The signals from the reference channel were subtracted to all sensorgrams. Glycopolymer samples were injected at concentrations of polymer sugar repeating units of 0.032-2.4 mM.

1.8 Animals

Wild type (WT) and $CD206^{-/-}$ C57BL/6J mice (Charles River laboratory and kindly provided by Dr. M. Nussenzweig, Rockefeller University) were kept under specific pathogen-free conditions and used at 8-12 weeks of age. Mice were housed at Queen Medical Centre, Central Animal House, University of Nottingham Medical School. All animals were handled in accordance with institutional guidelines issued by the Home Office, United Kingdom (approval reference number 000088).

For in vivo experiments C57BL/6J mice were housed at the Department of Pharmaceutical and Pharmacological Sciences at the University of Padova under controlled light/dark conditions and food and water was provided ad libitum. All experiments were performed in compliance with the Italian laws and policies (D.L. n°26 14th March 2014) and with the guidelines established by the European Community Council Directive n° 2010/63/UE. The study was approved by the Institutional Review Board for Animal Research (Organismo Preposto al Benessere Animale, OPBA) of the University of Padua and by the Italian Ministry of Health (authorisation number 499/2019-PR).

1.8.1 *In vivo* inhibition of TR-gelatin uptake by $CD206^+$ liver cells.

C57BL/6 female mice (n=14, 19 g, 8 weeks old) were injected i.p. with 100 µL of PBS, or S_{187} or G_{187} solution in PBS at a polymer chain concentration of 400 µM. After 4 h, mice were injected i.p. with 100 µL of a 1 mg mL^{-1} TR-gelatin solution in PBS (treatment) or PBS alone (PBS control). 3 mice were used for each treatment group (PBS + TR-Gelatin, or glycopolymers + TR-Gelatin) and two for the PBS control. After further 2 h mice were deeply anesthetised with 0.1 mL of a solution of Zoletil (25 mg mL^{-1}), Xylor (2.5 mg mL^{-1}), in 0.9 % NaCl injected i.p.. Anesthetised mice blood was removed by cardiac withdrawal. Accordingly, a 1 mL syringe was rinsed with a heparin solution (1000 IU mL^{-1}, in 25 mM HEPES buffered

saline) and 100 µL of the same solution were placed in each collecting vial. Prior liver collection animals underwent heart perfusion with 60 mL PBS. Heart perfusion was performed to wash out the blood from the liver to eliminate TR-gelatin and glycopolymers still in circulation.

1.8.2 Quantification and imaging of Glycopolymers and TR-gelatin in liver

One liver lobe was accurately weighted, cut in small pieces with the help of scissors and pulverized with a mortar and pestle in liquid nitrogen. The homogenate was resuspended in 10 mL per liver fragment of RIPA buffer (50 mM Tris, pH 7.4, 150 mM NaCl, 1% Triton X-100, 0.5% sodium deoxycholate) containing cOmplete^{TM} protease inhibitors and DNAse (10 µg mL^{-1}), gently mixed by pipetting and left on a roller shaker for 3 h at 4°C. Then the suspensions were centrifuged at 14,000 rpm and 4°C for 30 min in a benchtop centrifuge, the supernatants were collected and filtered through a 0.45 µm sterile filter to remove tissue debris. The supernatants were analyzed by spectrofluorimetric measurement recording the emission spectra from 610 to 700 nm (λ_{ex} = 596 nm) for TR-gelatin and from 515 to 700 nm (λ_{ex} = 495 nm) for Oregon Green-tagged glycopolymers G_{187} and S_{187}. Raw data were normalized to tissue weight. The remaining liver tissue was fixed *ex vivo* in 4% paraformaldehyde in PBS for 30 min at 4° C, embedded in Optimal Cutting Temperature (O.C.T.) and stored at -70°C. The tissue was cryo-sliced into 10 µm slices and placed on microscope glass slides (Superfrost® Plus by Thermo Scientific). Specimens were washed with ethanol 70% to remove O.C.T., rinsed with PBS, saturated with blocking solution (2% BSA, 15% goat serum, 0.25% gelatin, 0.20 glycine, 0.5% Triton X-100 in PBS) and immunostained with either anti-CD206 (15-2) antibody (1:200 dilution, 1 µg mL^{-1} final concentration) or CD31 antibody (1:100 dilution), to stain liver endothelial cells, diluted in blocking solution. Sections were rinsed 3 times with PBS for 10 min. An anti-mouse Alexa fluor® 647 secondary antibody was diluted in blocking solution (1:200) and incubated with the tissue sections for 45 min in the presence of 15 µg mL^{-1} of Hoechst for nuclei staining. Sections were rinsed with PBS, the cover slips were mounted on glass slides with DAKO® mounting media and sealed with nail polish. Images were acquired on a Zeiss LSM800 using a 63x oil immersion objective and a least 4 different fields from a choice of 6 sections were randomly acquired. Laser irradiation at λ = 405, 488, 561 and 640 nm was used to detect HOECHST, Oregon green, Texas Red and Alexa fluor® 647, respectively. All the images were acquired with identical settings and processed using Zen 2.3 (blue edition) software to adjust the color balance. The offset for Texas Red channel was kept constant for the analysis of all different samples.

CD31 was chosen as a marker for liver sinusoidal endothelial cells (LSECs), as studies have confirmed that these cells possess CD31 intracellularly, thus giving positive immunohistochemical staining in LSECs in liver sections.[13-15]

Table S3. Liver accumulation of glycopolymers and gelatin *in vivo*. Fluorescence intensity percentage (FI%) of TR-gelatin and fluorescence intensity (FI) of S_{187} or G_{187} glycopolymers detected in mouse liver tissue homogenate following by sequential *i.p.* administration of Oregon Green-labeled glycopolymers and TR-gelatin.[a)] Samples were excited at λ_{ex} = 596 nm for Texas Red (λ_{em}= 615 nm) and at λ_{em}=495 nm for Oregon Green (λ_{em} = 535 nm) detection, respectively.

	Average FI % TR-gelatin per mg of tissue	Average FI Oregon Green-polymer per mg of tissue
PBS+TR-gelatin (positive control for S_{187}+TR-gelatin)	100 ± 10.9	0.10 ± 0.03
S_{187}+TR-gelatin	30.2 ± 22.9	0.7 ± 0.5
PBS+TR-gelatin (positive control for G_{187}+TR-gelatin)	100 ± 12.7	0.11 ± 0.03
G_{187}+TR-gelatin	138.3 ± 12.6	3.26 ± 1.16*[b)]

[a)]100 µL of PBS, or S_{187} or G_{187} solution in PBS at a polymer concentration of 400 µM were administered *i.p.* to mice. After 4 h, 100 µL of a 1.0 mg mL^{-1} TR-gelatin solution in PBS (treatment) were administered *i.p.*, and liver tissue was collected after further 2 h. [b)]Due to high fluorescence readings – in the liver several known receptors bind to galactose-containing ligands [16-18] - samples were diluted 5:1 vol:vol. The value shown in the table is obtained by multiplying the resulting average fluorescence reading by five: (FI/mg of tissue)*5. While variation of fluorescence may not always follow a perfectly linear trend, this value still provides an indication of higher uptake of galactose-containing polymer G_{187} when compared to its galactose 3-*O*-sulfated analogue S_{187}.

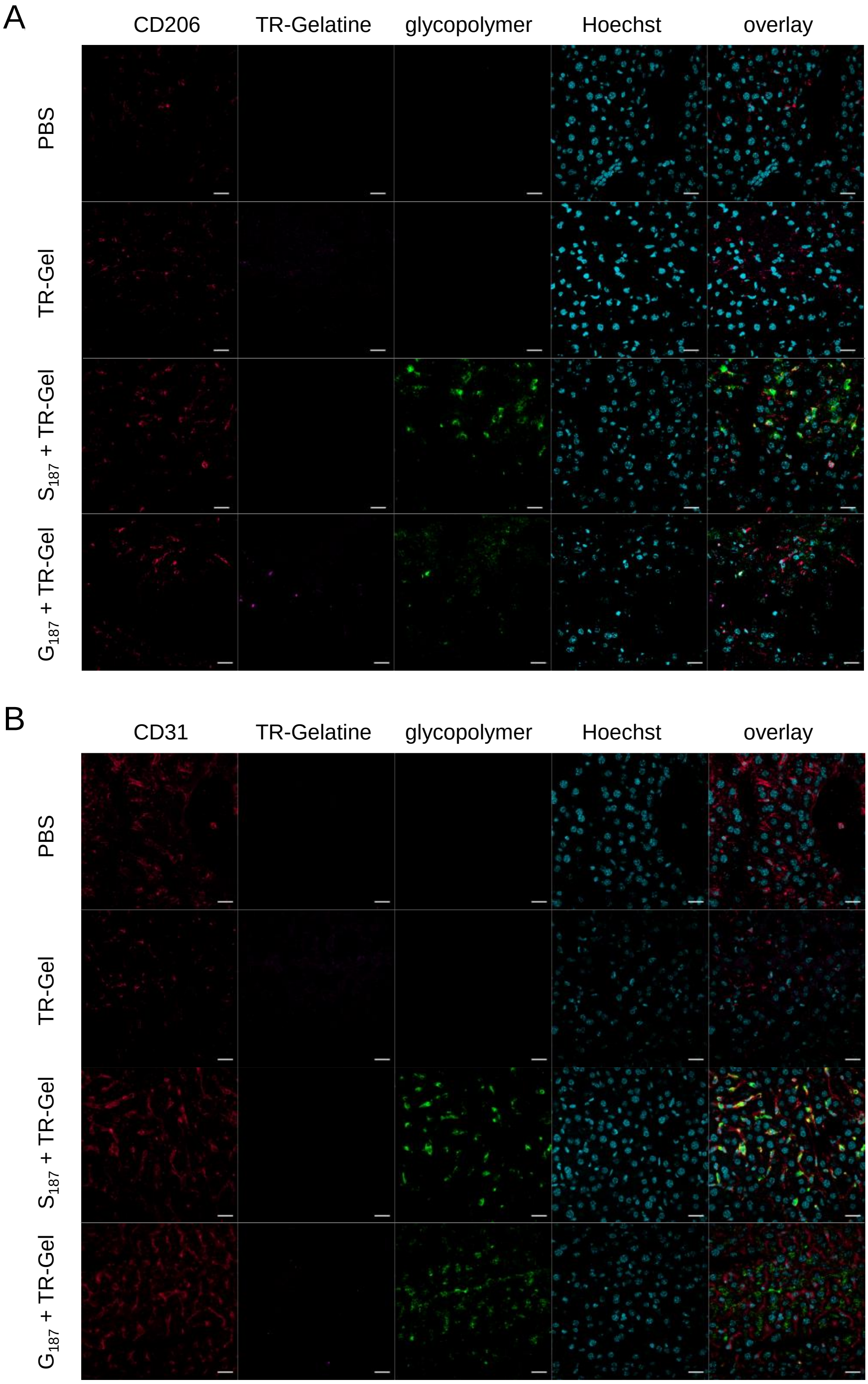

Figure S15. Representative two-dimensional confocal laser scanning images of liver sections of mice treated with S_{187}, G_{187} (both 100 µL of 400 µM solutions, polymer concentration, in PBS), or PBS solutions (*i.p.* administration, 4 h), followed by *i.p.* injection of 100 µL of Texas Red-tagged gelatin solution in PBS (56 mg kg^{-1}). Samples were imaged with Hoechst staining for nuclei (cyan), immunostaining for (A) CD206 or (B) CD31 receptor (red), TR-gelatin (magenta), and S_{187} or G_{187} glycopolymers (green) in mouse livers acquired with a 63x immersion oil objective and a magnification of 0.5x. Mice were treated with PBS only (PBS),

TR-gelatin only (TR-Gel) or S_{187} or G_{187} followed by TR-gelatin (S_{187} +TR-Gel, or G_{187} + TR-Gel) as described above. Scale Bar: 20 µm.

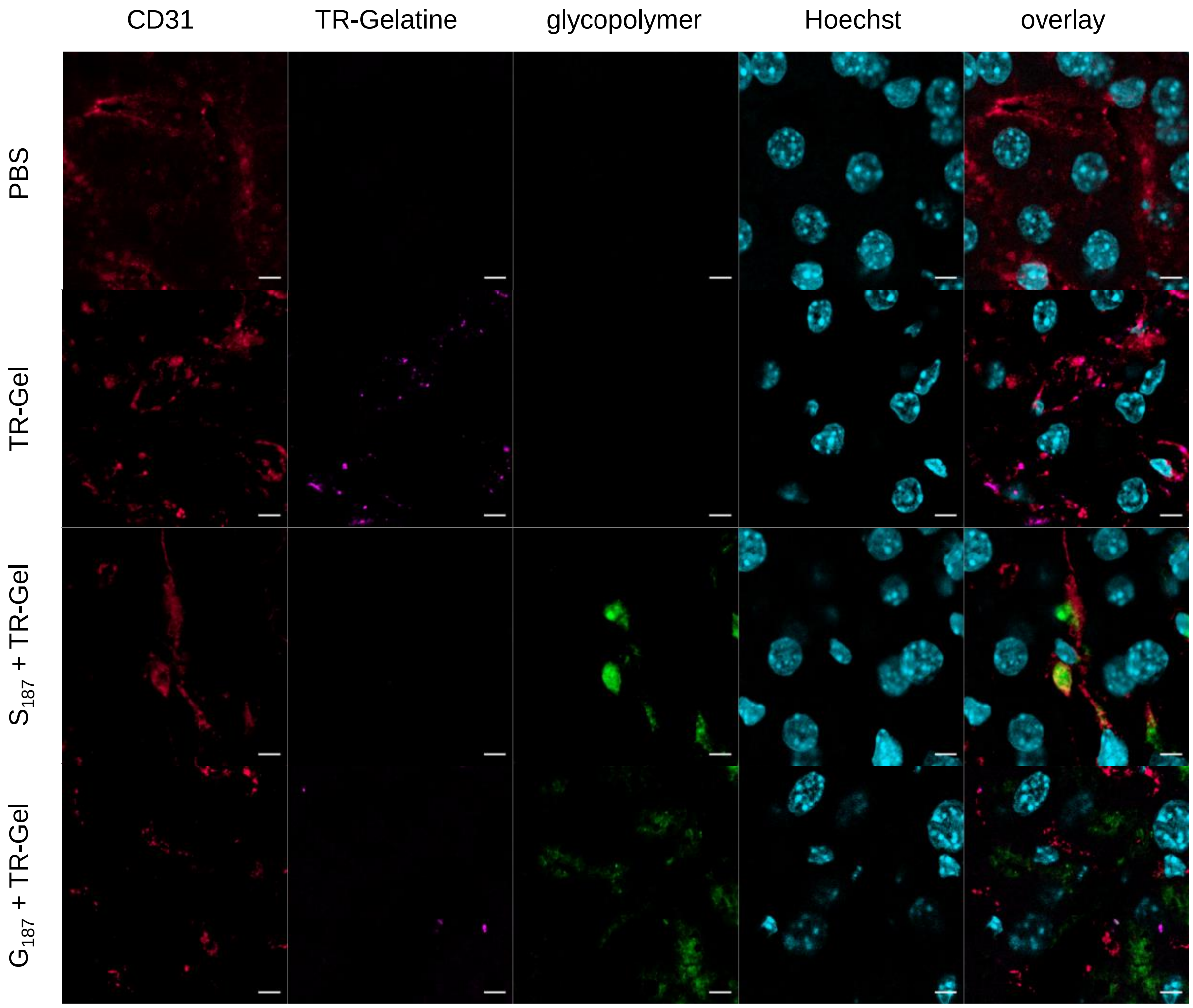

Figure S16. Representative two-dimensional confocal laser scanning images of liver sections of mice treated with S_{187}, G_{187} (both 100 µL of 400 µM solutions, polymer concentration, in PBS), or PBS solutions (*i.p.* administration, 4 h), followed by *i.p.* injection of 100 µL of TR-gelatin solution in PBS (56 mg kg^{-1}). Samples were imaged with Hoechst staining for nuclei (cyan), immunostaining for CD31 receptor (red), TR-gelatin (magenta), and S_{187} or G_{187} glycopolymers (green) in mouse livers acquired with a 63x immersion oil objective and a magnification of 2x. Mice were treated with PBS only (PBS), TR-gelatin only (TR-Gel), or S_{187} or G_{187} followed by TR-gelatin (S_{187} +TR-Gel, or G_{187} + TR-Gel) as described above. Scale Bar: 5 µm. Liver sections stained for CD206 are shown in Figure 7.

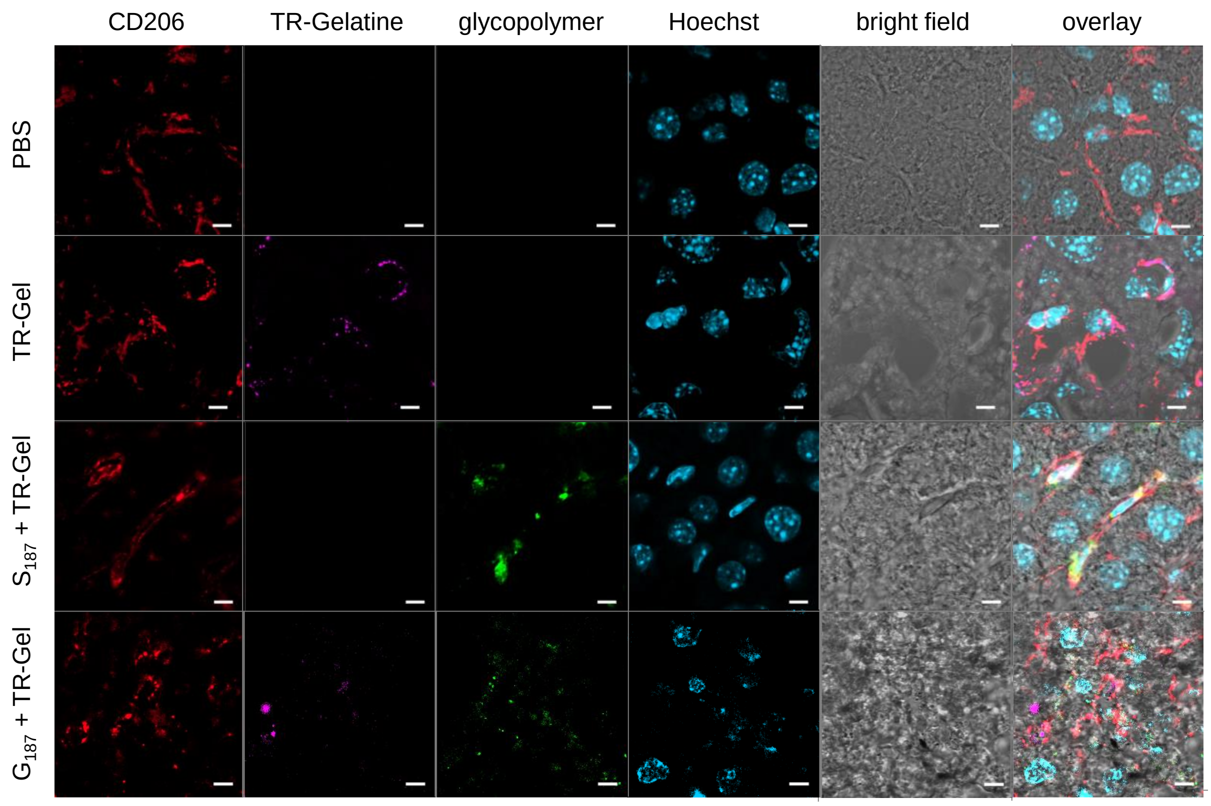

Figure S17. Representative two-dimensional confocal laser scanning and bright field images of liver sections of mice treated with S_{187} or G_{187} (both 100 µL of 400 µM solutions, polymer concentration, in PBS) or PBS solutions (*i.p.* administration, 4 hours), followed by *i.p.* injection of 100 µL of TR-gelatin solution in PBS (56 mg kg^{-1}). Samples were imaged with Hoechst staining for nuclei (cyan), immunostaining for CD206 receptor (red), TR-gelatin (magenta), Bright Field (BF, gray), and S_{187} glycopolymers (green) in mouse livers acquired with a 63x immersion oil objective and a magnification of 2x. Mice were treated with PBS only (PBS) or S_{187} or G_{187} followed by TR-gelatin (S_{187} +TR-Gel) as described above. Scale Bar: 5 µm.

1.8.3 Mouse plasma collection and analysis post-treatment with Texas Red-tagged gelatin and glycopolymers

Blood samples (0.7-1 mL) were divided into 2 aliquots, diluted with 1 mL of PBS each and centrifuged at 1,100 rpm for 10 min at 4°C. Supernatants containing plasma fraction were collected and analyzed by spectrofluorometer to assess gelatin (Texas Red) and glycopolymer (Oregon Green) content, recording the emission spectra from 610 to 700 nm (λ_{ex} = 596 nm) for TR-gelatin, and from 515 to 700 nm (λ_{ex} = 495 nm) for Oregon Green-tagged glycopolymers G_{187} and S_{187}.

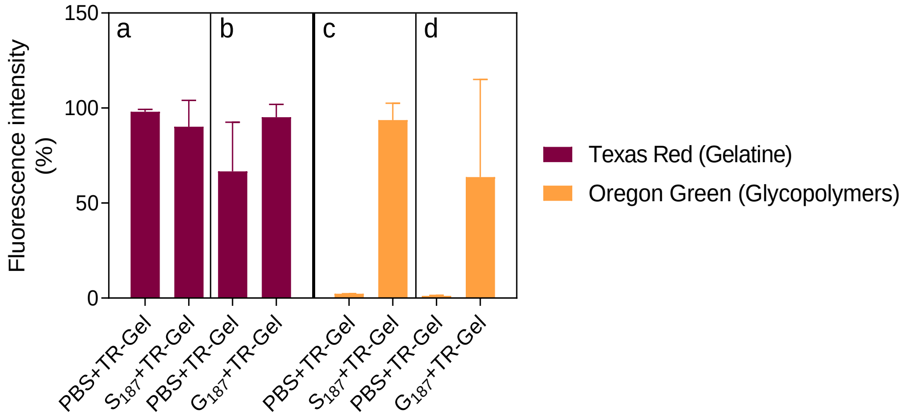

Figure S18. Mouse plasma analysis post-treatment with TR-gelatin and glycopolymers. Analysis of plasma samples withdrawn from mice 4 h after *i.p.* injection of S_{187} or G_{187} glycopolymers (100 µL of 400 µM polymer solution in PBS) or PBS (100 µL), followed by treatment for further 2 h with *i.p.* injection of 100 µL of a TR-gelatin solution in PBS (1 mg mL^{-1}). (a) Comparison of TR-gelatin plasma levels in mice pre-treated with PBS or S_{187}; (b) comparison of TR-gelatin plasma levels for mice pre-treated with PBS or G_{187}; (c) comparison of glycopolymer-Oregon Green levels for mice pre-treated with PBS or S_{187}; (d) comparison of glycopolymer-Oregon Green levels for mice pre-treated with PBS or G_{187}. For c and d, samples were diluted 1:2 with PBS before analysis as glycopolymer samples were out of range for Oregon Green detection.
Data are reported as percentage mean ± s.d. of the fluorescence intensity (the higher value of each group of experiments was set as 100%), N=2 independent biological samples with two technical replicates.

1.9 Statistical analysis

Statistical analysis was performed using Prism software 7.04 (GraphPad). Normality was assessed by Shapiro-Wilk normality test. Statistical significance for two group comparison was calculated by two-tailed Student's t-test while for comparison with more than two groups ordinary one-way ANOVA was used. Data are presented as mean values and s.d. Two groups were considered to be significantly different if $P<0.05$. For the *in vivo* study, GPower 3.1 was used to perform calculations on sample size. The minimal significance (α) and statistical power ($1 - \beta$) were set at 0.05 and 0.80 respectively. Calculations were performed for two groups.

2 Additional NMR spectra.

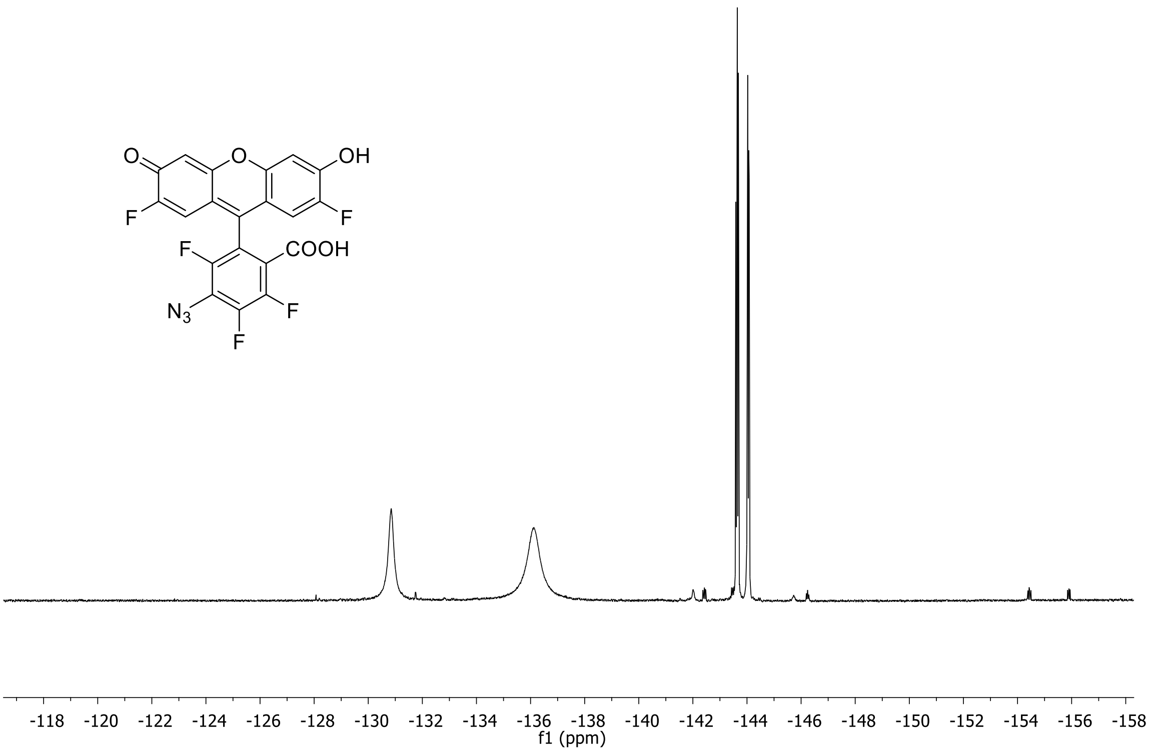

Figure S19. ^{19}F NMR spectrum of 6-azido-2,4,5,7,7'-pentafluorofluorescein (Oregon Green azide) in CD_3OD.

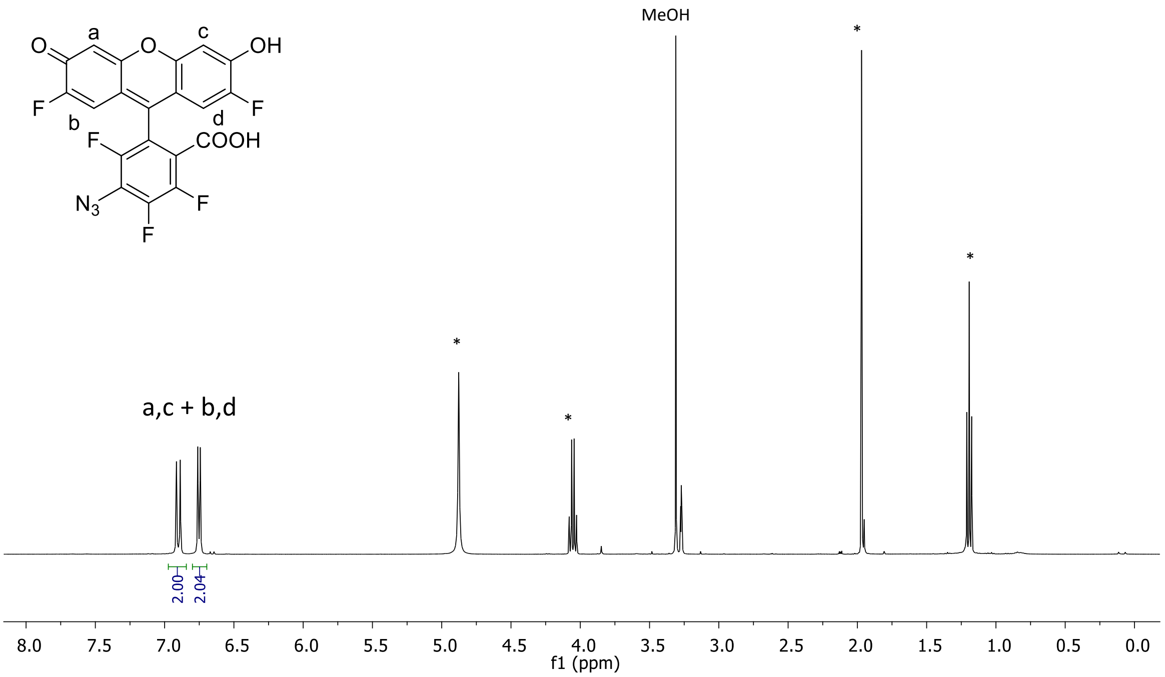

Figure S20. ^{1}H NMR spectrum of 6-azido-2,4,5,7,7'-pentafluorofluorescein (Oregon Green azide) in CD_3OD. * Ethyl acetate.

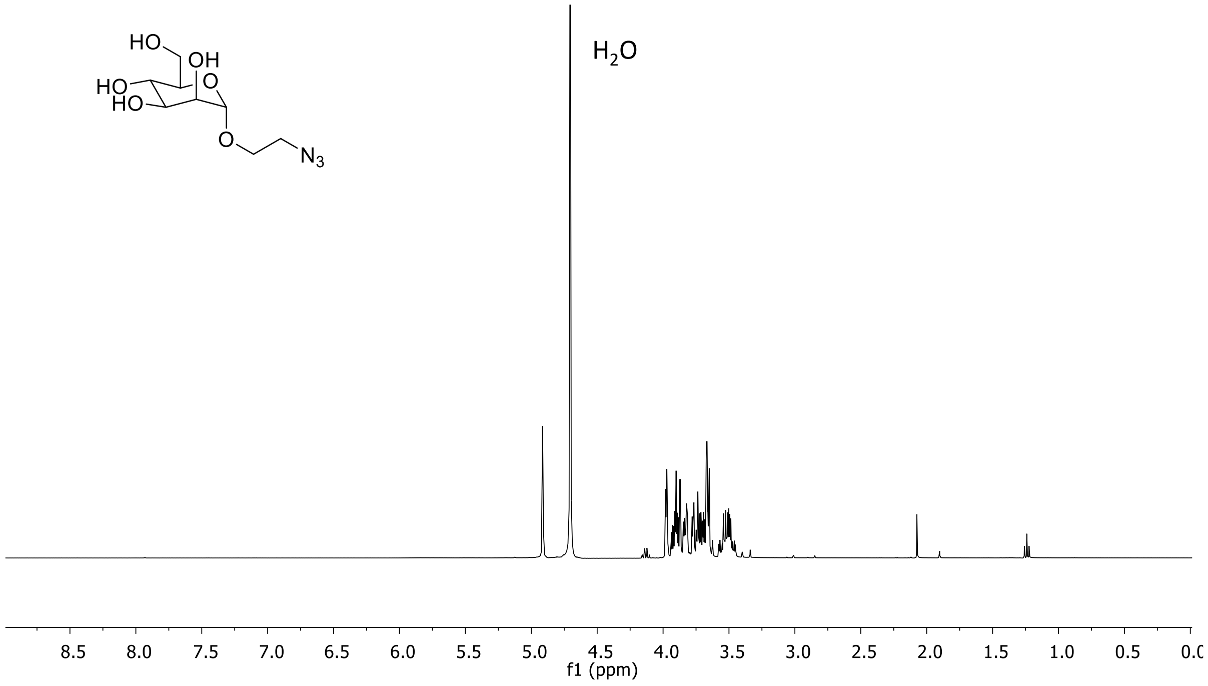

Figure S21. ^{1}H NMR spectrum of 2'-azidoethyl-*O*-*α*-D-mannopyranoside in D_2O.

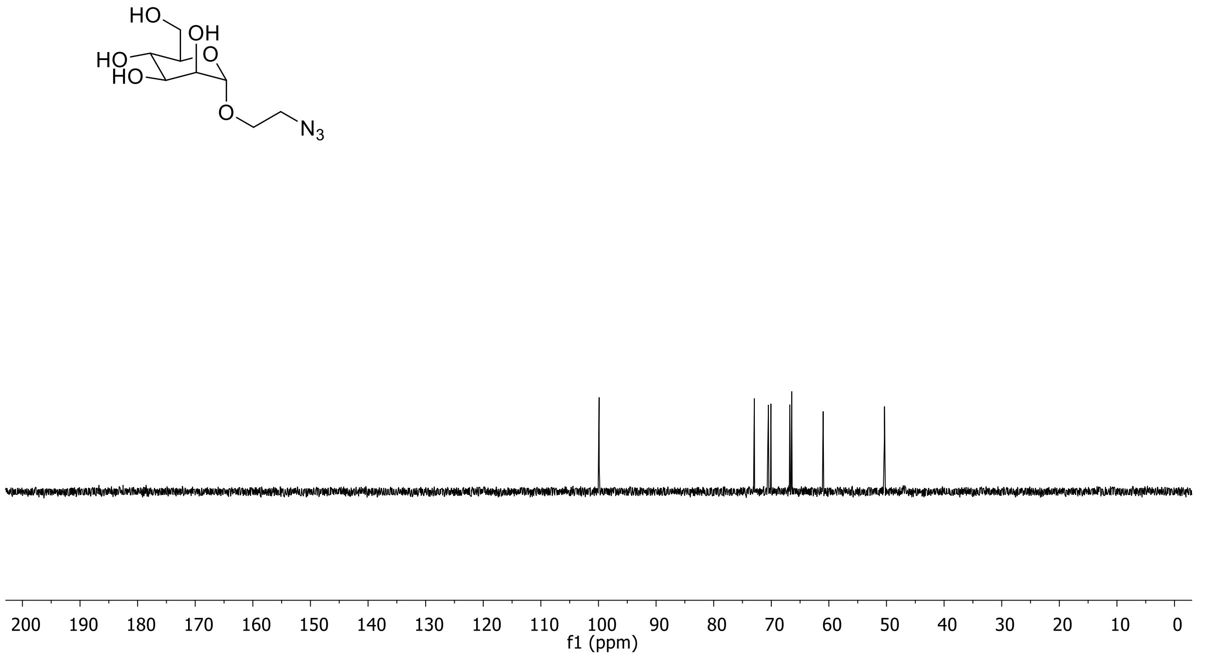

Figure S22. ^{13}C NMR spectrum of 2'-azidoethyl-*O*-*α*-D-mannopyranoside in D_2O.

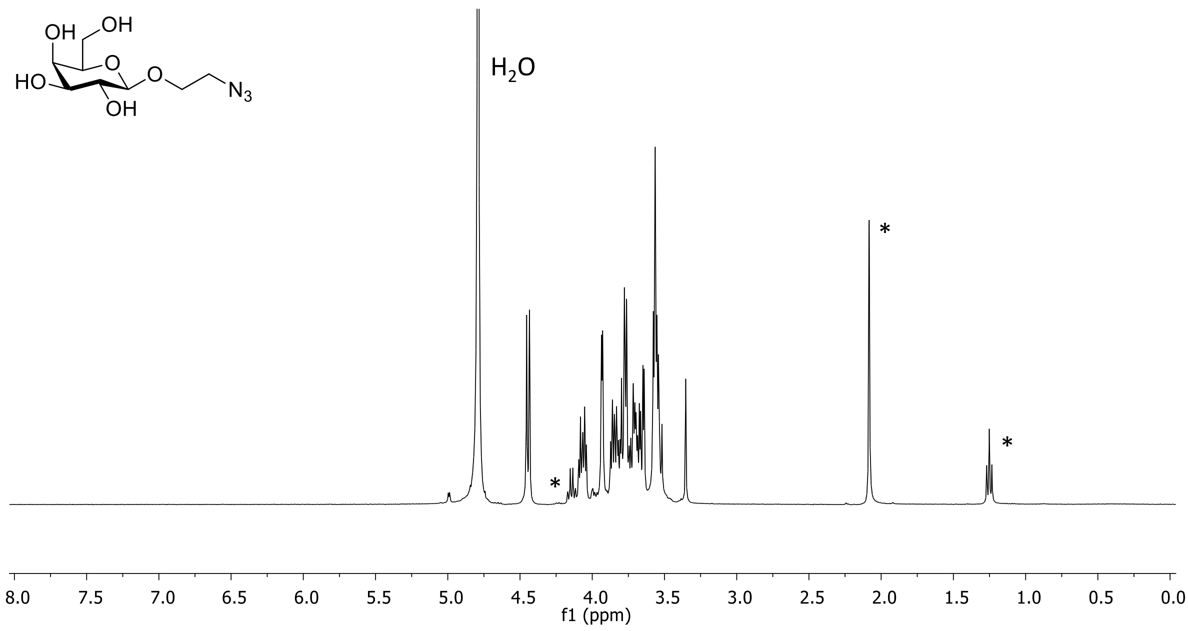

Figure S23. ^{1}H NMR spectrum of 2'-azidoethyl-*O*-*β*-D-galactopyranoside in D_2O (* ethyl acetate).

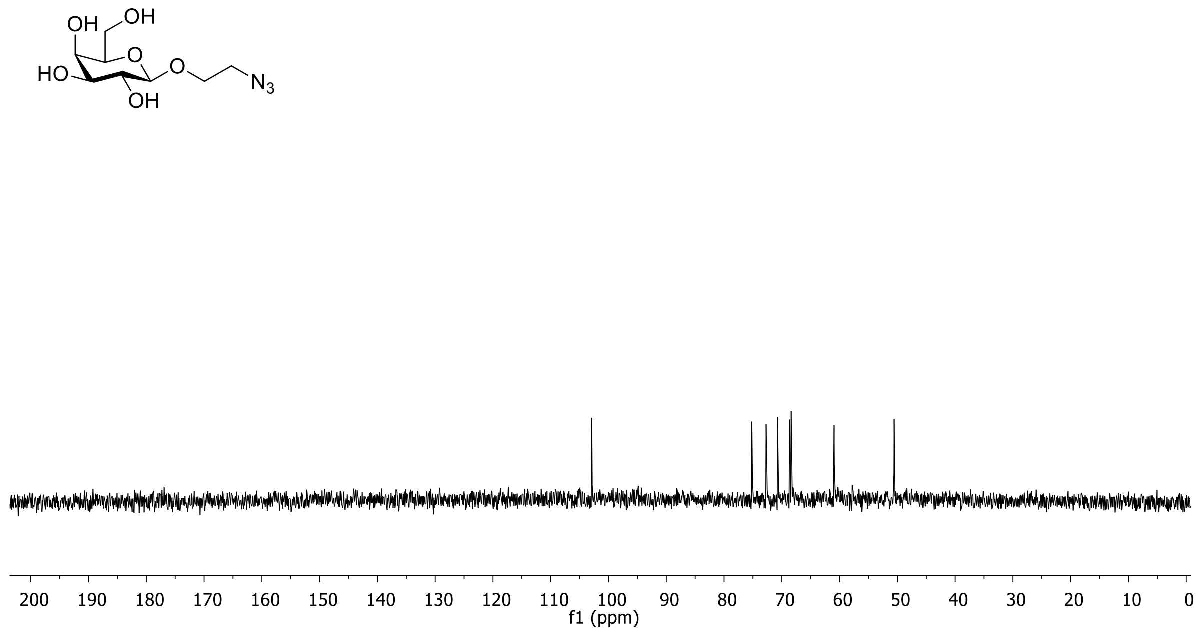

Figure S24. ^{13}C NMR spectrum of 2'-azidoethyl-*O*-*β*-D-galactopyranoside in D_2O.

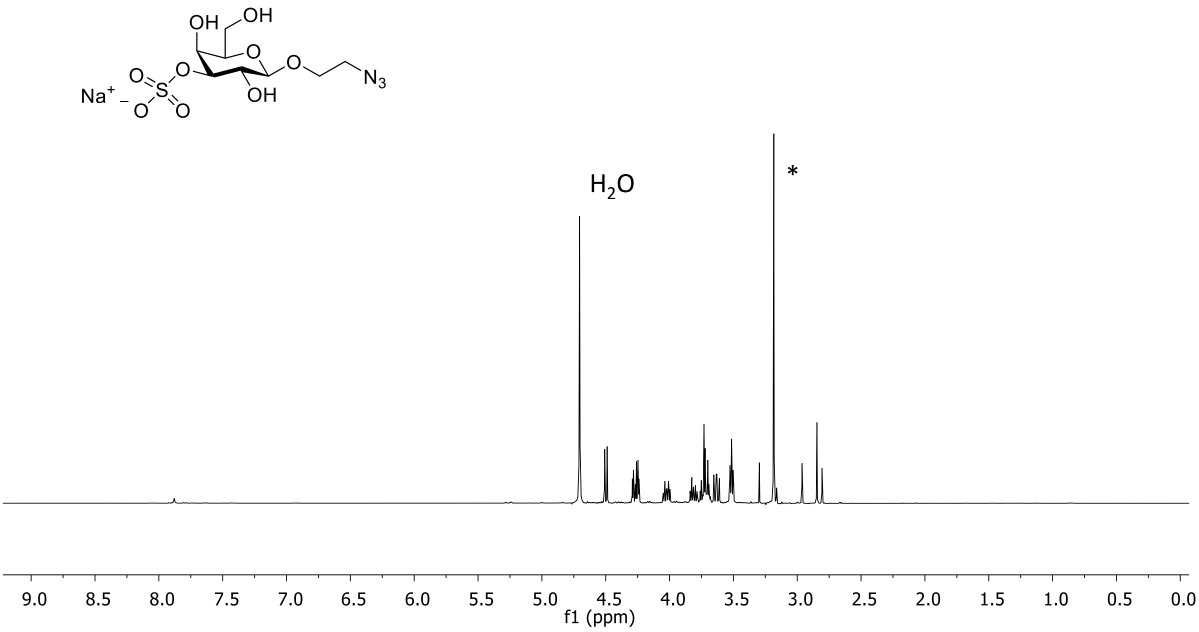

Figure S25. ^{1}H NMR spectrum of 3-*O*-sulfo-2'-azidoethyl-*O*-*β*-D-galactopyranoside in D_2O (* methanol).

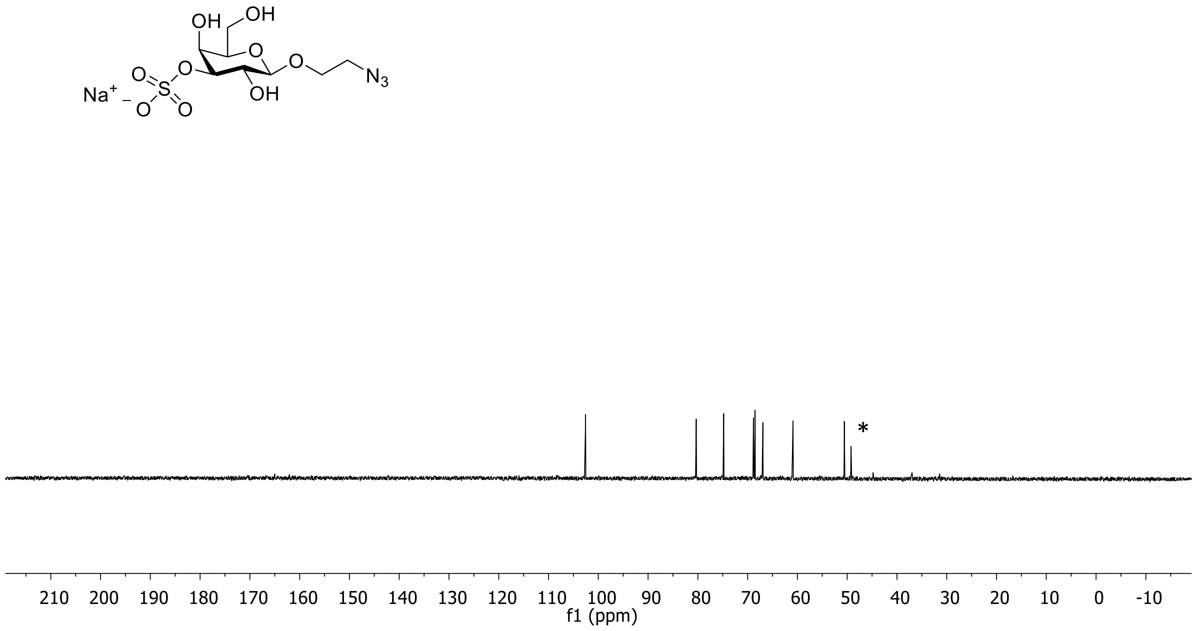

Figure S26. ^{13}C NMR spectrum of 3-*O*-sulfo-2'-azidoethyl-*O*-*β*-D-galactopyranoside in D_2O (* methanol).

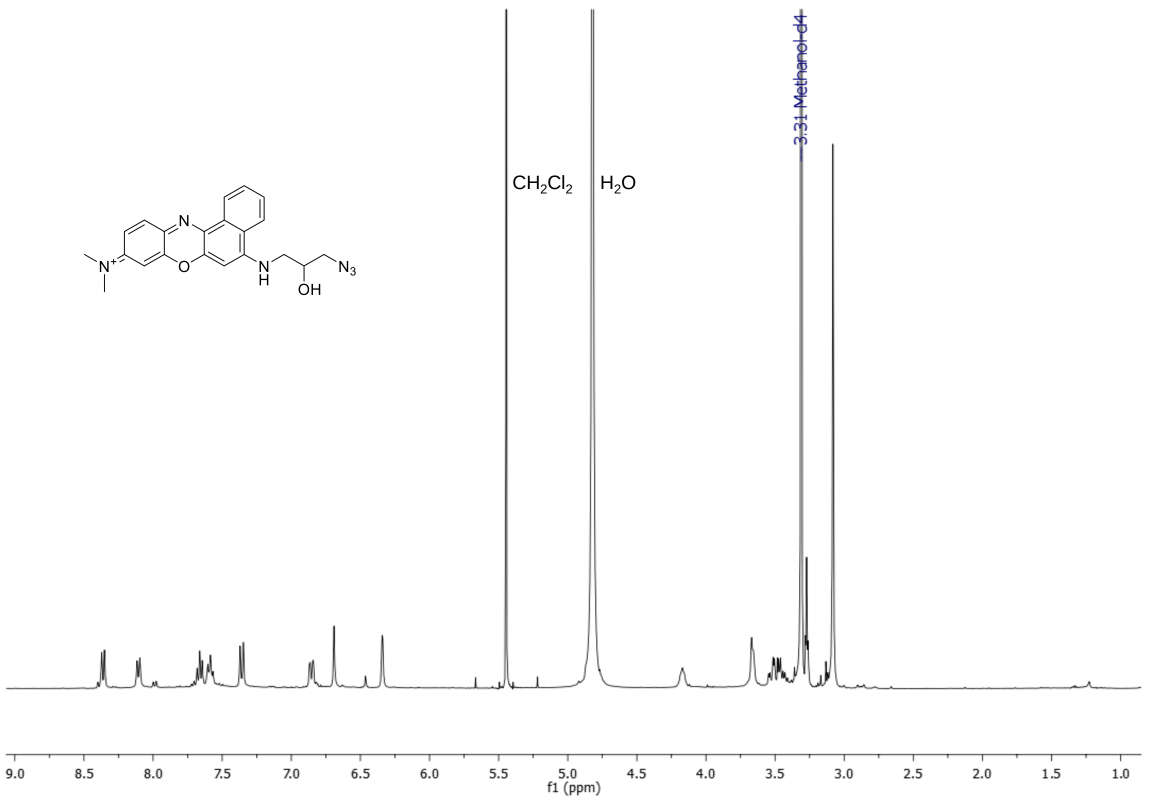

Figure S27. ^{1}H NMR spectrum of *N*-(5-((3-azido-2-hydroxypropyl)amino)-9H-benzo[a]phenoxazin-9-ylidene)-*N*-methylmethanaminium (Nile Blue azide) in CD_3OD.

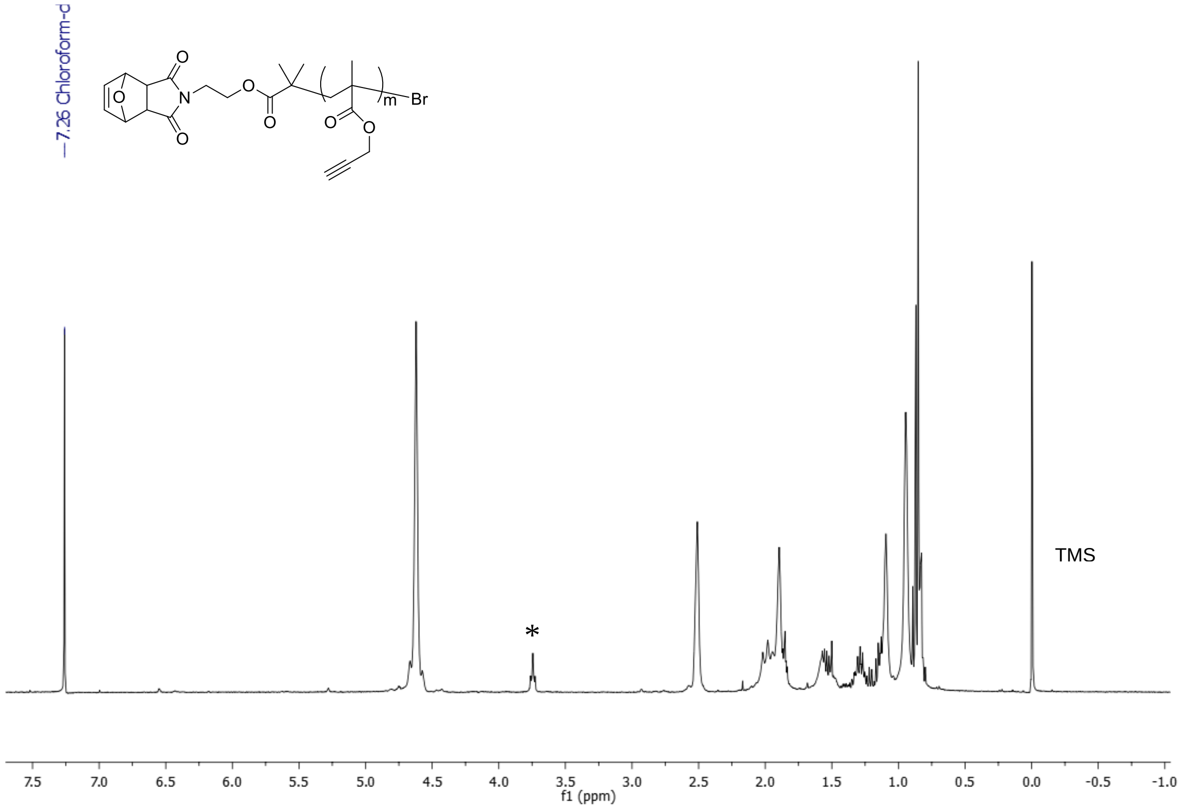

Figure S28. ^{1}H NMR analysis of clickable poly(propargyl methacrylate)s: spectrum of PMA_{187} in $CDCl_3$ (* = THF).

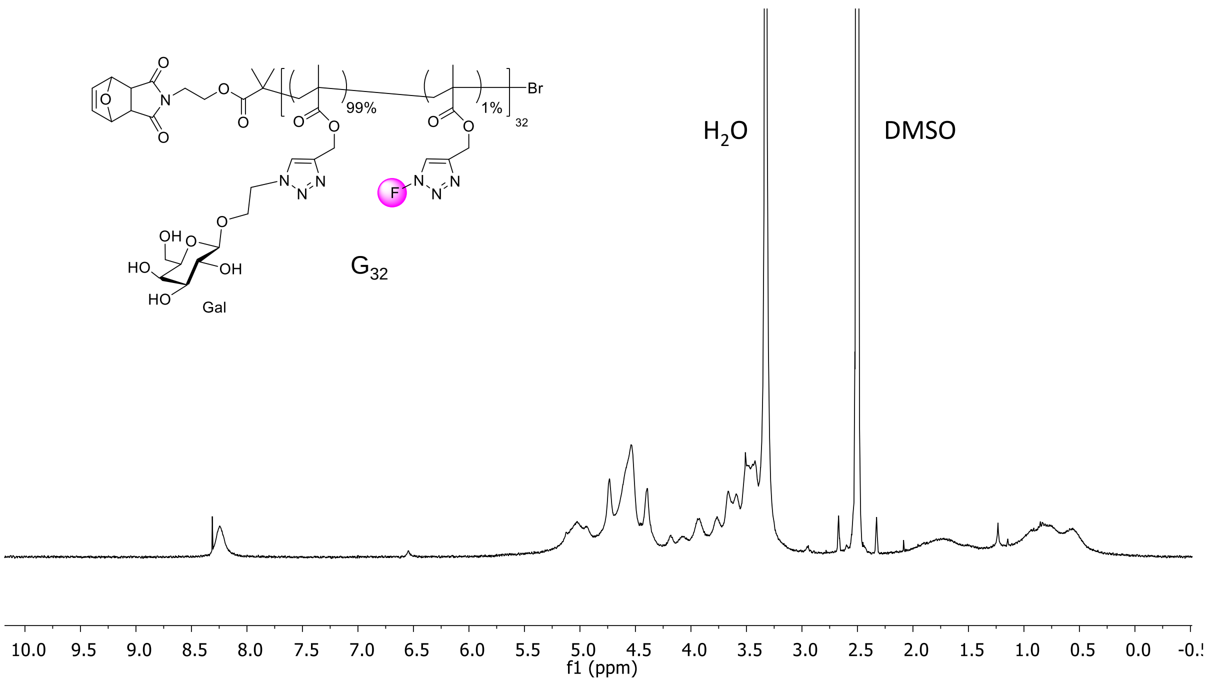

Figure S29. ^{1}H NMR spectrum of G_{32} in DMSO-d_6.

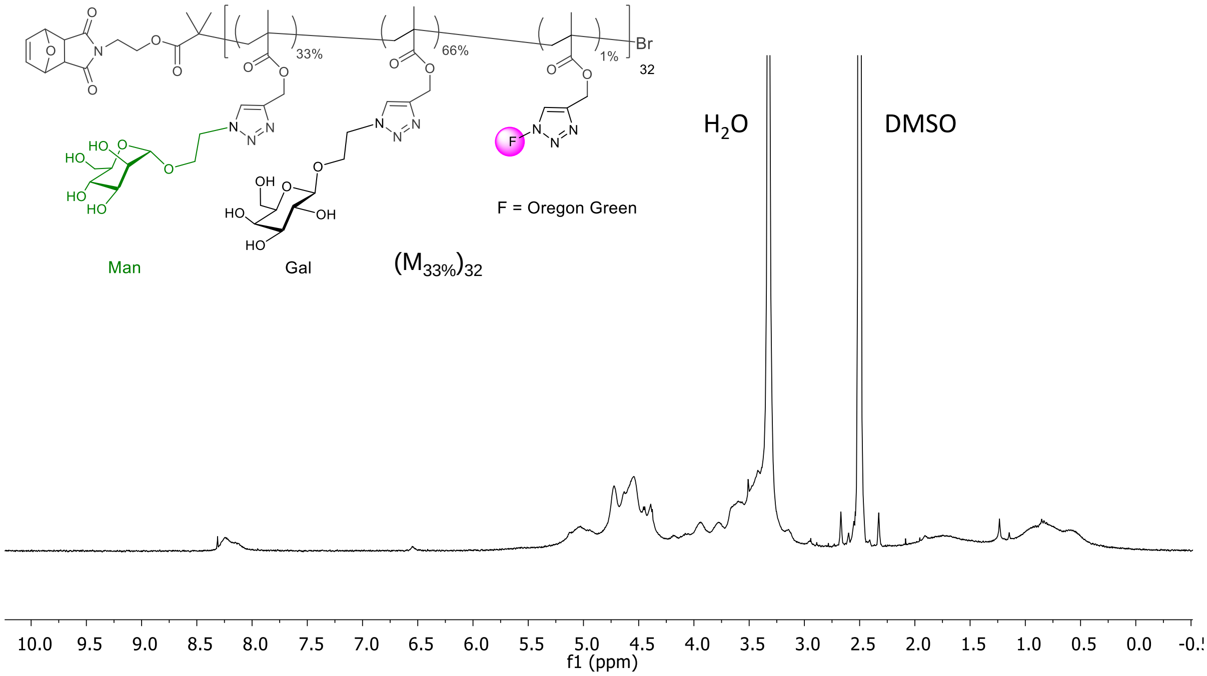

Figure S30. ^{1}H NMR spectrum of $(M_{33\%})_{32}$ in DMSO-d_6.

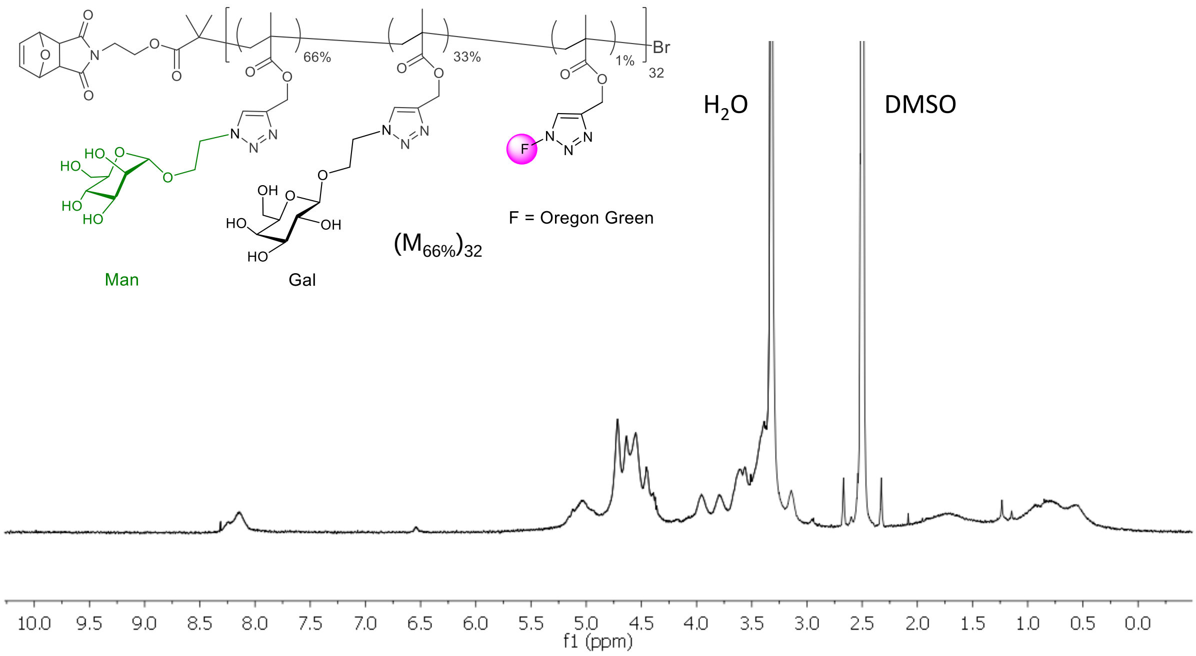

Figure S31. ^{1}H NMR spectrum of $(M_{66\%})_{32}$ in DMSO-d_6.

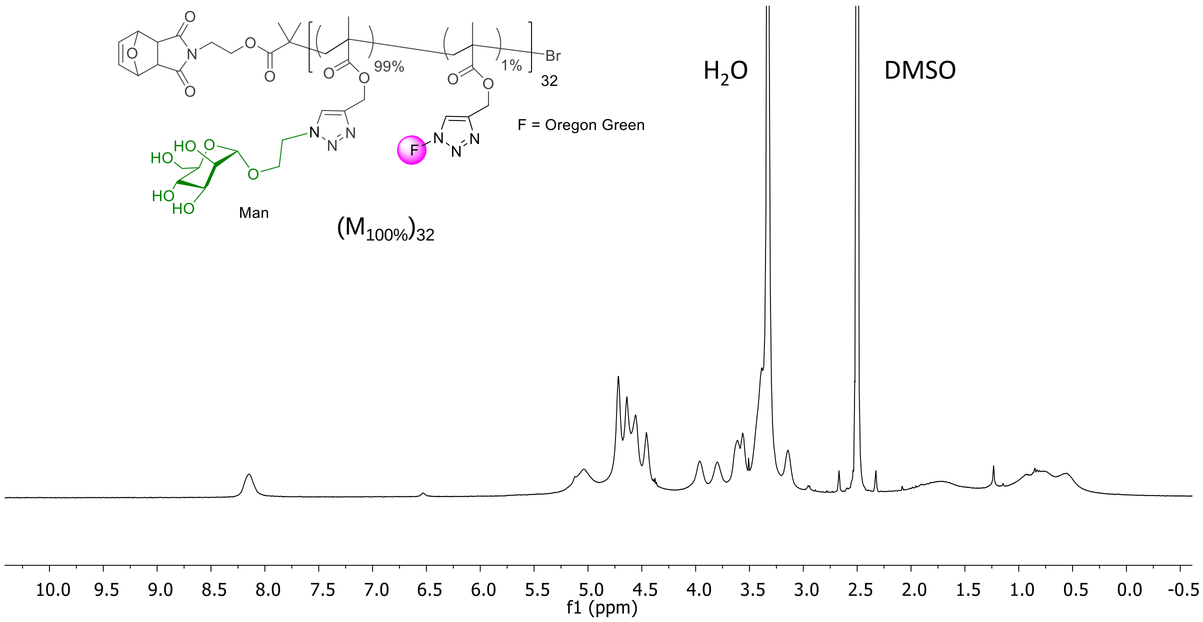

Figure S32. ^{1}H NMR spectrum of $(M_{100\%})_{32}$ (= M_{32}) in DMSO-d_6.

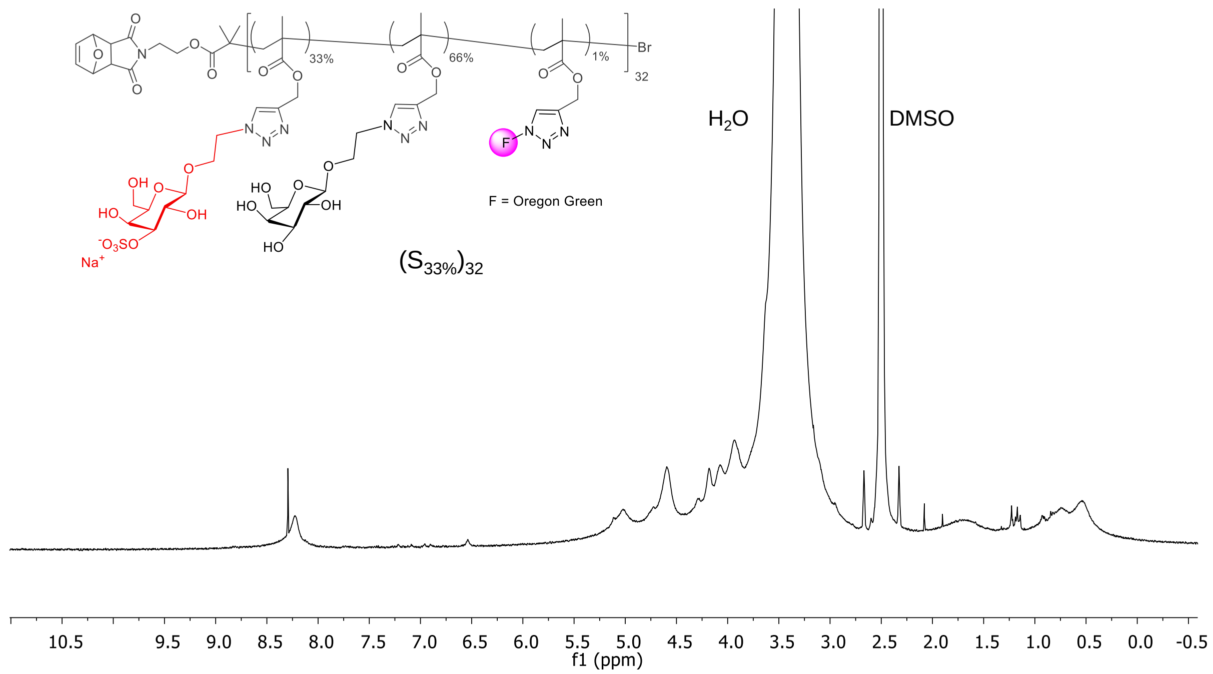

Figure S33. ^{1}H NMR spectrum of $(S_{33\%})_{32}$ in DMSO-d_6.

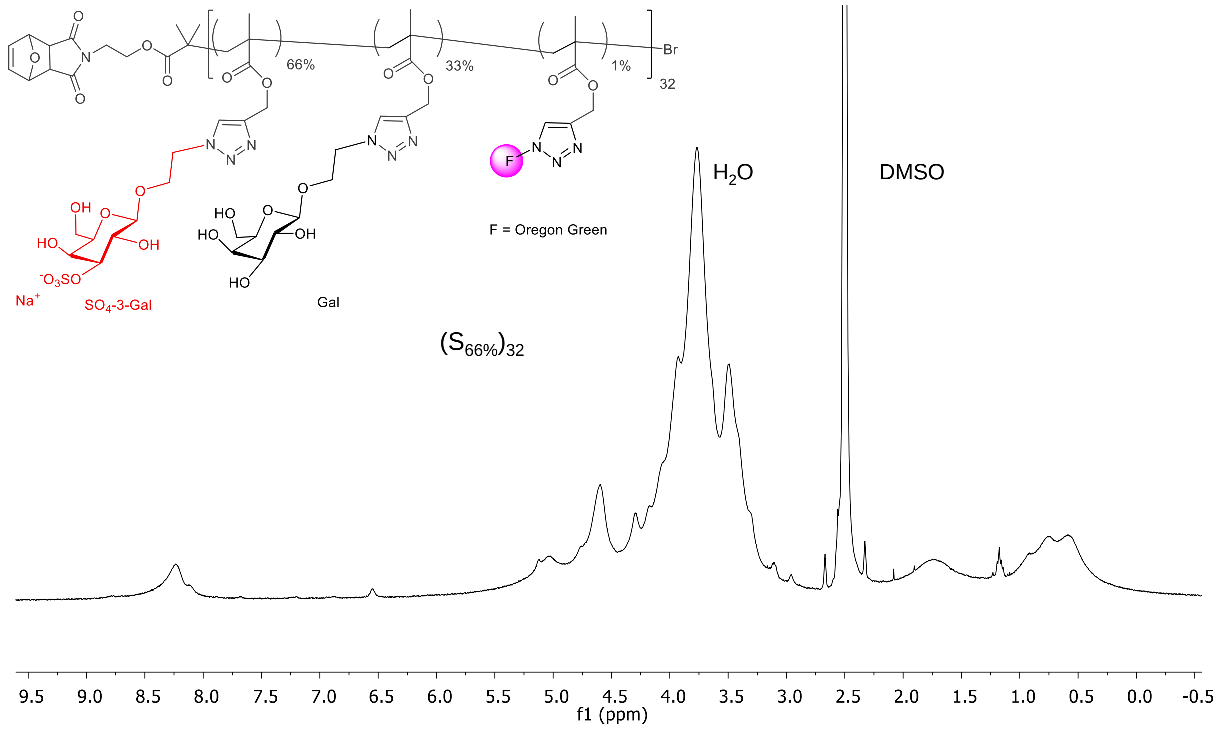

Figure S34. ^{1}H NMR spectrum of $(S_{66\%})_{32}$ in DMSO-d_6.

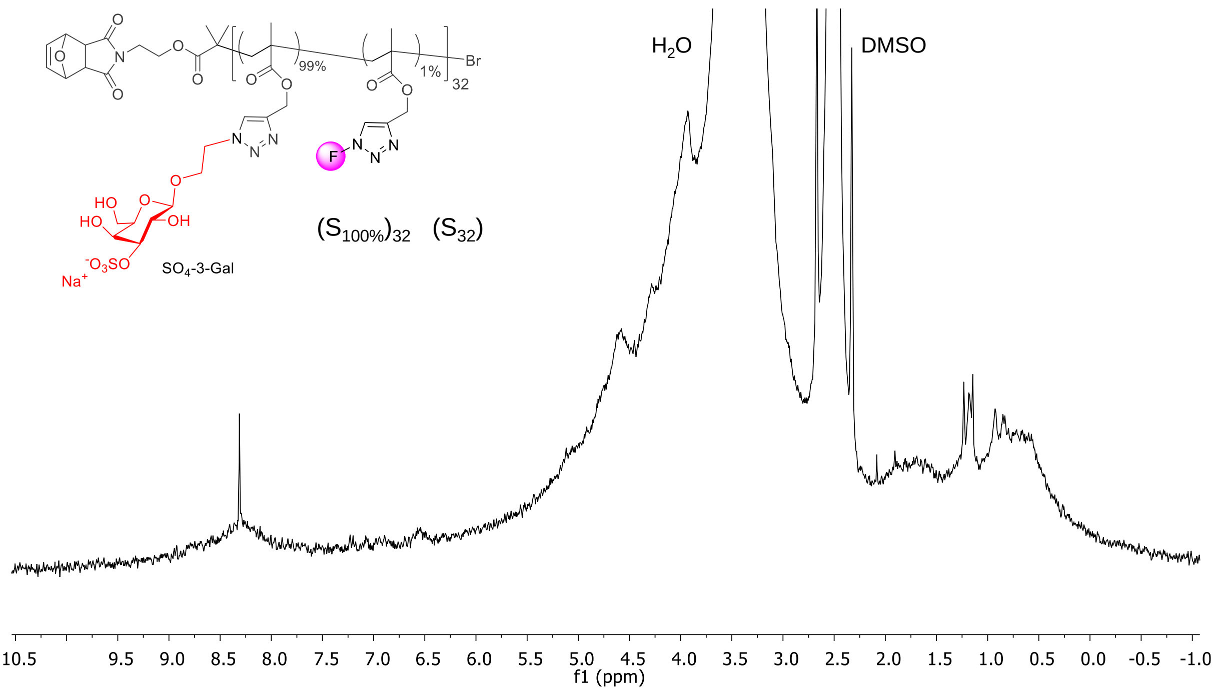

Figure S35. ^{1}H NMR spectrum of $(S_{100\%})_{32}$ (= S_{32}) in DMSO-d_6.

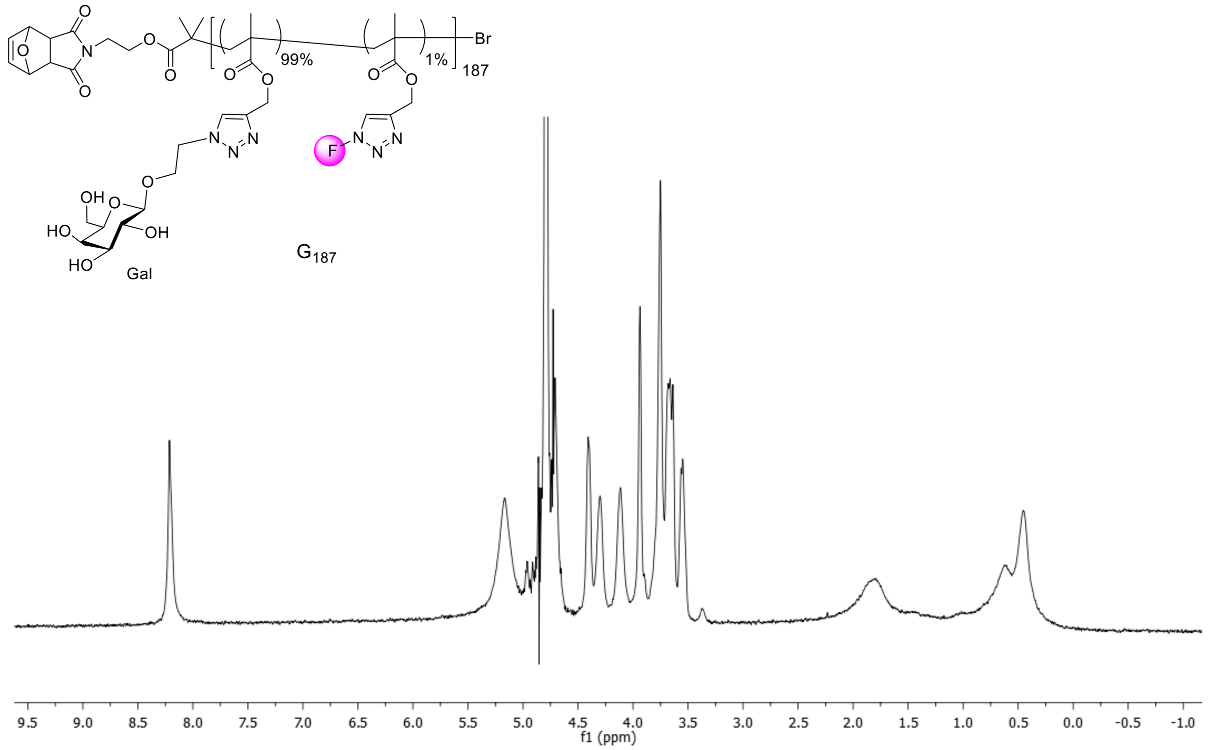

Figure S36. ^{1}H NMR spectrum of G_{187} in D_2O.

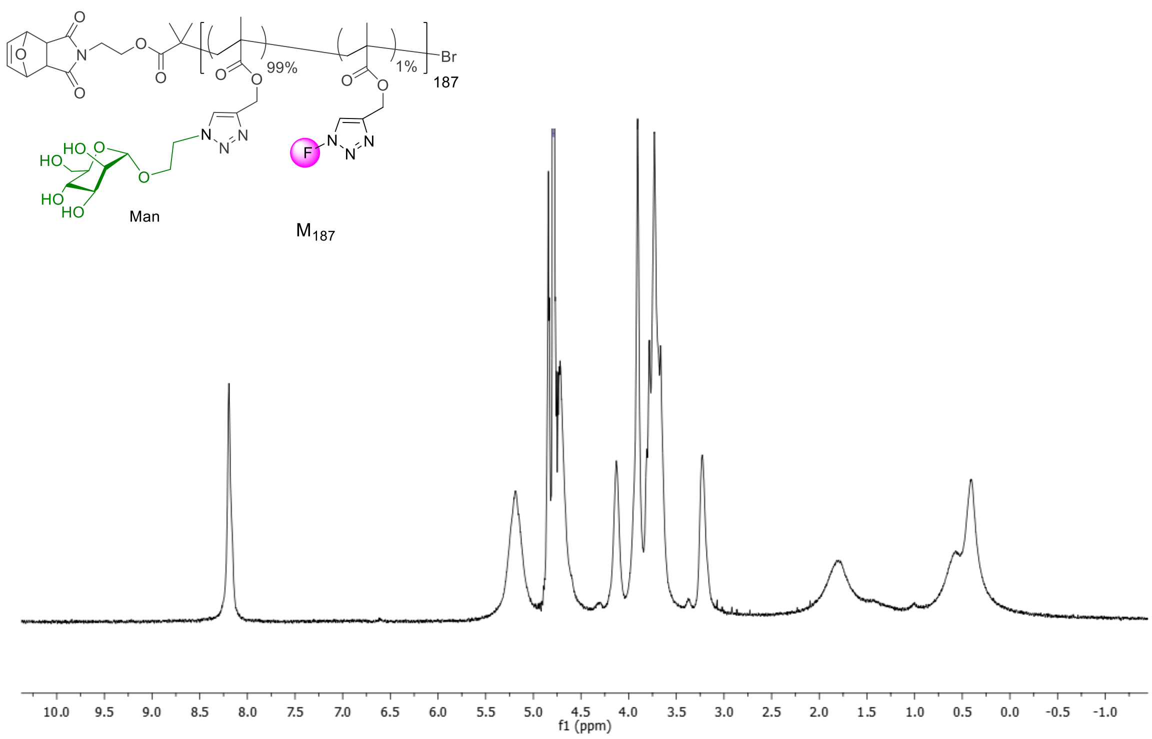

Figure S37. ^{1}H NMR spectrum of M_{187} in D_2O.

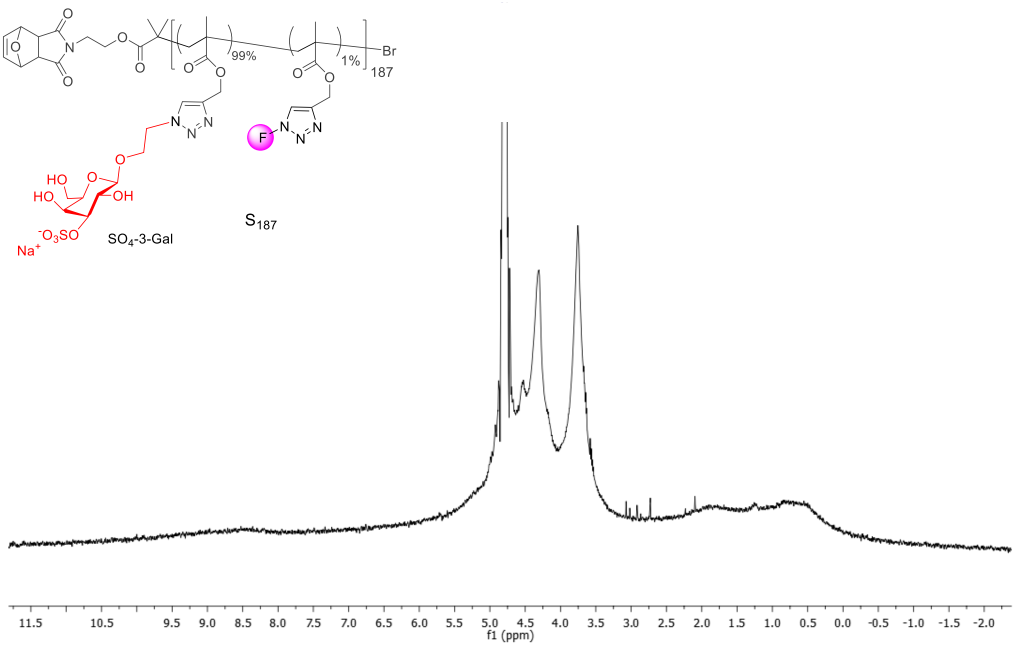

Figure S38. ^{1}H NMR spectrum of S_{187} in D_2O. Peak broadening is observed for all Gal sulfate glycopolymers, and increases at higher SO_4-3-Gal content.

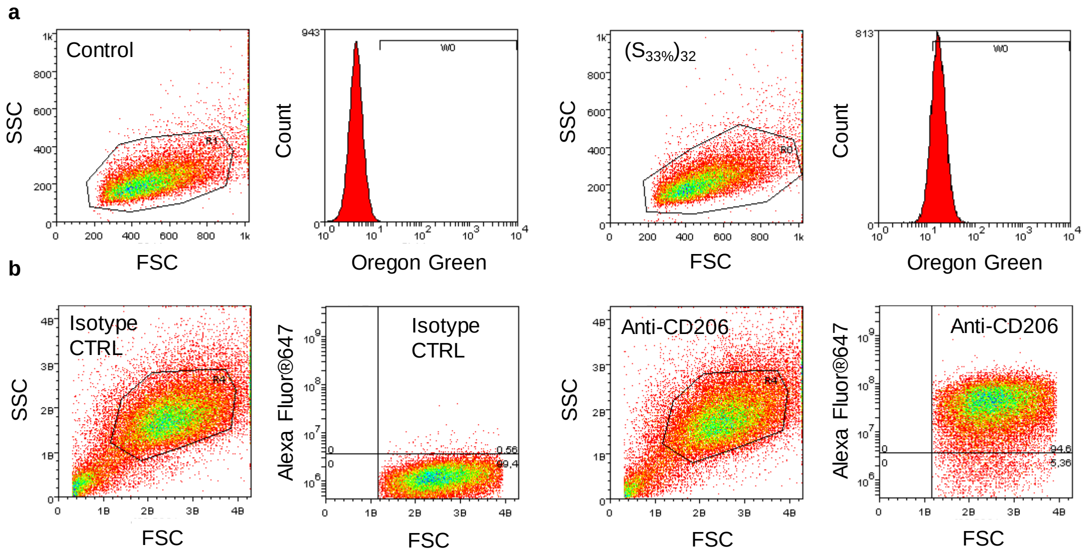

Figure S39. Example of flow cytometry gating strategy and analysis. a. CD206$^+$-CHO: cells for analysis were selected based on FSC/SSC characteristics to exclude debris (94.25% of the cells in the gate, gate R1). The cells within the gate were plotted in a histogram plot and the mean fluorescence intensity on FL1 was determined (λ = 525 nm, Oregon Green). Examples of untreated cells and cells incubated with $(S_{33\%})_{32}$ are provided. b. WT macrophages: cells were selected based on FSC/SSC characteristics to exclude debris (71% of the cells in the gate, gate R4). CD206$^+$ cells were then gated (Alexa Fluor® 647 anti-CD206 antibody, 94.6% positive cells) and compared with control sample (Alexa Fluor® 647 isotype control IgG2a, 0.56% positive cells).

References

(1) Magennis, E. P.; Francini, N.; Mastrotto, F.; Catania, R.; Redhead, M.; Fernandez-Trillo, F.; Bradshaw, D.; Churchley, D.; Winzer, K.; Alexander, C.; et al. Polymers for binding of the gram-positive oral pathogen Streptococcus mutans. *PLOS ONE* **2017**, *12* (7), e0180087.

(2) Geng, J.; Mantovani, G.; Tao, L.; Nicolas, J.; Chen, G.; Wallis, R.; Mitchell, D. A.; Johnson, B. R. G.; Evans, S. D.; Haddleton, D. M. Site-Directed Conjugation of “Clicked” Glycopolymers To Form Glycoprotein Mimics: Binding to Mammalian Lectin and Induction of Immunological Function. *Journal of the American Chemical Society* **2007**, *129* (49), 15156-15163.

(3) Martinez-Pomares, L.; Reid, D. M.; Brown, G. D.; Taylor, P. R.; Stillion, R. J.; Linehan, S. A.; Zamze, S.; Gordon, S.; Wong, S. Y. Analysis of mannose receptor regulation by IL-4, IL-10, and proteolytic processing using novel monoclonal antibodies. *Journal of leukocyte biology* **2003**, *73* (5), 604-613.

(4) Zamze, S.; Martinez-Pomares, L.; Jones, H.; Taylor, P. R.; Stillion, R. J.; Gordon, S.; Wong, S. Y. Recognition of bacterial capsular polysaccharides and lipopolysaccharides by the macrophage mannose receptor. *The Journal of biological chemistry* **2002**, *277* (44), 41613-41623.

(5) Yu, P.; Wang, Y.; Chin, R. K.; Martinez-Pomares, L.; Gordon, S.; Kosco-Vibois, M. H.; Cyster, J.; Fu, Y.-X. B Cells Control the Migration of a Subset of Dendritic Cells into B Cell Follicles Via CXC Chemokine Ligand 13 in a Lymphotoxin-Dependent Fashion. *Journal of Immunology* **2002**, *168* (10), 5117-5123.

(6) Manning, D. D.; Hu, X.; Beck, P.; Kiessling, L. L. Synthesis of Sulfated Neoglycopolymers: Selective P-Selectin Inhibitors. *J. Am. Chem. Soc.* **1997**, *119* (13), 3161-3162.

(7) Uzawa, H.; Minoura, N.; Toba, T.; Nishida, Y.; Kobayashi, K.; Hiratani, K. Convenient synthetic approach towards regioselectively sulfated sugars using limpet and abalone sulfatase-catalyzed desulfation. *Chemical Communications* **1998**, (21), 2311-2312, 10.1039/A805830J.

(8) Kele, P.; Li, X.; Link, M.; Nagy, K.; Herner, A.; Lorincz, K.; Beni, S.; Wolfbeis, O. S. Clickable fluorophores for biological labeling-with or without copper. *Organic & Biomolecular Chemistry* **2009**, *7* (17), 3486-3490, 10.1039/B907741C.

(9) Ladmiral, V.; Mantovani, G.; Clarkson, G. J.; Cauet, S.; Irwin, J. L.; Haddleton, D. M. Synthesis of Neoglycopolymers by a Combination of "Click Chemistry" and Living Radical Polymerization. *J. Am. Chem. Soc.* **2006**, *128*, 4823-4830, 10.1021/ja058364k.

(10) Hummert, E.; Henniges, U.; Potthast, A. Fluorescence labeling of gelatin and methylcellulose: monitoring their penetration behavior into paper. *Cellulose* **2013**, *20* (2), 919-931.

(11) Martinez-Pomares, L.; Wienke, D.; Stillion, R.; McKenzie, E. J.; Arnold, J. N.; Harris, J.; McGreal, E.; Sim, R. B.; Isacke, C. M.; Gordon, S. Carbohydrate-independent recognition of collagens by the macrophage mannose receptor. *Eur. J. Immunol.* **2006**, *36* (5), 1074-1082.

(12) Martínez-Pomares, L.; Mahoney, J. A.; Káposzta, R.; Linehan, S. A.; Stahl, P. D.; Gordon, S. A functional soluble form of the murine mannose receptor is produced by macrophages in vitro and is present in mouse serum. *The Journal of biological chemistry* **1998**, *273* (36), 23376-23380.

(13) Poisson, J.; Lemoinne, S.; Boulanger, C.; Durand, F.; Moreau, R.; Valla, D.; Rautou, P.-E. Liver sinusoidal endothelial cells: Physiology and role in liver diseases. *Journal of Hepatology* **2017**, *66* (1), 212-227.

(14) Elvevold, K.; Smedsrød, B.; Martinez, I. The liver sinusoidal endothelial cell: a cell type of controversial and confusing identity. *American Journal of Physiology: Gastrointestinal and Liver Physiology* **2008**, *294* (2), G391-G400.

(15) DeLeve, L. D.; Wang, X.; Hu, L.; McCuskey, M. K.; McCuskey, R. S. Rat liver sinusoidal endothelial cell phenotype is maintained by paracrine and autocrine regulation. *American Journal of Physiology: Gastrointestinal and Liver Physiology* **2004**, *287* (4), G757-G763.

(16) Ashwell, G.; Harford, J. Carbohydrate-Specific Receptors of the Liver. *Annual Review of Biochemistry* **1982**, *51* (1), 531-554.

(17) Roos, P. H.; Kolb-Bachofen, V.; Schlepper-Schäfer, J.; Monsigny, M.; Stockert, R. J.; Kolb, H. Two galactose-specific receptors in the liver with different function. *FEBS Letters* **1983**, *157* (2), 253-256.

(18) Roggenbuck, D.; Mytilinaiou, M. G.; Lapin, S. V.; Reinhold, D.; Conrad, K. Asialoglycoprotein receptor (ASGPR): a peculiar target of liver-specific autoimmunity. *Auto-immunity highlights* **2012**, *3* (3), 119-125.
